# Transcriptome profiling of grapevine seedless segregants during berry development reveals candidate genes associated with berry weight

**DOI:** 10.1186/s12870-016-0789-1

**Published:** 2016-04-26

**Authors:** Claudia Muñoz-Espinoza, Alex Di Genova, José Correa, Romina Silva, Alejandro Maass, Mauricio González-Agüero, Ariel Orellana, Patricio Hinrichsen

**Affiliations:** Instituto de Investigaciones Agropecuarias, INIA-La Platina, Santa Rosa 11, 610 Santiago, Chile; Centro de Biotecnología Vegetal, Universidad Andrés Bello, Av. Repúbica 217, Santiago, Chile; Center for Mathematical Modeling (UMI2807-CNRS) and Department of Mathematical Engineering, Faculty of Mathematical and Physical Sciences, University of Chile, Av. Blanco Encalada 2120, 7th Floor, Santiago, Chile; Center for Genome Regulation, Av. Blanco Encalada 2085, 3rd floor, Santiago, Chile

**Keywords:** RNA-seq, Table grapes, Berry weight, Functional genomics, Candidate genes

## Abstract

**Background:**

Berry size is considered as one of the main selection criteria in table grape breeding programs. However, this is a quantitative and polygenic trait, and its genetic determination is still poorly understood. Considering its economic importance, it is relevant to determine its genetic architecture and elucidate the mechanisms involved in its expression. To approach this issue, an RNA-Seq experiment based on Illumina platform was performed (14 libraries), including seedless segregants with contrasting phenotypes for berry weight at fruit setting (FST) and 6–8 mm berries (B68) phenological stages.

**Results:**

A group of 526 differentially expressed (DE) genes were identified, by comparing seedless segregants with contrasting phenotypes for berry weight: 101 genes from the FST stage and 463 from the B68 stage. Also, we integrated differential expression, principal components analysis (PCA), correlations and network co-expression analyses to characterize the transcriptome profiling observed in segregants with contrasting phenotypes for berry weight. After this, 68 DE genes were selected as candidate genes, and seven candidate genes were validated by real time-PCR, confirming their expression profiles.

**Conclusions:**

We have carried out the first transcriptome analysis focused on table grape seedless segregants with contrasting phenotypes for berry weight. Our findings contributed to the understanding of the mechanisms involved in berry weight determination. Also, this comparative transcriptome profiling revealed candidate genes for berry weight which could be evaluated as selection tools in table grape breeding programs.

**Electronic supplementary material:**

The online version of this article (doi:10.1186/s12870-016-0789-1) contains supplementary material, which is available to authorized users.

## Background

Grape (*Vitis vinifera* L.) is the main fruit crop of temperate regions, reaching nearly 77 million tons of fruit produced throughout the world in 2013 [1]. It also exhibits a high level of genetic diversity; the genus *Vitis* includes more than 50 species [2–4].

Berry weight is considered as one of the main selection criteria in table grape breeding, due to the consumer preferences for large and seedless berries along with organoleptic quality traits such as flavor and aroma [5]. However, berry weight is a quantitative and polygenic trait, probably determined by numerous processes such as cell multiplication, cell wall modification, water and sugar transport. Despite its relatively high heritability which is mostly additive, the genetic determination of berry weight was until recently scarcely documented [6, 7]. Therefore, considering the economic importance of berry weight for table grapes, it is relevant to determine its genetic architecture and elucidate the mechanisms involved in the expression of its driver genes. This information is required for the development of new cultivars involving the combination of desirable traits, which include not just berry size and lack of seeds, but also cluster architecture compatible with a proper berry spatial distribution [8], response to gibberellic acid (GA_3_) [9], yield [10] and tolerance to fungal diseases [11, 12], among others production traits.

As in other plant species, growth and cell proliferation of grape berries correspond to different processes which together determine the final fruit dimensions [13]. The development and maturation of grapevine berries has been studied as a model because of the uniqueness of this process in plant biology and its molecular regulation [14, 15].

Berry development presents a characteristic double sigmoid curve with three main phases, encompassing a series of physical and biochemical changes such as cell division and elongation, primary and secondary metabolism and resistance/susceptibility to biotic/abiotic stress [16]. Phase I involves events associated with cell division and cell elongation [17], the latter based on the accumulation of organic acids into the vacuole [6, 14, 18]. In this stage the berry is hard, green and grows slowly [14]; malic acid is the predominant metabolite. In Phase II, slower growth is observed and berry softening begins; numerous changes occur associated with gene expression and berry physiology reprogramming. Phase III is when berries reach their mature weight. This stage is characterized by the onset of sugar accumulation, a decrease in organic acid content and concomitantly, accumulation of anthocyanins in colored cultivars and volatile secondary metabolites associated with flavor and aroma [14].

A positive correlation has been described between the final berry weight and seed content [19] in segregating populations [20–24], possibly being the result of growth regulators produced by seeds [6, 25]. Interestingly, in stenospermocarpic varieties pollination occurs normally although the embryo development process aborts early, approximately 2 to 4 weeks after fertilization, while berry development continues normally [5, 24]. However, seedless varieties such as cv. Sultanina exhibit a reduced berry weight at harvest [26, 27], requiring two or three exogenous applications of gibberellic acid along with cluster thinning in order to maximize the potential berry growth; both practices demand high labor force, which increases production costs.

In relation to hormonal regulation, ethylene, auxins, ABA, cytokinins and gibberellins can influence berry development and ripening [28]. The concentration of auxins, cytokinins and gibberellins tends to increase during Phase I, in pre-*véraison* stages, and later decreasing in *véraison*, where a peak of abscisic acid has been described [28, 29].

Previous studies have described QTLs associated with berry weight in chromosomes 1 and 12 [23], 5 and 13 [30], 8, 11 and 17 [6], 15 [21] and 18 [22, 24]. In addition, [31] recently reported the *VvCEB1* gene, a bHLH transcription factor, as possibly involved in the regulation of cell size in cv. Cabernet Sauvignon. Also, the *VvNAC26* gene has been proposed as probably associated with berry weight variation in *V. vinifera* [32]. However, the genetics and information on the molecular mechanisms behind berry development in table grapes are still scarce and limited.

Diverse transcriptome studies based on microarrays [16, 33–35] as well as high-throughput RNA-Seq sequencing [36, 37] have been developed in grapes, focused on understanding the developmental and maturation process of the berry. However, these studies were directed to improve the understanding of organic acids, resveratrol, anthocyanin and tannin content and metabolism in relation to wine quality [36–40].

Due to the economic importance of berry weight in table grapes, it is relevant to determine the underlying mechanisms controlling this trait, in order to reveal positive and negative genetic factors involved in the expression of this complex trait.

We carried out the first transcriptome analysis with the aim of elucidating the mechanisms involved in berry weight determination. We contrasted seedless table grape segregants with opposite phenotypes for this trait in order to explore its genetic architecture. This comparative transcriptome profiling revealed candidate genes associated with berry weight, which could be evaluated as selection tools in table grape breeding programs.

## Results and Discussion

### RNA isolation from contrasting segregants for berry weight and library construction

The feasibility of this study was based on the availability of seedless segregants for berry weight (RxS crossing), maintained under the same climatic and agronomic conditions, which offer a unique opportunity to analyze transcriptome changes associated with this complex trait.

**Fig. 1:**
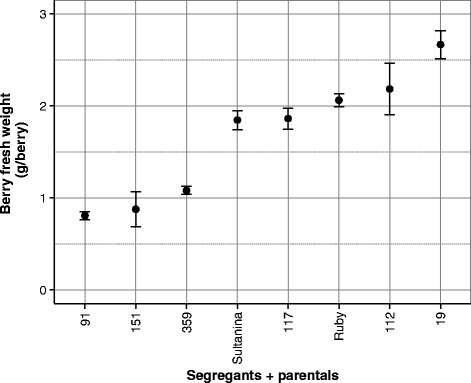
Berry fresh weight at harvest (18°Brix) of six RxS segregants exhibiting contrasting phenotypes, including parents cv. Ruby Seedless and Sultanina. Each value corresponded to phenotypic mean values during the 2009–2010, 2010–2011 and 2011–2012 seasons. Error bars represent one standard error of the mean (SEM)

In order to study the underlying differences between large and small berries, six seedless segregants derived from a ‘Ruby Seedless’ x ‘Sultanina’ crossing (RxS; *n* = 139) with contrasting phenotypes for berry weight were selected and phenotyped during three seasons, 2009–2010 to 2011–2012 (Fig. 1, Additional file 1: Table S1). According to ANOVA, the genotype effect was the most significant (83 %), the season effect corresponding to 8.5 % and the genotype x season interaction was 5.9 %. The linear model explained 97 % of the phenotypic variance (Table 1).

**Table 1:** Genotypic and season effect on berry weight phenotype (%)

Segregant	Season	Interaction	Model
82.86***	8.52*	5.93*	97.32

Significance codes according to ANOVA (p): ***0–0.001; **0.001–0.01; *0.01–0.05; *n.s.* not significant (*p* > 0.05). Coefficient of determinations (adjusted) based on mean squares of each factor, error and model according to ANOVA

**Fig. 2:**
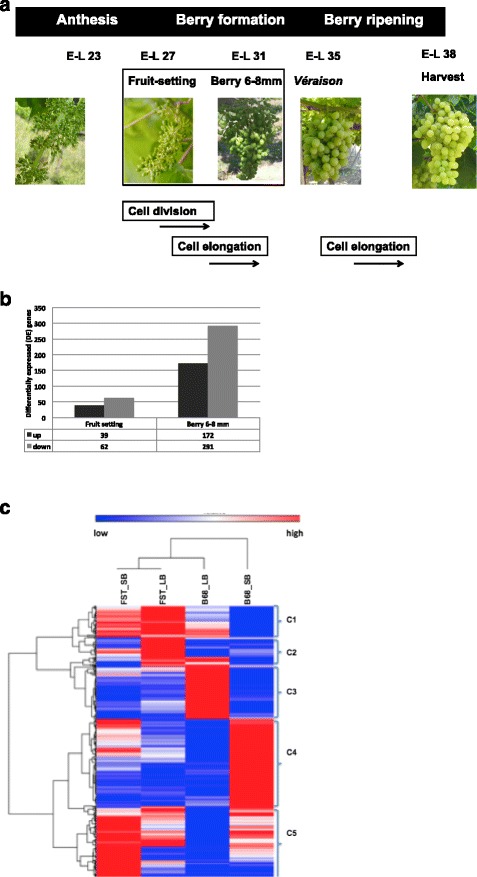
Experimental design, gene differential expression and hierarchical clustering of differentially expressed genes. **a** Phenological stages considered for the transcriptomic study. RNA samples were obtained from large (LB) and small (SB) berry genotypes, at phenological stages of fruit-setting (FST) and berry 6–8 mm stages (B68), modified from [15]. **b** Differentially expressed genes after comparison between RxS segregants with contrasting phenotypes for berry weight in both phenological stages. **c** Hierarchical clustering of a group of 526 differentially expressed genes among LB and SB segregants in the FST and B68 stages. Pearson correlation was used as distance and five clusters were identified

Thus a transcriptome experiment based on Illumina platform (RNA-Seq) was undertaken focused on early stages of berry development, i.e., fruit setting (FST) and berry 6–8 mm stages (B68) [14]; mRNA samples isolated in both stages were sequenced independently (Fig. 2a). These two stages are part of Phase I of the double sigmoid curve during berry growth, when the final number of cells is being defined, followed by cell expansion associated with water and organic acid accumulation in the vacuole [6, 14], critical processes defining the final fruit size [18, 31]. During the FST stage the berry cell machinery is receptive to exogenous gibberellin (GA) applications, increasing berry weight and reducing seed weight [41]. GA_1_ and GA_4_, the two endogenous bioactive GAs synthetized in the berry, have their maximum peaks in the FST and B68 stages, respectively (Ravest et al., in preparation).

### Illumina GAII mRNA sequencing

A total of 14 libraries were analyzed; 155,060,882 reads of 50 bp were obtained (Additional file 2: Table S2). After quality trimming 152,897,297 reads were kept, representing a loss of about 2 % of the reads for each library (Additional file 2: Table S2). Of this total, 91 % of the reads were mapped as unique and multiple alignments (Additional file 3: Table S3). The total of mapped reads corresponded to 147.8 million reads, of which 63 to 69 % mapped in exons, 15 to 19 % in UTR regions, 8 to 9 % within intron regions, and 6 to 9 % in intergenic regions; the percentage of usable reads (UTR and exons) varied from 80 to 85 % (Additional file 4: Table S4). A total of 8.5 million reads obtained from the 14 libraries were not mapped to the reference genome PN40024. They were used to construct 2,625 de novo contigs, with an average length of 673 bp. Of them, 457 contigs were mapped to the reference genome and reanalyzed (Additional file 5: Table S5).

### Global analysis of gene expression changes from fruit set (FST) to berry 6–8 mm (B68) stages

To determine which genes are changing their expression profiles and at what stage, comparisons between individuals with contrasting phenotypes for berry weight were performed (Fig. 2b, c). A group of 526 differentially expressed genes (DE) was identified comparing large (LB) versus small berry (SB) segregants in the two phenological stages (cuffdiff2 *p* < 0.01, FDR < 0.05) (Fig. 2b). In particular, 101 genes were identified from FST (39 up-regulated/62 down-regulated) (Additional file 6: Table S6) and 463 genes from B68 (172 up-regulated/291 down-regulated) (Additional file 7: Table S7). Interestingly, 37 of these were differentially expressed in both stages, with 34 coincidentally raising or decreasing their expression level, including transcripts coding for stilbene synthases (STS) (14) (Additional file 8: Table S8); this is equivalent to what has been observed in previous transcriptome studies during berry development in cv. Corvina [36] and cv. Cabernet Sauvignon [39].

A hierarchical clustering was performed using gene expression measured as fpkm observed in the group of 526 DE genes (Fig. 2c), and using Pearson correlation as distance in the transcriptional dendrogram. According to the expression profiles, five groups of DE genes were identified containing 60, 58, 101, 169 and 138 DE genes (Fig. 2c). In addition, a functional enrichment analysis (Gene Ontology) was developed to assess main processes over-represented in each cluster of transcripts using the agriGO platform [42] (Additional file 9: Figure S1). No over-represented category was identified in the case of cluster 2. Concomitantly, GO analysis of the groups of 101 DE genes identified in the FST and 463 in the B68 stage were performed and the results agreed with the global analysis.

### Functional analysis of DE genes comparing large and small berry segregants at fruit set (FST) and berry 6–8 mm (B68) development stages

#### Selection of a subset of candidate genes able to explain the difference in berry size

**Fig. 3:**
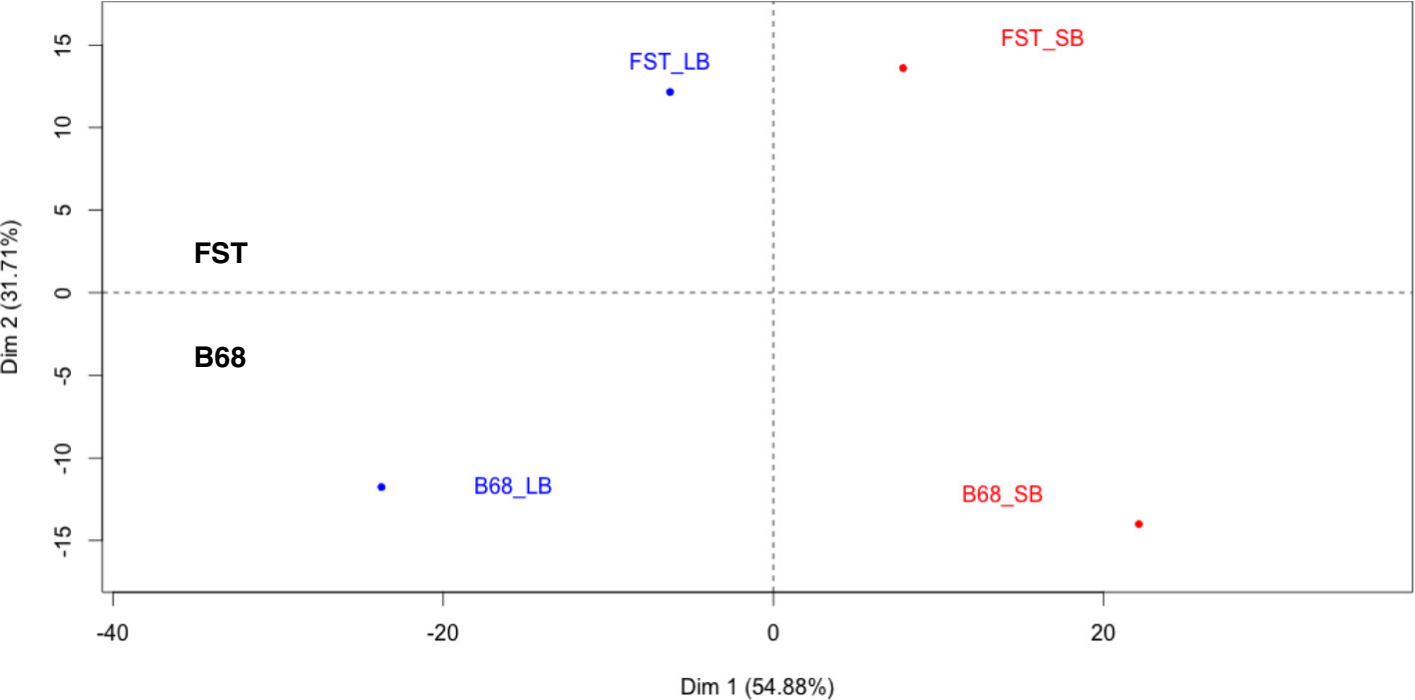
Principal components analysis (PCA) using normalized expression data (fpkm). Analysis included the group of 526 DE genes derived from comparison between LB (in blue) and SB segregants (in red) in the FST and B68 stages

In order to identify the genes involved in berry size determination, a principal components analysis was performed considering the 526 DE genes. The results showed that two components explained 87 % of the phenotypic variance (Fig. 3). The first component explained 55 % of the variation and clearly discriminates between contrasting phenotypes. The second component explained 31.7 % of the observed variation and discriminated between phenological stages (Fig. 3). Subsequently, correlation analyses were performed and significant correlations (*p* < 0.05) between DE genes and the two components were performed in order to select candidate genes, defined as transcripts whose expression level discriminates between individual classes [40].

A group of 68 DE genes were significantly correlated with component 1 and 16 with component 2 (Table 2). Interestingly, both subsets of DE genes were identified in the B68 stage (Additional file 7: Table S7).

**Table 2:** Differentially expressed genes (DE genes) significantly correlated with PCA components 1 (A) and 2 (B)

Category	geneID	Description	Correlation	*p*-value
A.				
Secondary metabolism				
	GSVIVG01027145001	O-acyltransferase WSD1	1.00	0.00
	GSVIVG01022205001	Cytochrome P450 84A1	0.99	0.01
	GSVIVG01036583001	Probable cytochrome P450 313a3	0.98	0.02
	GSVIVG01010574001	Stilbene synthase 4	0.95	0.05
Cell wall metabolism				
	GSVIVG01031543001	Lichenase	1.00	0.00
	GSVIVG01020228001	Probable xyloglucan endotransglucosylase/hydrolase protein 33	0.99	0.01
	GSVIVG01006161001	Glycogenin-2	0.97	0.03
	GSVIVG01011500001	Probable galacturonosyltransferase 13	0.96	0.04
	GSVIVG01029411001	Expansin-A15	−0.99	0.01
Water transport				
	GSVIVG01014205001	Epidermis-specific secreted glycoprotein EP1	0.99	0.01
Protein degradation/proteasome				
	GSVIVG01023803001	F-box protein At2g16365	0.96	0.04
	GSVIVG01007961001	LON peptidase N-terminal domain and RING finger protein 1	0.96	0.04
	GSVIVG01022680001	Protease Ulp1 family	−0.98	0.02
Hormonal metabolism and signaling				
	GSVIVG01035051001	Two-component response regulator ARR1	1.00	0.00
	GSVIVG01000579001	Vegetative incompatibility protein HET-E-1	0.96	0.04
	GSVIVG01008850001	Two-component response regulator ARR9	−0.97	0.03
Protein modification/kinase				
	GSVIVG01005164001	Cysteine-rich receptor-like protein kinase 29	0.99	0.01
	GSVIVG01015298001	Receptor-like protein kinase HSL1	0.99	0.01
	GSVIVG01013279001	Phosphatidylinositol-4-phosphate 5-kinase 5	0.98	0.02
	GSVIVG01005168001	Cysteine-rich receptor-like protein kinase 10	0.97	0.03
	GSVIVG01014382001	5'-AMP-activated protein kinase gamma subunit	0.97	0.03
	GSVIVG01023804001	AMP-activated protein kinase gamma regulatory subunit putative	0.97	0.03
	GSVIVG01021407001	LRR receptor-like serine/threonine-protein kinase FLS2	0.96	0.04
Stress/Defense				
	GSVIVG01019840001	Thaumatin-like protein	0.99	0.01
	GSVIVG01035061001	Major allergen Pru av 1	0.99	0.01
	GSVIVG01023740001	Protein WAX2	0.98	0.02
	GSVIVG01021355001	Protein SRG1	0.98	0.02
	GSVIVG01009107001	Cationic peroxidase 1	0.97	0.03
	GSVIVG01019841001	Pathogenesis-related protein R major form	0.97	0.03
	GSVIVG01019835001	Thaumatin-like protein	0.96	0.04
	GSVIVG01016196001	Nodulin family protein	0.96	0.04
	GSVIVG01008094001	Germin-like protein subfamily T member 1	0.96	0.04
	GSVIVG01016697001	18.6 kDa class III heat shock protein	−0.95	0.05
	GSVIVG01003320001	Cysteine proteinase inhibitor 1	−0.95	0.05
	GSVIVG01003118001	Heat stress transcription factor A-2b	−0.96	0.04
	GSVIVG01029025001	Chaperonin CPN60-1 mitochondrial	−0.96	0.04
	GSVIVG01016053001	Anthranilate N-benzoyltransferase protein 2	−0.96	0.04
	GSVIVG01000021001	Copper chaperone	−0.97	0.03
	GSVIVG01011742001	10 kDa chaperonin	−0.98	0.02
	GSVIVG01035433001	17.9 kDa class II heat shock protein	−0.99	0.01
	GSVIVG01024050001	Pathogenesis-related protein 5	−1.00	0.00
Development				
	GSVIVG01015278001	emb|CAB79689.1| putative protein	0.98	0.02
	GSVIVG01008595001	Protein RUPTURED POLLEN GRAIN 1	0.97	0.03
Chlorophyll biosynthesis				
	GSVIVG01008851001	Delta-aminolevulinic acid dehydratase chloroplast	−0.98	0.02
	GSVIVG01021406001	Chlorophyll a-b binding protein type 2 member 1B chloroplast	−0.97	0.03
Transport				
	GSVIVG01027803001	Inorganic phosphate transporter 1-4	1.00	0.00
	GSVIVG01029349001	Probable metal-nicotianamine transporter YSL7	0.99	0.01
	GSVIVG01000580001	ABC transporter B family member 15	0.96	0.04
	GSVIVG01034463001	ABC transporter G family member 25	0.95	0.05
	GSVIVG01001036001	Sugar carrier protein A	−0.96	0.04
	GSVIVG01033414001	Putative mitochondrial 2-oxoglutarate/malate carrier protein	−0.96	0.04
Transcription				
	GSVIVG01015353001	Transcription factor bHLH68	0.99	0.01
	GSVIVG01030127001	Zinc finger protein CONSTANS-LIKE 9	0.99	0.01
	GSVIVG01007666001	DEAD-box ATP-dependent RNA helicase 30	0.99	0.01
	GSVIVG01013182001	NAC domain-containing protein 78	0.98	0.02
	GSVIVG01017714001	Transcription factor HY5-like	0.96	0.04
	GSVIVG01003118001	Heat stress transcription factor A-2b	−0.96	0.04
	GSVIVG01024694001	GCN5-related N-acetyltransferase (GNAT) family protein	−0.96	0.04
B.				
Secondary metabolism				
	GSVIVG01021978001	Bifunctional 3-dehydroquinate dehydratase/shikimate dehydrogenase chloroplast	0.96	0.04
Cell wall metabolism				
	GSVIVG01028042001	Endoglucanase 1	0.95	0.05
	GSVIVG01036543001	Pollen Ole e 1 allergen and extensin family protein	0.96	0.04
	GSVIVG01037059001	Serine carboxypeptidase-like 18	0.96	0.04
Hormonal metabolism and signaling				
	GSVIVG01017158001	Auxin-induced protein AUX22	0.96	0.04
	GSVIVG01028033001	Indole-3-acetic acid-induced protein ARG2	0.96	0.04
	GSVIVG01037758001	Pirin-like protein	0.97	0.03
Stress/Defense				
	GSVIVG01009743001	Dihydroflavonol-4-reductase	0.97	0.03
Development				
	GSVIVG01020682001	Os01g0614300	0.98	0.02
	GSVIVG01009155001	Aspartic proteinase nepenthesin-1	0.97	0.03
	GSVIVG01034174001	Metallothionein-like protein type 2	0.97	0.03
	GSVIVG01036671001	Aspartic proteinase nepenthesin-1	0.95	0.05
Transcription				
	GSVIVG01037572001	Uncharacterized basic helix-loop-helix protein At1g64625	0.95	0.05

One of the most relevant functional categories associated with this group of genes was stress/defense response (26 %), encompassing HSP and chaperonins up-regulated in LB segregants (Additional file 10: Figure S2). In addition, protein kinase modifications and transcription categories were also relevant, possibly associated with the reprogramming of genes controlling transcription and translation rate in order to remodel the set of cell proteins. Four genes coding for receptor kinase-like (RLK) were up-regulated in SB segregants (Table 2). RLKs play a pivotal role in sensing external stimuli, activating downstream signaling pathways and regulating cell behavior involved in response to pathogens [43] growth and development processes in plants as well as biotic and abiotic stresses, suggesting a possible participation in the defense response in plants [43, 44]. This evidence suggests that a transcriptome reprogramming process is taking place during berry maturation, involving changes in synthesis and activation of proteins, processes that have been previously described in cv. Corvina, as well as a possible compensatory adaptation [16]. Indeed, increments in HSPs and chaperonin expression towards *véraison* have been reported, with a peak at *véraison* and subsequent reduction during berry maturation, associated with massive changes in metabolism at this phenological stage which demand the synthesis of new proteins [38, 45, 46].

Considering the observed evidence from other genetic backgrounds such as cv. Corvina, the higher expression level of HSP and chaperonins in LB segregants may be reflecting the adaptation of the berry to environmental stresses such as higher temperatures in the field.

Furthermore, evidence of a strong transcriptional control was found, with seven genes associated with the transcription category, two of them up-regulated in LB segregants, the heat stress transcription factor A-2b and a GCN5-related N-acetyltransferase (GNAT) family protein. Interestingly, the former corresponds to a transcriptional regulator whose orthologue in rice is the protein OsHsfA2e, induced by heat stress and specifically bound to the promotor of heat shock elements and possibly responsible for tolerance to high temperatures. Considering this, its introgression could be considered useful, in order to improve crop tolerance to climate change-associated stresses [47, 48]. The latter gene, a histone acetyltransferase (HAT), is responsible for lysine residue acetylation in histones H2B, H3 and H4, and also acts as a transcriptional activator, implicated in chromatin assembly and DNA replication [49].

In addition, a gene coding for a NAC domain-containing protein 78 was found up-regulated in SB segregants, which are plant-specific transcription factors (TFs). Members of this gene family have been related to plant development [50]. In particular in *Vitis vinifera, VvNAC26* gene has been associated with the early development of grape flowers and berries [51], possibly contributing to berry size variation [32].

In the transport category six DE genes were found, two up-regulated in LB segregants, the sugar carrier protein A and the putative mitochondrial 2-oxoglutarate/malate carrier protein, probably associated with the transport of malate to the vacuole and cell turgor; both could be key for cell expansion. Malate is the main organic acid stored in the vacuole of grape berry cells, from FST to *véraison* [46].

Associated with cell wall metabolism, we found DE genes coding for a probable xyloglucan endotransglucosylase/hydrolase proteins, a lichenase and a probable galacturonosyltransferase 13, up-regulated in SB segregants, and an expansin-A15, up-regulated in LB segregants (Additional file 7: Table S7). This result is concordant with the top over-represented category ‘xyloglucan:xyloglucosyl transferase’ associated with cluster 4 (Fig. 2c, Additional file 9: Figure S1C).

This evidence could be related to cell expansion events described in the B68 stage, which initially requires cell wall softening and later the incorporation of recently synthetized material [18, 31]. Cell wall softening occurs as a result of disruption of chemical bonds between structural cell wall components, by acidification and hydrolase enzymes, modifications which require an accurate and coordinated transcriptional regulation of genes involved in biosynthesis and cell wall adaptations [18, 31]. These enzymes modify hemicelluloses during cell expansion and fruit softening, suggesting a direct influence on growth. Furthermore, cell expansion involves changes in composition as well as the accumulation of different compounds which maintain osmotic pressure and water flux in cells in expansion [31, 52]. Evidence obtained in this study agreed with these events where a strong induction of genes associated with cell expansion was observed, which probably results in larger berry weights.

Our results suggest a relevant role of expansins in the LB phenotype during the B68 stage. In the case of SB segregants, genes with xyloglucan:xyloglucosyl transferase activity were found up-regulated in the same stage (Additional file 7: Table S7). This evidence suggests a differentiation in cell wall modifications, considering that expansins have been proposed as cell wall activator agents without hydrolytic activity. Likewise, up-regulated endoglucanases were identified in LB segregants, which are also associated with cell wall dynamics. Concomitantly, in the B68 stage genes related to auxin metabolism were also identified, up-regulated in the LB phenotype, in line with the putative role of auxins in cell expansion, involved in acid growth mediated by expansins [31, 53] (Additional file 7: Table S7).

Evidence obtained from the transcriptome analysis suggested that major differences among LB and SB seedless segregants are triggered at the B68 stage, which may be responsible for the final berry weight observed at harvest. In this stage berry diameter increases by cell expansion [14].

Other functional categories were associated with secondary metabolism, transport of inorganic ions and metals, proteosome-protein degradation, hormone metabolism and signaling, development and chlorophyll biosynthesis (Additional file 10: Figure S2).

Regarding the group of 16 genes significantly correlated with component 2 (Table 2), two genes were identified coding for aspartic proteinase nepenthesin-1, possibly associated with aspartic-type endopeptidase activity [54], and senescence process (development); as well as a serine carboxypeptidase-like 18 and endoglucanase 1, both related to cell wall metabolism (Additional file 11: Figure S3). Furthermore, three genes were found associated with hormonal metabolism and signaling, coding for auxin-induced protein AUX22 and ARG2, and pirin-like protein, related to calcium signaling.

### Co-expression network analysis

**Fig. 4:**
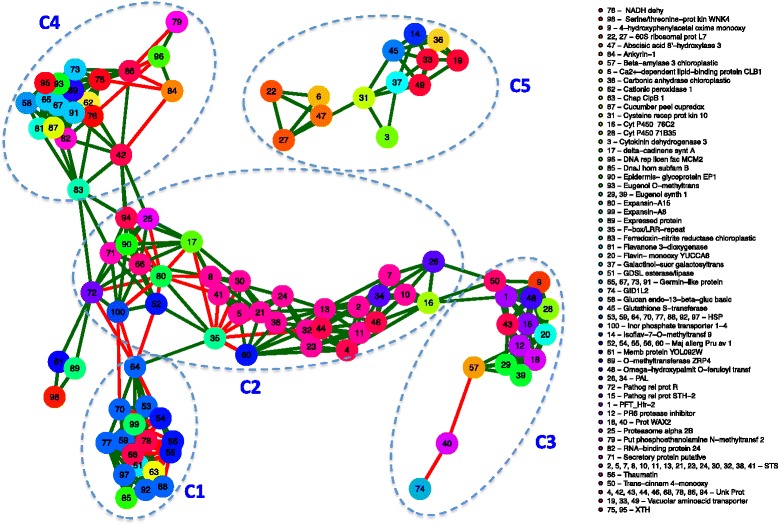
Nodes of co-expressed genes among LB and SB segregants identified using a network analysis. Main components of each node are N1: HSPs, chaperonins; N2: STBs, thaumatins; N3: monooxygenases; N4: cell wall modifications; N5: vacuolar transporters. Lines in red and green represent negative and positive correlations, respectively

Network analyses were performed to identify co-expression genes associated with the separation between LB and SB segregants. Subsequently, correlation analyses results lead to identify a total of 4,950 partial correlations, 431 of them significant (*p* < 0.05). Correlograms were plotted with the total observed correlations (Additional file 12: Figure S4), and correlations of over 90 % were considered as significant (Additional file 13: Figure S5). Furthermore, 15 % of the significant correlations were negative and more variable (CV = 5 %). Positive significant correlations represented 85 % and were less variable (CV = 2.6 %). Five interconnected clusters of nodes were identified (Fig. 4) (Additional file 14: Table S9).

These results were concordant with those obtained from hierarchical clustering and PCA; the seven DE genes selected as candidate markers for berry weight from PCA analysis were also present in the network analysis (Additional file 15: Figure S6). In addition, according to the cluster connectivity our results agreed with previous studies which described that highly connected genes were usually involved in the same biological pathways [55].

Cluster one was conformed mostly of genes coding for HSPs and chaperonin proteins, including also a gene coding for GDSL esterase/lipase and expansin-A8 (Fig. 4), all of them up-regulated in LB segregants. This result is concordant with identification of the category ‘Protein folding’ over-represented in cluster 3 (Fig. 2c, Additional file 9: Figure S1B), a process mediated by HSP [56]. As these genes have been associated with heat stress during berry development [56] and the response to microclimate changes in bunches [16, 57], this evidence suggests that LB segregants could respond more efficiently to heat stress.

Negative correlations were among genes coding for major allergen Pru av 1, associated with defense responses [58, 59], and expansin-A8; genes coding for chaperonins or HSPs were also found (Fig. 4).

Cluster two was composed mainly of genes coding for PALs and STS, a gene co-expression previously reported in cv. Syrah [37]; they were up-regulated in SB segregants in this study, in both phenological stages (Fig. 4; Additional files 6 and 7). These results are concordant with the identification of the over-represented categories ‘L-phenylalanine catabolic process’ and ‘Response to biotic stimulus’ found in cluster 5 (Fig. 2c, Additional file 9: Figure S1D, E).

STS expression has been considered as a response to stress factors such as fungal diseases, wounding and UV light [16, 60, 61], and a shift in phenylpropanoid pathway metabolites is highly sensitive to temperature changes [56]. The differential expression of those genes during berry development and maturation have been described in cv. Corvina [16, 40, 62], Norton [33] and Moscatel de Hamburgo [35]. Hence these results suggest that SB segregants presented a higher stress level during berry development than LB segregants, possibly environmental due to high temperatures.

However, positive correlation was observed between genes coding for expansin-A15 (code 80) and F-box/LRR-repeat protein 3 (code 35), both negatively correlated with genes coding for stilbene synthases in the cluster. F-box proteins act as regulators of the ubiquitin kinase dependent pathway associated with protein degradation, an important post-translational mechanism. Thus the removal of unfolded or non-functional proteins facilitates the adaptation of organisms to environmental changes, through rapid intracellular signaling [63].

In particular, expansin-A15 also showed negative correlation with genes coding for thaumatins, proteosome subunits, inorganic transporters and proteins related to pathogenesis (Additional file 14: Table S9), identified up-regulated in SB segregants (Additional files 6 and 7).

Cluster three was composed mostly of genes with monooxygenase and oxide-reductase activities, including cytochrome P450, PR6 protease inhibitor and eugenol synthase (Fig. 4). Genes belonging to the cytochrome P450 family were found up-regulated in SB segregants (Additional files 6 and 7), associated with phenylpropanoids, flavonoids, brassinosteroids and lignin synthesis. Interestingly, it has been reported that cytochrome P450-78A partially controls fruit size in tomato and possibly has a role in the domestication of this species [64].

Biosynthetic enzymes, redox regulators and HSP have been described as effector genes related to abiotic stress responses [65]. However, genes coding for chloroplast beta-amylase 3, gibberellin receptor GID1 and protein WAX2, up-regulated in SB segregants, were negatively correlated (Fig. 4).

WAX2 protein plays a role in the conversion or secretion of common precursors for cutins and wax metabolic pathways; it is also related to cuticle formation and stomata, both involved in transpiration control and drought tolerance as well [66].

Cluster 4 included a cohort of candidate enzymes related to cell wall modification, with xyloglucan endotransglucosylase/hydrolase protein 23 (XTH) and glucan endo-13-beta-glucosidase activities, positively correlated (Fig. 4).

Interestingly, cluster 5 presented no edges with the remaining clusters. Two branches were observed, the first composed of genes coding for 60S ribosomal protein L7 and abscisic acid 8'-hydroxylase 3, all of them positively correlated. The ribosomal protein modulation suggests that the transcriptome reprogramming that occurs during berry maturation involves changes in protein synthesis [16] (Fig. 4). A second branch included genes coding for cysteine-rich receptor-like protein kinase 10; vacuolar amino acid transporter 1, up-regulated in LB segregants (Additional file 6: Table S6), possibly associated with amino acid compartmentalization in the vacuole [67]; cytokinin dehydrogenase 3, as well as galactinol-sucrose galactosyltransferase; glutathione S-transferase, associated with the cellular response induced by heat shock stress and auxins, and metals such as cadmium, silver and copper [68]; and isoflavone-7-O-methyltransferase 9, related with flavonoid/isoflavonoid metabolism and biotic stress responses [69], which were positively regulated (Fig. 4).

### Expression analysis of a group of candidate genes associated with berry weight using qPCR

**Fig. 5:**
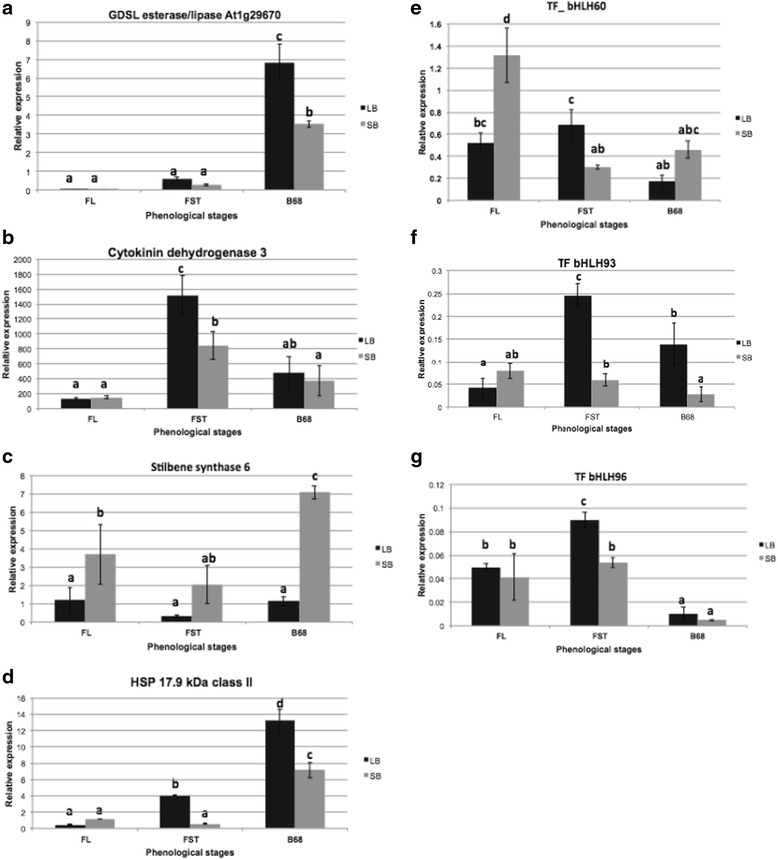
Validation of differentially expressed (DE) genes among LB and SB segregants by real-time PCR. LB, large berries, in black; SB, small berries, in grey. Phenological stages were anthesis (FL), fruit setting (FST) and berry 6–8 mm (B68). Genes are **a** GDSL esterase/lipase; **b** cytokinin dehydrogenase 3; **c** stilbene synthase 6; **d** gene coding for 17.9 kDa class II HSP; **e** TF-bHLH60; **f** TF-bHLH93; **g** TF-bHLH96; different letters on top of bars indicate significant differences (*p* < 0.05) according to one-way ANOVA and Tukey’s multiple comparison test among phenotypic category/phenological stage; values are the results of 27 observations categorized by phenotype. The TCPb gene (possible T-complex protein subunit beta, GSVIVG01008708001) was used as reference gene and gene expression was expressed as relative expression

The expression profiles of seven DE genes were experimentally validated by real-time qPCR experiments, in the phenological stages of anthesis (FL), fruit-setting (FST) and berry 6–8 mm (B68) (Fig. 5), in order to select candidate genes as putative factors associated with berry weight determination.

The results of the network and PCA were considered in the selection of candidate genes. Genes coding for GDSL esterase/lipase, cytokinin dehydrogenase 3 and stilbene synthase 6 were selected from the network analysis. In addition, the gene coding for HSP 17.9 kDa class II was significantly correlated with PCA component 1.

In the case of the gene coding for GDSL esterase/lipase, experimental results confirmed its up-regulated expression in LB segregants in the B68 stage (*p* < 0.05), suggesting an increase in its expression in this stage in both LB and SB segregants (Fig. 5a). In addition, the gene coding for cytokinin dehydrogenase 3 was significantly up-regulated in LB segregants in the FST stage (Tukey test, *p* < 0.05), with lower expression in both groups of segregants at the B68 stage (Fig. 5b). Interestingly, the gene coding for stilbene synthase 6 showed a tendency to be up-regulated in SB segregants in the three evaluated stages. Significant differences were confirmed during FL and B68 (Tukey test, *p* < 0.05), being higher in the latter stage (Fig. 5c).

In addition, the gene coding for HSP 17.9 kDa class II (HSP17.9-D) showed similar expression in FL in LB and SB segregants. However, in the FST and B68 stages it was significantly up-regulated in LB segregants (Fig. 5d). Considering that HSP17.9-D was also highly correlated with component 1 of the PCA, it could be considered as a potential candidate gene for berry weight.

Participation of bHLH proteins in plant organ size determination has been described. In particular in *V. vinifera*, the cell elongation protein bHLH (*VvCEB1*) has been recently associated with berry weight in Cabernet Sauvignon, possibly involved in cell expansion during berry development [31]. Therefore, in order to evaluate the possible role of members of this transcription factor family in the differences between LB and SB segregants, three DE genes coding for bHLH60, bHLH93 and bHLH96 were selected to be experimentally confirmed by real-time qPCR experiments.

The results showed that in the case of genes coding for transcription factors (TFs), TF-bHLH60 was significantly up-regulated in SB segregants during the FL stage. However, in the FST stage it was up-regulated in LB segregants (*p* < 0.05) (Fig. 5e). There was an inflexion in the FST stage, with maximum expression for LB segregants and minimum for SB segregants. The same tendency was detected for TF-bHLH93, but with a significant differential expression during the FST and B68 stages, up-regulated in LB segregants (Fig. 5f). A similar expression profile was observed in the gene coding for TF-bHLH96, which was up-regulated in LB segregants at the FST stage (Fig. 5g).

Interestingly, our results differ from previous reports that proposed the gene *VvCEB1* as a candidate marker for berry size, whose transcripts are predominantly accumulated in berries, especially with minimum auxin content [31]. Indeed, the three TFs evaluated showed higher expression in the FST stage comparing LB vs. SB segregants, suggesting a possible role in early stages of development. Experimental validation in advanced phenological stages would confirm their expression profile in berries in order to determine if, as with *VvCEB1*, these TFs plays a role in cell expansion in a wider genetic background.

## Conclusions

We have carried out the first transcriptome analysis focused on seedless table grape segregants with contrasting phenotypes for berry weight. A group of 526 differentially expressed genes potentially associated with berry size was identified, 101 genes in the FST stage and 463 genes in the B68 stage.

The integration of differential expression, PCA, correlation and network analysis provided a wide characterization of overall regulation and dynamic remodeling of the gene expression in berry development in pre-*véraison* stages. A survey of candidate genes was also performed, and expression profiles of seven candidate genes were validated.

## Methods

### Plant material

The ‘Ruby’ x ‘Sultanina’ (RxS) population (*n* = 139) is planted at La Platina Experimental Station of the Instituto de Investigaciones Agropecuarias (INIA), located in Santiago, Chile (Latitude 33°34’23.3”S, longitude 70°37’35.73”W). This population is managed using a trendil system known as ‘spanish parron’, grafted over cv. Sultanina, and two to four replicates are available per segregant (clones). Segregants were managed under standard conditions for watering, fertilization, pest and diseases control and pruning. Both parents are publicly available resources and the segregants belong to the table grape breeding program of INIA.

The segregants and both parents were sampled in order to determine a number of quality-related traits; sugar content and titratable acidity, berry and seed weight and volume were the relevant traits for this study. Phenotype robustness was evaluated over three seasons (2009–2010 to 2011–2012), as well as health condition and vigor.

### Experimental design and sample collection

A group of six segregants of the RxS cross (*N* = 139), named 19, 27, 112, 117, 151 and 359, plus both parents, Ruby Seedless and Sultanina, were selected for transcriptome analysis. These segregants exhibited contrasting phenotypes (Fisher test, *p* < 0.05) for berry size and weight, i.e., small (SB) and large (LB), all of them seedless (Fig. 1). Berry samples were collected in the 2009–2010 season 30 and 45 days after flowering, in phenological stages of E-L 27 and E-L 31 [70, 71], corresponding to fruit setting (FST) and berries of 6–8 mm diameter (B68), considered as early stages of berry development. Each genotype was sampled in two or three replicates (clones), which were later considered as technical replicates. Samples were collected in the field, frozen in liquid N2 and stored at −80 °C until RNA extraction.

### RNA isolation from contrasting segregants for berry weight, library construction and mRNA sequencing

For RNA-Seq experiments, pericarp and mesocarp tissues were homogenized and analyzed together. Total RNA was isolated from 3 to 4 g of frozen tissue using the modified hot borate method [72]. The quantity and quality of RNA was assessed by measuring the A260/280 ratio using a Nanodrop ACT GeneASP-2680 equipment, and by agarose gel electrophoresis. RNA samples with 260/280 ratios between 1.8 and 2.2 were selected. Prior to sequencing, RNA integrity values were evaluated using a BioAnalyzer. Selected samples reported an RNA Integrity Number (RIN) ≥ 7.0. RNAs were sequenced after the corresponding cDNA synthesis, as described by [73]. Sequencing was performed using an Illumina sequencing platform (Genome Analyzer II) (IGA, Udine, Italy).

qPCR analysis followed the same RNA isolation protocol described above and cDNA were obtained by reverse transcription reactions with 2 ug of total RNA as template, using MMLV-RT reverse transcriptase (Promega, Madison, WI) and oligo dT primers according to standard procedures. The concentration of cDNA was assessed by measuring the absorbance at 260 nm, using a Nanodrop ACT Gene ASP-2680 equipment, finally diluting each cDNA to 50 ng/uL prior to use in qPCR experiments.

### Sequencing data analysis

A total of approximately 10 million single-end reads were obtained per sequenced library, with an average length of 50 bp. Reads were trimmed by sequencing quality (Q20) and a minimum length of 30 bp. Trimmed, good-quality reads were aligned to the grapevine reference genome (PN40024 12X.v1) [74] using Tophat software [75], with a maximum of two mismatches per read. Multiple reads with more than 20 hits were discarded. Reads were then normalized as fpkm expression values, defined as reads per kilobase of exon per million reads mapped, to make them comparable across experiments. The reference grapevine genome and the gene annotation were downloaded from the GENOSCOPE database [76]. The RNA-Seq data used in this study are available at the NCBI’s Sequence Read Achieve [77] with SRA Study accession number SRX366617 [73].

### Differential expression analysis

In order to identify differentially expressed genes, libraries derived from LB segregants (19, 112, and 117) were compared with SB segregant libraries (91, 151 and 359) in phenological stages FST and B68. Segregants exhibiting the same phenotype for berry weight were considered as biological replicates in the analysis [78]. Differential expression analysis was done using Cuffdiff2 (v. 2.0.2) software [79], using a geometric data normalization of library sizes (including replicates), multi-reads and fragment bias correction. Significant differences with *p* < 0.01 and a False Discovery Rate (FDR) of 0.05 were considered in this analysis.

### Cluster analysis and gene ontology assignment

Hierarchical clustering (HCL) was performed using Pearson’s correlation distance and GENE-E software [80]. A gene ontology (GO) enrichment analysis was performed considering 526 differentially expressed genes (DE genes) grouped in five clusters, obtained from comparison of LB vs. SB segregants in the FST and B68 stages. The frequency of query genes was compared with the complete reference genome for *V. vinifera* (PN40024), searching for possible enrichment in biological processes. Analyses were performed using agriGO tool [81], with the singular enrichment analysis and complete GO options. Significant GO terms (*p* < 0.05) were calculated using the hypergeometric distribution and the Yekutieli multi-test adjustment method [42].

### Principal components analysis

A principal components analysis with the group of 526 DE genes obtained from comparison of LB and SB segregants in the FST and B68 stages was performed using the FactoMineR library [82] and R statistical software [83]. Then, in order to identify candidate genes, i.e., transcripts whose absence, presence or expression level could be able to discriminate between segregants, a correlation analysis between DE genes and components 1 and 2 derived from PCA was performed. Thus significantly correlated DE genes (*p* < 0.05) were selected as candidate genes.

### Gene co-expression network analysis

To perform the network analysis a matrix of Pearson correlations was developed, based on average values for each phenotype, which was later represented in a correlogram using the corrplot library [84] and R software [83]. Subsequently, a partial correlation analysis was performed using the PCIT library [85] and R software. Significant correlations were plotted in a correlogram. Later, correlations were considered for a network analysis using R qgraph [86]. Network analysis consisted of the representation of correlations between variables in a set of nodes connected by edges, which showed the correlation between variables [87].

### Gene expression analysis by qPCR

Quantitative real-time PCR expression analysis (qPCR) of the seven selected genes was performed in the group of six segregants with contrasting phenotypes for berry weight, in the phenological stages of anthesis (FL), fruit-setting (FST) and berry of 6–8 mm (B68), corresponding respectively to E-L 23, E-L 27 and E-L 31 [70, 71]. qPCR were carried out using StepOne™Real-Time PCR System equipment (Applied Biosystems, Carlsbad, California). The qPCR amplification reactions were performed in a total volume of 10 μl containing 1 μL cDNA (50 ng/μL), 1 μL primer mix (from 400 nM to 600 nM depending on the gene), 5 μL FastStart Essential DNA Green Master (2X) (Roche, Mannheim, Germany), and 3 μL nuclease-free water. The thermal cycling conditions were denaturation at 95 °C for 10 min, followed by 40 cycles of template denaturation at 95 °C for 15 s, primer annealing at 60 °C for 1 min and extension at 72 °C for 25 s. The amplicon specificity was verified through melting curve analysis, 60 °C to 95 °C, with a gradient of 0.3 °C after 40 cycles. For each segregant three biological replicates (clones) were used, with three technical replicates per point. In addition, three segregants representative of SB or LB phenotypes were used per point, corresponding to a total of 27 observations. Values were normalized based on the housekeeping gene TCPb, which codes for a putative protein complex T subunit ß (GSVIVG01008708001) [73]. Statistical analysis of qPCR results involved ANOVA and Tukey tests (*p* < 0.05), and were performed using the statistical package Infostat (v2012) [88].

### Primer design

Specific primers for genes being analyzed were designed using PRIMER three software [89], according to parameters described by [90], and checked *in silico* using the Operon software [91]. Primers were synthesized by Integrated DNA Technologies, Inc. (Coralville, Iowa). The nucleotide sequences of the genes of interest were downloaded from a private database maintained at [92]. Primers used in real-time experiments (qRT-PCR) are summarized in Additional file 16: Table S10.

### Ethics approval and consent to participate

Not applicable.

### Consent for publication

Not applicable.

### Availability of data and material

The RNA-Seq data used in this study is available at the NCBI’s Sequence Read Achieve (http://www.ncbi.nlm.nih.gov/sra) with the SRA Study accession number SRX366617.

## Additional files

Additional file 1: Table S1. Phenotypical characterization of berry weight at harvest of six RxS segregants exhibiting contrasting phenotypes for berry weight, including parents cv Ruby Seedless and Sultanina. Analyses were performed during the 2009–2010 to 2011–2012 seasons. SEM is the standard error of the mean and CV is the coefficient of variation. (PDF 59 kb) Additional file 2: Table S2. Read quality summary considering the total 14 libraries. (PDF 46 kb) Additional file 3: Table S3. Read mapping summary. (PDF 54 kb) Additional file 4: Table S4. Read mapping distribution summary. (PDF 49 kb) Additional file 5: Table S5. Unmapped reads summary describing the total of contigs mapped to the reference genome using MegaBlast program. (PDF 57 kb) Additional file 6: Table S6. Differentially expressed (DE) genes identified in the comparison between LB and SB segregants, in the FST stage (Cuffdiff2, *p* < 0.01). (PDF 117 kb) Additional file 7: Table S7. Differentially expressed (DE) genes identified in the comparison between LB and SB segregants, in the B68 stage (Cuffdiff2, *p* < 0.01). (PDF 327 kb) Additional file 8: Table S8. List of 37 DE genes (*p* < 0.01) identified in the FST and B68 stages in the comparisons between LB and SB segregants. (PDF 69 kb) Additional file 9: Figure S1. A, B, C, D, E. GO enrichment of five clusters identified in hierarchical clustering of 526 DE genes, from comparison between LB and SB segregants. A. GO categories over-represented in Cluster 1 (Biological Process); B. GO categories over-represented in Cluster 3 (Biological Process); C. GO categories over-represented in Cluster 4 (Molecular Function); D. GO categories over-represented in Cluster 5 (Biological Process); E. GO categories over-represented in Cluster 5 (Molecular Function). Biological Process and Molecular Function categories are shown, and only significantly over-represented categories were considered (*p* < 0.05 and FDR < 0.05). The analysis was performed using the online agriGO tool and the GO complete category. The boxes contain the GO number, the p-value (in parentheses), the category description, the number of genes in each category associated with the GO term versus the total of query genes and the number of genes in each category out of 14,511 genes of the reference genome of *Vitis vinifera* (PN40024, 12X.v1), with associated GO terms. The arrows indicate the relationships among the GO categories, as follows: black solid arrows mean that a GO category is also included in the other one; red solid arrows mean that one GO category positively regulates the other; green solid arrows mean that the GO category negatively regulates the other; black dashed arrows indicate that there are two significant nodes related to the GO category; and black dotted arrows indicate that only one significant node is related to the GO category. (PDF 567 kb) Additional file 10: Figure S2. Functional characterization of 68 candidate genes significantly correlated with PCA component 1, associated with differences between LB and SB segregants. (PDF 96 kb) Additional file 11: Figure S3. Functional characterization of 16 candidate genes significantly correlated with PCA component 2, associated with differences between FST and B68 stages. (PDF 87 kb) Additional file 12: Figure S4. Correlogram representing a total of 4,950 partial correlations, including significant and non-significant correlations, found among the group of 100 DE genes with the highest significance, associated with differences between LB and SB segregants, in the FST and B68 stages. The color indicates the type of correlation i.e., negative significant correlations are in red while positive significant correlations are shown in blue. Intensity of colors indicates strength of correlations; darker shades represent higher or more negative values. (PDF 174 kb) Additional file 13: Figure S5. Correlogram representing 431 significant correlations (*p* < 0.05), found among the group of 100 DE genes with the highest significance, associated with differences between LB and SB segregants in the FST and B68 stages. Correlograms were plotted with the total of observed correlations. The color indicates the type of correlation i.e., negative significant correlations are in red while positive significant correlations are shown in blue. Intensity of colors indicates strength of correlations; darker shades represent higher or more negative values. (PDF 132 kb) Additional file 14: Table S9. Summary of significant partial correlations observed in the group of 100 DE genes with high significance, associated with differences between LB and SB segregants, in the FST and B68 stages. (PDF 295 kb) Additional file 15: Figure S6. Network analysis of co-expressed genes among LB and SB segregants. In blue and red are represented DE genes that are part of the network, which respectively present or not significant correlation with component 1 of the PCA analysis, selected as candidate genes for berry weight. Lines in red and blue represent negative and positive correlations, respectively. (PDF 64 kb) Additional file 16: Table S10. Primers designed for the analysis of the expression level of the seven candidate genes, based on real-time qPCR. (PDF 63 kb)

## Abbreviations

B68: berry 6–8 mm stage
DE: differentially expressed
FST: fruit set stage
GO: gene ontology
HSP: heat shock protein
INIA: Instituto de Investigaciones Agropecuarias
LB: large berry segregants
PAL: phenylalanine-ammonium lyase
PCA: principal components analysis
qPCR: Real-Time PCR
QTL: quantitative trait loci
SB: small berry segregants
STS: stilbene synthases
