# Supplementary material for: Transcriptome profiling of grapevine seedless segregants during berry development reveals candidate genes associated with berry weight
Source: BMC Plant Biol. 2016 Apr 26;16:104. doi: 10.1186/s12870-016-0789-1 (PMC4845426; doi:10.1186/s12870-016-0789-1)
Supplement: Additional file 1: Table S1. — Phenotypical characterization of berry weight at harvest of six RxS segregants exhibiting contrasting phenotypes for berry weight, including parents cv Ruby Seedless and Sultanina. Analyses were performed during the 2009–2010 to 2011–2012 seasons. SEM is the standard error of the mean and CV is the coefficient of variation. (PDF 59 kb) [file 12870_2016_789_MOESM1_ESM.pdf]

Table S1. Phenotypical characterization of berry weight at harvest of six RxS segregants exhibiting contrasting phenotypes for berry weight, including parents cv. Ruby Seedless and Sultanina. Analyses were performed during the 2009-2010 to 2011-2012 seasons. SEM is the standard error of the mean and CV is the coefficient of variation.

Segregant or parent	Season	Mean (gr)	SEM (gr)	CV (%)	Fisher Test*
91	2009-2010	0.74	0.03	11.0	c
151	2009-2010	1.03	0.54	117.6	c
359	2009-2010	1.10	0.07	20.3	c
117	2009-2010	1.72	0.10	16.4	b
Sultanina	2009-2010	1.78	0.20	31.3	b
Ruby	2009-2010	1.82	0.10	11.5	b
19	2009-2010	2.65	0.16	18.4	a
112	2009-2010	2.70	0.35	29.2	a
91	2010-2011	0.92	0.05	14.2	d
151	2010-2011	0.94	0.05	13.2	d
359	2010-2011	1.15	0.02	4.6	cd
112	2010-2011	1.57	0.37	52.9	c
117	2010-2011	2.17	0.29	30.1	b
Sultanina	2010-2011	2.18	0.04	4.3	b
Ruby	2010-2011	2.27	0.05	4.5	b
19	2010-2011	2.95	0.28	19.0	a
151	2011-2012	0.48	0.07	26.6	c
91	2011-2012	0.67	0.19	40.7	c
359	2011-2012	0.85	0.01	2.7	c
Sultanina	2011-2012	1.64	0.17	27.5	b
19	2011-2012	1.65	NA	NA	b
117	2011-2012	1.73	0.15	14.5	b
Ruby	2011-2012	2.04	0.09	7.9	ab
112	2011-2012	2.70	NA	NA	a

*Different letters indicate means ± standard error of the mean (SEM) with statistically significant differences among genotypes according to Fisher's least significant difference test (LSD, $p < 0.05$). NA: not available.
