# Supplementary material for: Transcriptome profiling of grapevine seedless segregants during berry development reveals candidate genes associated with berry weight
Source: BMC Plant Biol. 2016 Apr 26;16:104. doi: 10.1186/s12870-016-0789-1 (PMC4845426; doi:10.1186/s12870-016-0789-1)
Supplement: Additional file 2: Table S2. — Read quality summary considering the total 14 libraries. (PDF 46 kb) [file 12870_2016_789_MOESM2_ESM.pdf]

Table S2. Read quality summary considering the total 14 libraries.

Library	Number of raw reads	Number of trimmed reads	Usable reads (%)
SB_FST_91	7,784,105	7,706,202	98
SB_FST_151	9,372,771	9,180,595	97
SB_FST_359	12,941,624	12,740,412	98
LB_FST_19	8,927,416	8,822,155	98
LB_FST_112	10,885,400	10,728,881	98
LB_FST_117	9,406,990	9,358,954	99
Sul_FST	10,483,239	10,306,135	98
Ruby_FST	11,703,667	11,581,925	98
SB_B68_91	5,560,072	5,498,863	98
SB_B68_359	4,758,771	4,682,808	98
LB_B68_19	11,425,990	11,291,471	98
LB_B68_112	23,640,636	23,263,080	98
LB_B68_117	18,573,815	18,295,075	98
Sul_B68	9,596,386	9,440,741	98

FST= Fruit set stage; B68= Berry of 6-8 mm stage.

SB= Small berry segregant; LB= Large berry segregant.
