# Supplementary material for: Transcriptome profiling of grapevine seedless segregants during berry development reveals candidate genes associated with berry weight
Source: BMC Plant Biol. 2016 Apr 26;16:104. doi: 10.1186/s12870-016-0789-1 (PMC4845426; doi:10.1186/s12870-016-0789-1)
Supplement: Additional file 3: Table S3. — Read mapping summary. (PDF 54 kb) [file 12870_2016_789_MOESM3_ESM.pdf]

Table S3. Read mapping summary.

Library	Total alignments	Unique alignments	%	Multiple reads map	%	Exact alignments	%	Splice alignments	%	Total read map	%
SB_FST_91	7,669,285	7,026,509	91,6	251,374	3,3	6,468,777	84,3	1,200,508	15,7	7,277,883	94
SB_FST_151	9,213,824	8,365,247	90,8	326,74	3,5	7,774,494	84,4	1,439,330	15,6	8,691,987	94
SB_FST_359	12,382,726	11,302,300	91,3	431,845	3,5	10,457,104	84,4	1,925,622	15,6	11,734,145	92
LB_FST_19	8,722,979	7,958,331	91,2	302,829	3,5	7,380,285	84,6	1,342,694	15,4	8,261,160	93
LB_FST_112	10,562,263	9,658,009	91,4	365,727	3,5	8,841,507	83,7	1,720,756	16,3	10,023,736	93
LB_FST_117	9,247,029	8,447,701	91,4	320,522	3,5	7,707,007	83,3	1,540,022	16,7	8,768,223	93
Sul_FST	11,660,302	10,623,506	91,1	410,824	3,5	9,827,322	84,3	1,832,980	15,7	11,034,330	95
Ruby_FST	10,246,911	9,299,556	90,8	370,928	3,6	8,607,645	84,0	1,639,266	16,0	9,670,484	93
SB_B68_91	5,461,042	4,994,393	91,5	186,747	4,0	4,617,289	84,5	843,753	15,5	5,181,140	94
SB_B68_359	4,559,028	4,174,556	91,6	154,242	4,0	3,811,295	83,6	747,733	16,4	4,328,798	92
LB_B68_19	11,412,160	10,383,355	91,0	404,375	4,0	9,582,095	84,0	1,830,065	16,0	10,787,730	95
LB_B68_112	23,401,773	21,283,048	90,9	880,988	4,0	19,488,766	83,3	3,913,007	16,7	22,164,036	95
LB_B68_117	18,384,508	16,836,870	91,6	620,645	4,0	15,359,606	83,5	3,024,902	16,5	17,457,515	95
Sul_B68	9,506,565	8,582,489	90,3	368,848	4,0	7,966,112	83,8	1,540,453	16,2	8,951,337	94

FST= Fruit set stage; B68= Berry of 6-8 mm stage.

SB= Small berry segregant; LB= Large berry segregant.
