# Supplementary material for: Transcriptome profiling of grapevine seedless segregants during berry development reveals candidate genes associated with berry weight
Source: BMC Plant Biol. 2016 Apr 26;16:104. doi: 10.1186/s12870-016-0789-1 (PMC4845426; doi:10.1186/s12870-016-0789-1)
Supplement: Additional file 4: Table S4. — Read mapping distribution summary. (PDF 49 kb) [file 12870_2016_789_MOESM4_ESM.pdf]

Table S4. Read mapping distribution summary.

Library	Total alignments bases	Exon bases (%)	UTR bases (%)	Intron bases (%)	Intergenic bases (%)	Usable bases EXON + UTR (%)
SB_FST_91	7,495,375	63	18	9	8	82
SB_FST_151	8,925,591	64	17	9	8	81
SB_FST_359	12,062,629	65	17	9	8	82
SB_FST_19	8,494,918	63	17	9	9	80
LB_FST_112	10,321,737	63	19	8	8	83
LB_FST_117	9,029,930	68	15	8	7	84
Sul_FST	9,940,762	64	17	9	8	82
Ruby_FST	11,352,988	65	17	9	8	82
SB_B68_91	5,336,561	63	18	9	8	82
SB__B68_359	4,458,309	65	17	9	7	83
LB_B68_19	11,093,634	65	17	9	7	83
LB_B68_112	22,199,002	69	16	8	6	85
LB_B68_117	17,945,407	65	18	8	7	83
Sul_B68	9,192,449	66	17	8	7	83

FST= Fruit set stage; B68= Berry of 6-8 mm stage.

SB= Small berry segregant; LB= Large berry segregant.
