# Supplementary material for: Transcriptome profiling of grapevine seedless segregants during berry development reveals candidate genes associated with berry weight
Source: BMC Plant Biol. 2016 Apr 26;16:104. doi: 10.1186/s12870-016-0789-1 (PMC4845426; doi:10.1186/s12870-016-0789-1)
Supplement: Additional file 5: Table S5. — Unmapped reads summary describing the total of contigs mapped to the reference genome using MegaBlast program. (PDF 57 kb) [file 12870_2016_789_MOESM5_ESM.pdf]

Table S5. Unmapped reads summary describing the total of *de novo* contigs mapped to the reference genome using MegaBlast program.

Library	Total unmapped reads	Total of *de novo* contigs	Minimal contigs length	Maximal contigs length	Average contigs length	Contigs match to reference
SB_FST_91	428,319	134	302	10,772	741	19
SB_FST_151	488,608	201	302	10,795	682	46
SB_FST_359	1,006,267	259	302	10,772	629	32
LB_FST_19	560,995	146	306	10,772	687	27
LB_FST_112	705,145	293	301	10,772	618	37
LB_FST_117	590,731	268	301	13,292	629	19
Sul_FST	635,651	155	303	10,772	715	32
Ruby_FST	547,595	194	301	10,772	657	38
SB_B68_91	317,723	97	301	2,790	675	13
SB_B68_359	354,010	90	302	4,426	626	15
LB_B68_19	503,741	170	301	10,772	672	33
LG_B68_112	1,099,044	227	301	6,395	660	55
LB_B68_117	837,560	240	301	10,772	705	62
Sul_B68	489,404	151	302	10,772	726	29

FST= Fruit setting stage; B68= Berry of 6-8 mm stage.

SB= Small berry segregant; LB= Large berry segregant.
