# Supplementary material for: Transcriptome profiling of grapevine seedless segregants during berry development reveals candidate genes associated with berry weight
Source: BMC Plant Biol. 2016 Apr 26;16:104. doi: 10.1186/s12870-016-0789-1 (PMC4845426; doi:10.1186/s12870-016-0789-1)
Supplement: Additional file 6: Table S6. — Differentially expressed (DE) genes identified in the comparison between LB and SB segregants, in the FST stage (Cuffdiff2, p < 0.01). (PDF 117 kb) [file 12870_2016_789_MOESM6_ESM.pdf]

**Table S6. Differentially expressed (DE) genes identified in the comparison between LB and SB segregants, in the FST stage (Cuffdiff2, p < 0.01).**

| Gene_ID | Chrom | $log_2$ (FC) | p_value | Description |
|---|---|---|---|---|
| GSVIVG01024301001 | 16 | 4.12 | 0.00 | Ca2+-dependent lipid-binding protein CLB1/vesicle protein vp115/Granuphilin A contains C2 domain |
| GSVIVG01016069001 | 9 | 4.05 | 0.00 | Protein WAX2 |
| GSVIVG01015141001 | 11 | 3.91 | 0.01 | Ankyrin repeat-containing protein At3g12360 |
| GSVIVG01011486001 | 14 | 3.56 | 0.01 | Phosphatidylinositol-345-trisphosphate 3-phosphatase and dual-specificity protein phosphatase PTEN |
| GSVIVG01022448001 | 8 | 3.25 | 0.00 | F-box/LRR-repeat protein At3g48880 |
| GSVIVG01023110001 | 12 | 3.23 | 0.00 | Glutathione S-transferase zeta class |
| GSVIVG01029491001 | 9 | 3.13 | 0.01 | Lupeol synthase 5 |
| GSVIVG01031484001 | 6 | 3.13 | 0.00 | Vacuolar amino acid transporter 1 |
| GSVIVG01029641001 | 9 | 2.74 | 0.00 | 60S ribosomal protein L7-3 |
| GSVIVG01015194001 | 11 | 2.71 | 0.00 | Cytokinin dehydrogenase 3 |
| GSVIVG01011406001 | 14 | 2.60 | 0.00 | Vacuolar amino acid transporter 1 |
| GSVIVG01018891001 | 4 | 2.58 | 0.00 | Abscisic acid 8'-hydroxylase 3 |
| GSVIVG01012020001 | 1 | 2.43 | 0.01 | Probable E3 ubiquitin-protein ligase RHA2B |
| GSVIVG01007745001 | 17 | 2.43 | 0.00 | Carbonic anhydrase chloroplastic |
| GSVIVG01021134001 | 10 | 2.28 | 0.00 | Isoflavone-7-O-methyltransferase 9 |
| GSVIVG01026803001 | 15 | 2.23 | 0.01 | Isoflavone-7-O-methyltransferase 6 |
| GSVIVG01029525001 | 9 | 2.20 | 0.00 | Camelliol C synthase |
| GSVIVG01029631001 | 9 | 2.17 | 0.00 | 60S ribosomal protein L7-4 |
| GSVIVG01019570001 | 2 | 2.13 | 0.01 | Cellulose synthase-like protein G3 |
| GSVIVG01027716001 | 5 | 2.04 | 0.01 | Cellulose synthase-like protein G2 |
| GSVIVG01011401001 | 14 | 1.99 | 0.00 | Vacuolar amino acid transporter 1 |
| GSVIVG01024424001 | 16 | 1.98 | 0.01 | LRR receptor-like serine/threonine-protein kinase ERECTA |
| GSVIVG01036853001 | 2 | 1.97 | 0.00 | Cysteine-rich receptor-like protein kinase 10 |

| | | | | |
|---|---|---|---|---|
| GSVIVG01019490001 | 2 | 1.92 | 0.01 | Unkown Protein Function |
| GSVIVG01030219001 | 8 | 1.86 | 0.00 | Cationic peroxidase 1 |
| GSVIVG01024885001 | 6 | 1.85 | 0.00 | Tropinone reductase homolog At1g07440 |
| GSVIVG01013763001 | 1 | 1.77 | 0.00 | Glycosyl transferase family 8 - glycogenin |
| GSVIVG01035315001 | 4 | 1.74 | 0.00 | Probable leucine-rich repeat receptor-like protein kinase At5g61480 |
| GSVIVG01004877001 | Un | 1.74 | 0.01 | TMV resistance protein N |
| GSVIVG01024892001 | 6 | 1.71 | 0.00 | Tropinone reductase homolog At1g07440 |
| GSVIVG01032425001 | 14 | 1.67 | 0.00 | Galactinol--sucrose galactosyltransferase |
| GSVIVG01022171001 | 7 | 1.66 | 0.01 | Unkown Protein Function |
| GSVIVG01014559001 | 19 | 1.63 | 0.01 | Vacuolar H+-ATPase V0 sector subunit c" |
| GSVIVG01019452001 | 2 | 1.62 | 0.01 | Unkown Protein Function |
| GSVIVG01010719001 | 13 | 1.51 | 0.01 | Unkown Protein Function |
| GSVIVG01024879001 | 6 | 1.37 | 0.01 | Tropinone reductase homolog At1g07440 |
| GSVIVG01036279001 | 14 | 1.32 | 0.01 | Pathogenesis-related protein PR-4B |
| GSVIVG01027600001 | 15 | 1.28 | 0.01 | Dihydroflavonol-4-reductase |
| GSVIVG01037055001 | 3 | 1.25 | 0.01 | Unkown Protein Function |
| GSVIVG01022913001 | 12 | -1.40 | 0.00 | nutrient reservoir putative |
| GSVIVG01021609001 | 10 | -1.41 | 0.00 | Uncharacterized amino acid permease yfnA |
| GSVIVG01035059001 | 5 | -1.49 | 0.00 | Major allergen Pru av 1 |
| GSVIVG01012363001 | 13_random | -1.52 | 0.01 | Myrcene synthase chloroplastic |
| GSVIVG01000403001 | 12 | -1.53 | 0.01 | Isoprene synthase chloroplastic |
| GSVIVG01027790001 | 5 | -1.61 | 0.01 | Unkown Protein Function |
| GSVIVG01008910001 | 18 | -1.66 | 0.00 | Unkown Protein Function |
| GSVIVG01021724001 | 10 | -1.79 | 0.01 | Unkown Protein Function |
| GSVIVG01033484001 | 8 | -1.92 | 0.00 | Peroxidase 53 |
| GSVIVG01015071001 | 11 | -2.00 | 0.00 | Cytochrome P450 71A1 |
| GSVIVG01037077001 | 3 | -2.06 | 0.00 | Eugenol synthase 1 |
| GSVIVG01015076001 | 11 | -2.07 | 0.01 | Flavonoid 3'5'-hydroxylase 1 |
| GSVIVG01027568001 | 15 | -2.07 | 0.01 | Gibberellin receptor GID1 |
| GSVIVG01037073001 | 3 | -2.08 | 0.00 | Eugenol synthase 1 |
| GSVIVG01000567001 | 1 | -2.13 | 0.00 | Cytochrome P450 71B35 |
| GSVIVG01021278001 | 10 | -2.15 | 0.01 | Probable LRR receptor-like serine/threonine-protein kinase At1g53430 |
| GSVIVG01036885001 | 2 | -2.17 | 0.00 | Abscisic acid 8'-hydroxylase 1 |

| | | | | |
|---|---|---|---|---|
| GSVIVG01024292001 | 16 | -2.17 | 0.00 | Phenylalanine ammonia-lyase |
| GSVIVG01027558001 | 15 | -2.30 | 0.00 | Cytochrome P450 76C2 |
| GSVIVG01026810001 | 15 | -2.37 | 0.00 | Protein WAX2 |
| GSVIVG01025391001 | 6 | -2.37 | 0.01 | Unkown Protein Function |
| GSVIVG01027785001 | 5 | -2.41 | 0.00 | Unkown Protein Function |
| GSVIVG01018990001 | 4 | -2.43 | 0.00 | Flavin-containing monooxygenase YUCCA6 |
| GSVIVG01032091001 | 13 | -2.48 | 0.01 | Uncharacterized membrane protein predicted efflux pump |
| GSVIVG01018142001 | 5 | -2.55 | 0.00 | PR6 protease inhibitor |
| GSVIVG01010565001 | 16 | -2.57 | 0.00 | Stilbene synthase 2 |
| GSVIVG01025394001 | 6 | -2.62 | 0.00 | Unkown Protein Function |
| GSVIVG01010561001 | 16 | -2.73 | 0.00 | Stilbene synthase 1 |
| GSVIVG01035076001 | 5 | -2.78 | 0.00 | Pathogenesis-related protein STH-2 |
| GSVIVG01036322001 | 18_random | -2.79 | 0.00 | (+)-delta-cadinene synthase isozyme A |
| GSVIVG01026220001 | 10 | -2.89 | 0.00 | Stilbene synthase 3 |
| GSVIVG01025284001 | 6 | -3.00 | 0.00 | Unkown Protein Function |
| GSVIVG01010580001 | 16 | -3.04 | 0.00 | Stilbene synthase 2 |
| GSVIVG01010591001 | 16 | -3.07 | 0.00 | Unkown Protein Function |
| GSVIVG01010584001 | 16 | -3.12 | 0.00 | Stilbene synthase 4 |
| GSVIVG01029110001 | 11 | -3.14 | 0.00 | Proteasome subunit alpha type-2-B |
| GSVIVG01010583001 | 16 | -3.16 | 0.00 | Stilbene synthase 4 |
| GSVIVG01038414001 | 9 | -3.17 | 0.00 | Omega-hydroxypalmitate O-feruloyl transferase |
| GSVIVG01010590001 | 16 | -3.18 | 0.00 | Stilbene synthase 3 |
| GSVIVG01024305001 | 16 | -3.20 | 0.01 | Phenylalanine ammonia-lyase 2 |
| GSVIVG01032256001 | 11 | -3.20 | 0.00 | Trans-cinnamate 4-monooxygenase |
| GSVIVG01024295001 | 16 | -3.20 | 0.01 | Phenylalanine ammonia-lyase 2 |
| GSVIVG01010579001 | 16 | -3.22 | 0.00 | Stilbene synthase 4 |
| GSVIVG01025287001 | 6 | -3.28 | 0.00 | Unkown Protein Function |
| GSVIVG01024294001 | 16 | -3.31 | 0.00 | Phenylalanine ammonia-lyase |
| GSVIVG01010578001 | 16 | -3.34 | 0.00 | Stilbene synthase 4 |
| GSVIVG01024315001 | 16 | -3.37 | 0.01 | Phenylalanine ammonia-lyase |
| GSVIVG01010589001 | 16 | -3.45 | 0.00 | Stilbene synthase 3 |
| GSVIVG01024293001 | 16 | -3.49 | 0.01 | Phenylalanine ammonia-lyase G2B |
| GSVIVG01024306001 | 16 | -3.64 | 0.00 | Phenylalanine ammonia-lyase |
| GSVIVG01010556001 | 16 | -3.72 | 0.00 | Stilbene synthase 1 |
| GSVIVG01010582001 | 16 | -3.88 | 0.00 | Stilbene synthase 3 |
| GSVIVG01024299001 | 16 | -3.89 | 0.00 | Phenylalanine ammonia-lyase 2 |

| | | | | |
|---|---|---|---|---|
| GSVIVG01024756001 | 6 | -3.96 | 0.00 | 4-hydroxyphenylacetaldehyde oxime monooxygenase |
| GSVIVG01010585001 | 16 | -3.99 | 0.00 | Stilbene synthase 4 |
| GSVIVG01010568001 | 16 | -4.17 | 0.00 | Stilbene synthase 6 |
| GSVIVG01028606001 | 7 | -4.41 | 0.00 | Unkown Protein Function |
| GSVIVG01010581001 | 16 | -4.48 | 0.00 | Stilbene synthase 4 |
| GSVIVG01028618001 | 7 | -4.61 | 0.00 | Pore-forming toxin-like protein Hfr-2 |
| GSVIVG01026213001 | 10 | -4.79 | 0.00 | Stilbene synthase 1 |
| GSVIVG01010557001 | 16 | -4.90 | 0.00 | Stilbene synthase 4 |
| GSVIVG01031746001 | 3 | -6.32 | 0.00 | Alpha-amylase |

Gene_ID= Gene code nomenclature based on reference genome annotation PN40024 (12X.v1); $\log_2$(FC) = log base 2 of fold change; p_value = differential expression significance.
