# Supplementary material for: Transcriptome profiling of grapevine seedless segregants during berry development reveals candidate genes associated with berry weight
Source: BMC Plant Biol. 2016 Apr 26;16:104. doi: 10.1186/s12870-016-0789-1 (PMC4845426; doi:10.1186/s12870-016-0789-1)
Supplement: Additional file 7: Table S7. — Differentially expressed (DE) genes identified in the comparison between LB and SB segregants, in the B68 stage (Cuffdiff2, p < 0.01). (PDF 327 kb) [file 12870_2016_789_MOESM7_ESM.pdf]

Table S7. Differentially expressed (DE) genes identified in the comparison between LB and SB segregants, in the B68 stage (Cuffdiff2, p < 0.01).

Gene_ID	Chrom	log_2 (FC)	p_value	Description
GSVIVG01003747001	11	1.79769 e+308	0.01	Unkown Protein Function
GSVIVG01009810001	18	1.79769 e+308	0.01	Cysteine proteinase RD19a
GSVIVG01017084001	9	1.79769 e+308	0.01	Putative peroxidase 48
GSVIVG01035433001	4	5.45	0.00	17.9 kDa class II heat shock protein
GSVIVG01021308001	10	5.44	0.00	GDSL esterase/lipase At1g29670
GSVIVG01035434001	4	4.97	0.00	17.9 kDa class II heat shock protein
GSVIVG01010631001	16	4.53	0.01	Dehydration-responsive element-binding protein 1A
GSVIVG01019407001	2	4.36	0.00	Heat shock protein 83
GSVIVG01001315001	2	3.99	0.00	Small heat shock protein chloroplastic
GSVIVG01029491001	9	3.93	0.00	Lupeol synthase 5
GSVIVG01030360001	12	3.85	0.00	Ankyrin-1
GSVIVG01030362001	12	3.65	0.00	Serine/threonine-protein phosphatase 6 regulatory ankyrin repeat subunit B
GSVIVG01031747001	3	3.60	0.00	Alpha-amylase type B isozyme
GSVIVG01018654001	16	3.56	0.00	23.6 kDa heat shock protein mitochondrial
GSVIVG01028856001	16	3.49	0.00	Heat shock cognate protein 80
GSVIVG01024301001	16	3.45	0.00	Ca2+-dependent lipid-binding protein CLB1/vesicle protein vp115/Granuphilin A contains C2 domain
GSVIVG01015485001	11	3.45	0.00	Secologanin synthase
GSVIVG01013045001	2	3.41	0.00	Cytochrome P450 76C4
GSVIVG01035385001	4	3.30	0.00	Heat shock factor protein HSF30
GSVIVG01016428001	13	3.30	0.00	17.3 kDa class I heat shock protein
GSVIVG01020329001	19	3.29	0.01	Polygalacturonase
GSVIVG01035430001	4	3.27	0.00	17.3 kDa class II heat shock protein

GSVIVG01008401001	17	3.26	0.00	Transcription factor MYB3
GSVIVG01016856001	9	3.25	0.01	Protein HOTHEAD
GSVIVG01016403001	13	3.21	0.00	17.6 kDa class I heat shock protein 3
GSVIVG01035435001	4	3.20	0.00	17.9 kDa class II heat shock protein
GSVIVG01016426001	13	3.20	0.00	Unkown Protein Function
GSVIVG01003118001	Un	3.18	0.00	Heat stress transcription factor A-2b
GSVIVG01003253001	Un	3.15	0.00	DNA replication licensing factor mcm6
GSVIVG01010308001	1	3.15	0.00	25.3 kDa heat shock protein chloroplastic
GSVIVG01032703001	13	3.14	0.00	UPF0557 protein C10orf119 homolog
GSVIVG01035428001	4	3.13	0.01	17.9 kDa class II heat shock protein
GSVIVG01016429001	13	3.11	0.00	18.2 kDa class I heat shock protein
GSVIVG01028522001	7	3.09	0.00	Unkown Protein Function
GSVIVG01021415001	10	3.06	0.01	Mitochondrial inner membrane protease subunit 1
GSVIVG01035436001	4	3.06	0.00	17.9 kDa class II heat shock protein
GSVIVG01016697001	9	3.01	0.00	18.6 kDa class III heat shock protein
GSVIVG01017233001	9	3.00	0.00	Unkown Protein Function
GSVIVG01020682001	12	2.98	0.00	Os01g0614300
GSVIVG01023641001	11	2.98	0.00	Heat shock 70 kDa protein
GSVIVG01028174001	7	2.97	0.00	Glycogenin-1
GSVIVG01035431001	4	2.97	0.01	Unkown Protein Function
GSVIVG01019816001	2	2.94	0.00	Predicted regulator of the ubiquitin pathway (contains UAS and UBX domains)
GSVIVG01024054001	3	2.92	0.00	Auxin transporter-like protein 3
GSVIVG01033633001	8	2.90	0.01	Flavonoid 3'5'-hydroxylase
GSVIVG01027380001	13	2.89	0.00	HSP90 co-chaperone putative
GSVIVG01008158001	17	2.88	0.01	DNA polymerase epsilon subunit 2
GSVIVG01025945001	18	2.87	0.00	Agamous-like MADS-box protein AGL11
GSVIVG01005154001	Un	2.87	0.00	Predicted histone tail methylase containing SET domain
GSVIVG01008469001	17	2.83	0.00	Nudix hydrolase 17 mitochondrial
GSVIVG01024572001	6	2.83	0.00	Expansin-A8

GSVIVG01007880001	17	2.79	0.00	Chaperone protein ClpB 1
GSVIVG01001298001	2	2.77	0.00	36.4 kDa proline-rich protein
GSVIVG01001046001	11	2.76	0.00	DNA replication licensing factor MCM5
GSVIVG01000023001	14	2.71	0.00	Unkown Protein Function
GSVIVG01024207001	3	2.67	0.00	DNA replication licensing factor mcm4
GSVIVG01035964001	4	2.64	0.01	Centromere-associated protein NUF2
GSVIVG01003320001	Un	2.63	0.00	Cysteine proteinase inhibitor 1
GSVIVG01016572001	13	2.61	0.01	15.7 kDa heat shock protein peroxisomal
GSVIVG01024725001	6	2.61	0.00	Omega-3 fatty acid desaturase chloroplastic
GSVIVG01036543001	14	2.57	0.01	Pollen Ole e 1 allergen and extensin family protein
GSVIVG01029158001	11	2.43	0.00	Probable xyloglucan endotransglucosylase/hydrolase protein 23
GSVIVG01008850001	18	2.43	0.00	Two-component response regulator ARR9
GSVIVG01022113001	7	2.41	0.00	DNA polymerase alpha catalytic subunit
GSVIVG01021112001	10	2.40	0.00	DnaJ homolog subfamily B member 13
GSVIVG01035860001	4	2.36	0.00	Unkown Protein Function
GSVIVG01021426001	10	2.32	0.00	Maternal DNA replication licensing factor mcm3
GSVIVG01028176001	7	2.31	0.01	Glycogenin-1
GSVIVG01013467001	18	2.30	0.00	Calcium-binding protein CML37
GSVIVG01003386001	Un	2.30	0.00	Unkown Protein Function
GSVIVG01034649001	13	2.30	0.00	Unkown Protein Function
GSVIVG01032681001	13	2.30	0.00	Expansin-A8
GSVIVG01021978001	14	2.30	0.00	Bifunctional 3-dehydroquinate dehydratase/shikimate dehydrogenase chloroplastic
GSVIVG01000981001	13	2.30	0.01	Unkown Protein Function
GSVIVG01011760001	1	2.30	0.00	DnaJ homolog subfamily B member 8
GSVIVG01029411001	17	2.29	0.00	Expansin-A15
GSVIVG01011742001	1	2.27	0.00	10 kDa chaperonin
GSVIVG01026974001	15	2.27	0.01	Late embryogenesis abundant protein Lea14-A
GSVIVG01011715001	1	2.26	0.00	Putative phosphoethanolamine N-methyltransferase 2

GSVIVG01000923001	1	2.25	0.01	Bcl-2-associated athanogene-like protein
GSVIVG01007942001	17	2.25	0.01	Unkown Protein Function
GSVIVG01001119001	1	2.24	0.00	DNA replication licensing factor MCM2
GSVIVG01021406001	10	2.22	0.00	Chlorophyll a-b binding protein type 2 member 1B chloroplastic
GSVIVG01012069001	1	2.21	0.01	Unkown Protein Function
GSVIVG01034174001	8	2.20	0.00	Metallothionein-like protein type 2
GSVIVG01017946001	5	2.20	0.01	Uncharacterized protein C24B11.05
GSVIVG01024994001	6	2.20	0.00	Heat shock cognate 70 kDa protein 2
GSVIVG01017806001	5	2.18	0.01	Unkown Protein Function
GSVIVG01003904001	Un	2.17	0.00	F-box protein PP2-B10
GSVIVG01000021001	14	2.17	0.00	Copper chaperone
GSVIVG01010781001	5	2.14	0.00	Unkown Protein Function
GSVIVG01033414001	8	2.14	0.00	Putative mitochondrial 2-oxoglutarate/malate carrier protein
GSVIVG01026014001	18	2.12	0.00	Chaperone protein dnaK2
GSVIVG01016413001	13	2.09	0.00	17.5 kDa class I heat shock protein
GSVIVG01018447001	16	2.05	0.01	Unkown Protein Function
GSVIVG01029508001	9	2.01	0.00	Lupeol synthase 5
GSVIVG01029311001	11	1.99	0.00	Transcription factor bHLH35
GSVIVG01016396001	13	1.99	0.00	17.5 kDa class I heat shock protein
GSVIVG01024694001	6	1.98	0.00	GCN5-related N-acetyltransferase (GNAT) family protein
GSVIVG01003204001	Un	1.98	0.00	Chlorophyll a-b binding protein 13 chloroplastic
GSVIVG01035900001	4	1.97	0.00	Chaperone protein ClpB 2
GSVIVG01000461001	12	1.96	0.00	Anthocyanin 5-aromatic acyltransferase
GSVIVG01005193001	Un	1.94	0.00	Unkown Protein Function
GSVIVG01024077001	3	1.93	0.00	Unkown Protein Function
GSVIVG01037142001	18	1.92	0.00	RING-H2 finger protein ATL7
GSVIVG01000469001	12	1.90	0.00	Anthocyanin 5-aromatic acyltransferase
GSVIVG01033350001	8	1.90	0.00	Transcription factor bHLH60
GSVIVG01024815001	6	1.89	0.00	Proliferating cell nuclear antigen
GSVIVG01005149001	Un	1.85	0.00	70 kDa peptidyl-prolyl

				isomerase
GSVIVG01027182001	15	1.84	0.00	Myb-related protein Zm38
GSVIVG01000468001	12	1.83	0.01	Anthocyanin 5-aromatic acyltransferase
GSVIVG01009152001	18	1.82	0.00	Probable mitochondrial 2-oxoglutarate/malate carrier protein
GSVIVG01009363001	18	1.82	0.00	Unkown Protein Function
GSVIVG01035110001	5	1.81	0.01	Unkown Protein Function
GSVIVG01023115001	12	1.77	0.00	gb|AAW38977.1| At5g49800
GSVIVG01003201001	Un	1.77	0.00	Chlorophyll a-b binding protein 13 chloroplastic
GSVIVG01018781001	4	1.74	0.00	Naringenin2-oxoglutarate 3-dioxygenase
GSVIVG01015354001	11	1.73	0.01	Bark storage protein A
GSVIVG01011412001	14	1.73	0.00	Gibberellin-regulated protein 4
GSVIVG01021289001	10	1.72	0.00	Probable LRR receptor-like serine/threonine-protein kinase RFK1
GSVIVG01009083001	18	1.72	0.00	Homeobox-leucine zipper protein ATHB-16
GSVIVG01013917001	16	1.71	0.00	Ethylene-responsive transcription factor 5
GSVIVG01033476001	8	1.71	0.00	Activator of 90 kDa heat shock protein ATPase homolog
GSVIVG01017960001	5	1.71	0.01	Heat shock cognate 70 kDa protein 4
GSVIVG01028042001	7	1.70	0.01	Endoglucanase 1
GSVIVG01026803001	15	1.70	0.00	Isoflavone-7-O-methyltransferase 6
GSVIVG01037052001	3	1.66	0.00	EGY3 EGY3 (ETHYLENE-DEPENDENT GRAVITROPISM-DEFICIENT AND YELLOW-GREEN-LIKE 3)
GSVIVG01022680001	2	1.65	0.00	Protease Ulp1 family
GSVIVG01001036001	11	1.65	0.00	Sugar carrier protein A
GSVIVG01021237001	10	1.64	0.01	Nucleobase-ascorbate transporter 1
GSVIVG01016417001	13	1.62	0.00	Cytokinin-O-glucosyltransferase 2
GSVIVG01002899001	Un	1.62	0.00	70 kDa peptidyl-prolyl isomerase
GSVIVG01036671001	13	1.61	0.00	Aspartic proteinase nepenthesin-1

GSVIVG01026491001	4	1.60	0.00	Transcriptional corepressor SEUSS
GSVIVG01037059001	3	1.59	0.00	Serine carboxypeptidase-like 18
GSVIVG01009624001	18	1.58	0.01	Oxygen-evolving enhancer protein 1 chloroplastic
GSVIVG01020806001	12	1.58	0.00	Protein TRANSPARENT TESTA 12
GSVIVG01002527001	Un	1.57	0.00	70 kDa peptidyl-prolyl isomerase
GSVIVG01009155001	18	1.56	0.00	Aspartic proteinase nepenthesin-1
GSVIVG01029789001	12	1.54	0.00	Chlorophyll a-b binding protein CP24 10A chloroplastic
GSVIVG01030503001	12	1.54	0.00	Glucan endo-13-beta-glucosidase 3
GSVIVG01034195001	8	1.50	0.00	Heat shock cognate 70 kDa protein 2
GSVIVG01007988001	17	1.49	0.00	Chlorophyll a-b binding protein 4 chloroplastic
GSVIVG01020808001	12	1.49	0.01	Protein TRANSPARENT TESTA 12
GSVIVG01030056001	12	1.48	0.00	Heat shock cognate protein 80
GSVIVG01037892001	3	1.46	0.00	Probable indole-3-acetic acid-amido synthetase GH3.1
GSVIVG01008851001	18	1.46	0.00	Delta-aminolevulinic acid dehydratase chloroplastic
GSVIVG01017158001	9	1.46	0.00	Auxin-induced protein AUX22
GSVIVG01024710001	6	1.45	0.01	Probable protein phosphatase 2C 25
GSVIVG01034194001	8	1.45	0.00	Unkown Protein Function
GSVIVG01016857001	9	1.45	0.00	Serine/threonine-protein kinase PBS1
GSVIVG01023783001	3	1.43	0.01	Transcription factor bHLH63
GSVIVG01030386001	12	1.42	0.01	Protochlorophyllide reductase chloroplastic
GSVIVG01025088001	6	1.41	0.00	Two-component response regulator-like PRR73
GSVIVG01018556001	16	1.41	0.00	EXL3 EXL3 (EXORDIUM LIKE 3)
GSVIVG01019052001	4	1.40	0.00	Endoribonuclease Dicer homolog 2b
GSVIVG01031471001	6	1.37	0.01	Unkown Protein Function
GSVIVG01014388001	19	1.37	0.00	Protochlorophyllide reductase chloroplastic
GSVIVG01000669001	13	1.36	0.00	Unkown Protein Function
GSVIVG01019892001	2	1.35	0.00	Leucoanthocyanidin

				dioxygenase
GSVIVG01016053001	9	1.35	0.00	Anthranilate N-benzoyltransferase protein 2
GSVIVG01029025001	5	1.34	0.00	Chaperonin CPN60-1 mitochondrial
GSVIVG01017062001	9	1.31	0.01	gb|AAO63445.1| At2g25737
GSVIVG01024805001	6	1.30	0.01	Uncharacterized RNA-binding protein C25G10.01
GSVIVG01000114001	7	1.24	0.01	60S ribosomal protein L26-2
GSVIVG01016700001	9	1.22	0.01	Chaperone protein dnaJ
GSVIVG01036167001	6	1.22	0.01	Cucumisin
GSVIVG01007878001	17	1.22	0.01	Flavonoid 3'-monooxygenase
GSVIVG01004715001	Un	1.20	0.01	DUF246 domain-containing protein At1g04910
GSVIVG01024050001	3	1.16	0.01	Pathogenesis-related protein 5
GSVIVG01009743001	18	1.15	0.01	Dihydroflavonol-4-reductase
GSVIVG01029048001	5	1.11	0.01	Probable pectate lyase 10
GSVIVG01033076001	14	-1.06	0.01	nodulin family protein
GSVIVG01038001001	10	-1.09	0.01	Unkown Protein Function
GSVIVG01036470001	14	-1.11	0.01	Vacuolar cation/proton exchanger 2
GSVIVG01001340001	1_random	-1.12	0.01	Salt stress root protein RS1
GSVIVG01027015001	15	-1.12	0.01	Fatty acid desaturase 3
GSVIVG01024116001	3	-1.13	0.01	calcium ion binding protein putative
GSVIVG01024596001	6	-1.14	0.01	Peroxidase 4
GSVIVG01008065001	17	-1.15	0.00	Homeobox-leucine zipper protein HOX16
GSVIVG01011500001	14	-1.15	0.01	Probable galacturonosyltransferase 13
GSVIVG01023803001	3	-1.15	0.01	F-box protein At2g16365
GSVIVG01006876001	Un	-1.15	0.01	Aspartic proteinase nepenthesin-1
GSVIVG01011711001	1	-1.16	0.01	FOG: PPR repeat
GSVIVG01031320001	14	-1.16	0.01	Nucleic acid binding protein putative
GSVIVG01016972001	9	-1.16	0.01	Auxin-responsive protein IAA26
GSVIVG01021128001	10	-1.17	0.00	Auxin response factor 3
GSVIVG01033935001	8	-1.17	0.00	Aspartic proteinase nepenthesin-2
GSVIVG01028213001	7	-1.18	0.01	Nucleic acid binding protein putative
GSVIVG01030501001	12	-1.18	0.01	Unkown Protein Function

GSVIVG01038647001	16	-1.18	0.01	Two-component response regulator-like APRR5
GSVIVG01013279001	2	-1.19	0.01	Phosphatidylinositol-4-phosphate 5-kinase 5
GSVIVG01037572001	6	-1.19	0.00	Uncharacterized basic helix-loop-helix protein At1g64625
GSVIVG01033540001	8	-1.19	0.01	Glucan endo-13-beta-glucosidase
GSVIVG01007961001	17	-1.20	0.01	LON peptidase N-terminal domain and RING finger protein 1
GSVIVG01034151001	8	-1.21	0.01	Uncharacterized protein C24B11.05
GSVIVG01009475001	18	-1.21	0.01	Unkown Protein Function
GSVIVG01033956001	8	-1.21	0.00	Probable receptor protein kinase TMK1
GSVIVG01014705001	19	-1.23	0.00	Cellulose synthase-like protein E1
GSVIVG01021507001	10	-1.23	0.01	Probable 9-cis-epoxycarotenoid dioxygenase NCED5 chloroplastic
GSVIVG01011480001	14	-1.24	0.01	Hypothetical protein LOC100261075 putative transcriptional regulator
GSVIVG01036583001	13	-1.24	0.01	Probable cytochrome P450 313a3
GSVIVG01009851001	18	-1.24	0.01	Cytochrome P450 83B1
GSVIVG01022014001	14	-1.25	0.00	Probable gibberellin receptor GID1L1
GSVIVG01031678001	5	-1.26	0.01	Cytokinin-O-glucosyltransferase 1
GSVIVG01024773001	6	-1.26	0.01	Similar to binding
GSVIVG01015882001	3	-1.26	0.00	Zinc transporter 8
GSVIVG01019722001	2	-1.27	0.00	Transcription factor GT-2 and related proteins contains trihelix DNA-binding/SANT domain
GSVIVG01016999001	9	-1.27	0.00	Pleiotropic drug resistance protein 15
GSVIVG01023336001	12	-1.29	0.01	Probable mitochondrial chaperone bcs1
GSVIVG01008427001	17	-1.29	0.01	Integral membrane protein DUF6 containing protein
GSVIVG01018000001	5	-1.30	0.00	Unkown Protein Function
GSVIVG01013237001	2	-1.31	0.00	Type I inositol-145-trisphosphate 5-phosphatase 2
GSVIVG01022185001	7	-1.31	0.01	Copper chaperone

GSVIVG01037782001	19	-1.31	0.00	Zinc finger CCCH domain-containing protein 18
GSVIVG01023327001	12	-1.31	0.01	Unkown Protein Function
GSVIVG01015991001	9	-1.31	0.00	Chaperone protein dnaJ 8 chloroplastic
GSVIVG01018648001	16	-1.32	0.00	Sugar phosphate exchanger 2
GSVIVG01003431001	7	-1.32	0.00	Homeobox-leucine zipper protein ATHB-6
GSVIVG01026876001	15	-1.33	0.01	KTEL motif-containing protein 1
GSVIVG01037740001	19	-1.33	0.00	Unkown Protein Function
GSVIVG01014703001	19	-1.33	0.00	Cellulose synthase-like protein E1
GSVIVG01015353001	11	-1.33	0.00	Transcription factor bHLH68
GSVIVG01011673001	1	-1.34	0.01	Calcium ion binding protein putative
GSVIVG01017714001	5	-1.35	0.01	Transcription factor HY5-like
GSVIVG01009276001	18	-1.35	0.01	Branched-chain-amino-acid aminotransferase 2 chloroplastic
GSVIVG01010060001	1	-1.35	0.00	Probable GMP synthase [glutamine-hydrolyzing]
GSVIVG01008461001	17	-1.36	0.00	Uncharacterized oxidoreductase ygbJ
GSVIVG01021293001	10	-1.36	0.01	Probable LRR receptor-like serine/threonine-protein kinase At1g07650
GSVIVG01029680001	12	-1.36	0.00	Anthocyanidin 3-O-glucosyltransferase 1
GSVIVG01013544001	5	-1.38	0.01	Unkown Protein Function
GSVIVG01001853001	5_random	-1.39	0.01	Ammonium transporter 1 member 1
GSVIVG01007666001	17	-1.40	0.00	DEAD-box ATP-dependent RNA helicase 30
GSVIVG01015767001	3	-1.40	0.00	ABC transporter G family member 11
GSVIVG01009256001	18	-1.40	0.01	Receptor-like protein kinase HSL1
GSVIVG01000940001	1	-1.41	0.00	NAC domain-containing protein 29
GSVIVG01004265001	Un	-1.41	0.00	Aspartyl protease
GSVIVG01006161001	Un	-1.41	0.00	Glycogenin-2
GSVIVG01036558001	14	-1.41	0.00	Cytochrome P450 85A1
GSVIVG01008270001	17	-1.41	0.01	Predicted membrane protein
GSVIVG01023740001	11	-1.42	0.00	Protein WAX2
GSVIVG01027145001	15	-1.42	0.00	O-acyltransferase WSD1

GSVIVG01011433001	14	-1.43	0.00	Dof zinc finger protein DOF5.2
GSVIVG01021126001	10	-1.43	0.01	MLO-like protein 12
GSVIVG01030257001	8	-1.43	0.00	F-box/LRR-repeat protein 3
GSVIVG01031964001	3	-1.44	0.01	2-hydroxyacid dehydrogenase putative
GSVIVG01029349001	17	-1.45	0.00	Probable metal-nicotianamine transporter YSL7
GSVIVG01028033001	7	-1.45	0.00	Indole-3-acetic acid-induced protein ARG2
GSVIVG01014081001	19	-1.46	0.00	Mitogen-activated protein kinase 9
GSVIVG01017808001	5	-1.46	0.00	Inactive beta-amylase 9
GSVIVG01038546001	16	-1.46	0.00	Probable pectinesterase/pectinesterase inhibitor 34
GSVIVG01032702001	13	-1.46	0.01	Unkown Protein Function
GSVIVG01027443001	15	-1.47	0.00	Two-component response regulator-like APRR5
GSVIVG01014973001	19	-1.49	0.01	Probable glutathione S-transferase parC
GSVIVG01027922001	5	-1.51	0.00	3-isopropylmalate dehydratase
GSVIVG01016441001	13	-1.52	0.01	Photosystem Q(B) protein
GSVIVG01016487001	13	-1.52	0.01	Tropinone reductase homolog At1g07440
GSVIVG01019452001	2	-1.53	0.01	Unkown Protein Function
GSVIVG01007762001	17	-1.54	0.00	Serine/threonine-protein kinase HT1
GSVIVG01009680001	18	-1.55	0.00	Zinc finger protein MAGPIE
GSVIVG01037525001	6	-1.56	0.00	Probable gibberellin receptor GID1L3
GSVIVG01034463001	18	-1.56	0.01	ABC transporter G family member 25
GSVIVG01005206001	Un	-1.57	0.00	Probable ubiquitin-conjugating enzyme E2 24
GSVIVG01010157001	1	-1.57	0.00	Unkown Protein Function
GSVIVG01035231001	4	-1.58	0.00	Protein LHY
GSVIVG01003713001	7	-1.60	0.00	protein binding protein putative
GSVIVG01008135001	17	-1.60	0.00	Unkown Protein Function
GSVIVG01017239001	9	-1.60	0.01	(6-4)DNA photolyase
GSVIVG01006748001	Un	-1.60	0.00	FOG: Ankyrin repeat
GSVIVG01023708001	11	-1.62	0.00	S-adenosylmethionine decarboxylase leader
GSVIVG01030127001	12	-1.62	0.00	Zinc finger protein CONSTANS-LIKE 9
GSVIVG01003677001	Un	-1.62	0.00	Uncharacterized Cys-rich domain

GSVIVG01030508001	12	-1.63	0.00	1-aminocyclopropane-1-carboxylate oxidase 3
GSVIVG01036148001	6	-1.65	0.00	Unkown Protein Function
GSVIVG01029545001	9	-1.66	0.01	Probable mitochondrial chaperone bcs1
GSVIVG01003722001	11	-1.66	0.00	Calmodulin-binding protein
GSVIVG01007624001	17	-1.67	0.01	High-affinity nitrate transporter 3.2
GSVIVG01011801001	1	-1.68	0.00	Protein TRANSPARENT TESTA 12
GSVIVG01008595001	17	-1.68	0.00	Protein RUPTURED POLLEN GRAIN 1
GSVIVG01015538001	11	-1.68	0.00	SPX domain-containing protein 1
GSVIVG01035078001	5	-1.70	0.01	Unkown Protein Function
GSVIVG01009528001	18	-1.71	0.00	Probable gibberellin receptor GID1L1
GSVIVG01014951001	19	-1.71	0.00	Probable glutathione S-transferase parC
GSVIVG01017926001	5	-1.71	0.00	Probable LRR receptor-like serine/threonine-protein kinase At1g67720
GSVIVG01001318001	2	-1.71	0.01	Sec14 cytosolic factor
GSVIVG01025691001	8	-1.72	0.00	Auxin response factor 8
GSVIVG01037758001	19	-1.72	0.00	Pirin-like protein
GSVIVG01017089001	9	-1.73	0.00	Mitochondrial carnitine/acylcarnitine carrier protein CACL
GSVIVG01015560001	11	-1.73	0.00	Early nodulin 16 precursor putative
GSVIVG01027558001	15	-1.74	0.00	Cytochrome P450 76C2
GSVIVG01019865001	2	-1.75	0.01	Adenosine 3'-phospho 5'-phosphosulfate transporter 2
GSVIVG01014403001	19	-1.77	0.00	NAC domain-containing protein 72
GSVIVG01005878001	Un	-1.77	0.00	Trehalose-phosphate phosphatase
GSVIVG01027876001	5	-1.77	0.00	Peptide transporter PTR3-B
GSVIVG01037014001	3	-1.81	0.00	Basic form of pathogenesis-related protein 1
GSVIVG01027588001	15	-1.82	0.01	Transcription factor TCP9
GSVIVG01037700001	19	-1.83	0.00	Unkown Protein Function
GSVIVG01011949001	1	-1.83	0.01	Probable galacturonosyltransferase-like 3
GSVIVG01035013001	5	-1.84	0.01	Glucan endo-13-beta-glucosidase basic vacuolar

				isoform
GSVIVG01035051001	5	-1.85	0.00	Two-component response regulator ARR1
GSVIVG01034066001	8	-1.87	0.00	Unkown Protein Function
GSVIVG01014106001	19	-1.88	0.00	Unkown Protein Function
GSVIVG01027803001	5	-1.88	0.00	Inorganic phosphate transporter 1-4
GSVIVG01026614001	4	-1.89	0.00	Auxin-induced protein 5NG4
GSVIVG01000579001	1	-1.90	0.00	Vegetative incompatibility protein HET-E-1
GSVIVG01022916001	12	-1.92	0.01	Glutelin type-A 3
GSVIVG01038653001	16	-1.92	0.00	Caffeic acid 3-O-methyltransferase
GSVIVG01023906001	3	-1.93	0.00	Lysine histidine transporter-like 8
GSVIVG01030330001	8	-1.93	0.00	Ammonium transporter 3 member 3
GSVIVG01021724001	10	-1.95	0.00	Unkown Protein Function
GSVIVG01031255001	14	-2.00	0.00	Unkown Protein Function
GSVIVG01034034001	8	-2.00	0.00	Fructose-16-bisphosphatase chloroplastic
GSVIVG01027784001	5	-2.00	0.00	Unkown Protein Function
GSVIVG01036279001	14	-2.00	0.00	Pathogenesis-related protein PR-4B
GSVIVG01023919001	3	-2.01	0.00	Expansin-like A2
GSVIVG01018832001	4	-2.02	0.00	Cytochrome P450 84A1
GSVIVG01008910001	18	-2.05	0.00	Unkown Protein Function
GSVIVG01015298001	11	-2.05	0.00	Receptor-like protein kinase HSL1
GSVIVG01027857001	5	-2.05	0.00	Phosphate transporter PHO1-3
GSVIVG01027361001	13	-2.06	0.00	Flavanone 3-dioxygenase
GSVIVG01027788001	5	-2.09	0.00	Unkown Protein Function
GSVIVG01015278001	11	-2.10	0.01	emb|CAB79689.1| putative protein
GSVIVG01000144001	7	-2.11	0.00	Peroxidase 73
GSVIVG01024578001	6	-2.12	0.00	Probable serine/threonine-protein kinase WNK4
GSVIVG01019530001	2	-2.14	0.01	Putative RING-H2 finger protein ATL21A
GSVIVG01026627001	4	-2.14	0.01	Kynurenine 3-monooxygenase and related flavoprotein monooxygenases
GSVIVG01011635001	1	-2.16	0.00	F10B6.18
GSVIVG01021723001	10	-2.19	0.00	Reticuline oxidase-like protein
GSVIVG01009045001	18	-2.20	0.00	RNA-binding protein 24

GSVIVG01029160001	11	-2.20	0.00	Brassinosteroid-regulated protein BRU1
GSVIVG01012026001	1	-2.21	0.00	Unkown Protein Function
GSVIVG01017958001	5	-2.22	0.00	Primary amine oxidase
GSVIVG01019841001	2	-2.23	0.00	Pathogenesis-related protein R major form
GSVIVG01037055001	3	-2.25	0.00	Unkown Protein Function
GSVIVG01021397001	10	-2.25	0.01	Probable WRKY transcription factor 28
GSVIVG01008134001	17	-2.27	0.01	Unkown Protein Function
GSVIVG01027787001	5	-2.28	0.00	Unkown Protein Function
GSVIVG01003473001	Un	-2.28	0.00	Zinc finger protein CONSTANS-LIKE 9
GSVIVG01011037001	7	-2.29	0.00	Probable gibberellin receptor GID1L2
GSVIVG01028057001	7	-2.29	0.00	Probable polygalacturonase
GSVIVG01028260001	7	-2.32	0.00	expressed protein
GSVIVG01014147001	19	-2.38	0.00	Probable LRR receptor-like serine/threonine-protein kinase RFK1
GSVIVG01021192001	10	-2.38	0.00	Unkown Protein Function
GSVIVG01033648001	8	-2.39	0.00	Transcription factor RAX2
GSVIVG01019034001	4	-2.39	0.00	Uncharacterized membrane protein YOL092W
GSVIVG01006143001	Un	-2.41	0.01	Cysteine-rich receptor-like protein kinase 29
GSVIVG01034058001	8	-2.42	0.00	Probable glutathione S-transferase
GSVIVG01021407001	10	-2.42	0.00	LRR receptor-like serine/threonine-protein kinase FLS2
GSVIVG01013182001	2	-2.44	0.00	NAC domain-containing protein 78
GSVIVG01004076001	Un	-2.44	0.00	Protein TRANSPARENT TESTA 12
GSVIVG01008324001	17	-2.45	0.01	calmodulin binding
GSVIVG01017223001	9	-2.46	0.01	Probable boron transporter 2
GSVIVG01008094001	17	-2.48	0.00	Germin-like protein subfamily T member 1
GSVIVG01038652001	16	-2.50	0.00	Flavanone 3-dioxygenase
GSVIVG01023804001	3	-2.50	0.00	AMP-activated protein kinase gamma regulatory subunit putative
GSVIVG01013708001	18_random	-2.50	0.01	3-ketoacyl-CoA synthase 11
GSVIVG01009106001	18	-2.55	0.01	Cationic peroxidase 1

GSVIVG01010591001	16	-2.58	0.01	Unkown Protein Function
GSVIVG01022181001	7	-2.58	0.00	L-ascorbate oxidase
GSVIVG01036802001	19	-2.60	0.01	Myb-related protein Zm1
GSVIVG01014340001	19	-2.64	0.00	Metallothionein-like protein type 3
GSVIVG01027568001	15	-2.65	0.00	Gibberellin receptor GID1
GSVIVG01023095001	12	-2.67	0.00	Anthocyanidin 3-O-glucosyltransferase 2
GSVIVG01021309001	10	-2.70	0.00	GDSL esterase/lipase At5g45670
GSVIVG01005168001	Un	-2.71	0.00	Cysteine-rich receptor-like protein kinase 10
GSVIVG01011428001	14	-2.73	0.00	Os09g0413600
GSVIVG01036018001	4	-2.73	0.01	Aspartic proteinase nepenthesin-2
GSVIVG01031737001	3	-2.78	0.00	Ferredoxin--nitrite reductase chloroplastic
GSVIVG01010556001	16	-2.79	0.01	Stilbene synthase 1
GSVIVG01014382001	19	-2.80	0.00	5'-AMP-activated protein kinase gamma subunit
GSVIVG01020228001	1	-2.81	0.00	Probable xyloglucan endotransglucosylase/hydrolase protein 33
GSVIVG01029173001	11	-2.87	0.00	Probable xyloglucan endotransglucosylase/hydrolase protein 23
GSVIVG01037008001	3	-2.91	0.01	Basic form of pathogenesis-related protein 1
GSVIVG01021459001	10	-2.93	0.01	Unkown Protein Function
GSVIVG01016345001	13	-2.93	0.00	RNA-binding protein 24
GSVIVG01027143001	15	-2.94	0.00	O-acyltransferase WSD1
GSVIVG01025391001	6	-2.94	0.00	Unkown Protein Function
GSVIVG01038111001	5	-2.95	0.00	Endochitinase PR4
GSVIVG01003765001	Un	-2.98	0.01	STS14 protein
GSVIVG01010579001	16	-2.98	0.00	Stilbene synthase 4
GSVIVG01010590001	16	-2.98	0.00	Stilbene synthase 3
GSVIVG01029165001	11	-2.98	0.00	Probable xyloglucan endotransglucosylase/hydrolase protein 23
GSVIVG01029163001	11	-2.99	0.00	Probable xyloglucan endotransglucosylase/hydrolase protein 23
GSVIVG01029161001	11	-2.99	0.00	Brassinosteroid-regulated protein BRU1
GSVIVG01001021001	16	-3.01	0.00	Putative RING-H2 finger protein ATL21A

GSVIVG01019835001	2	-3.02	0.00	Thaumatin-like protein
GSVIVG01033538001	8	-3.03	0.00	Glucan endo-13-beta-glucosidase basic isoform
GSVIVG01010561001	16	-3.07	0.00	Stilbene synthase 1
GSVIVG01027144001	15	-3.07	0.00	Unkown Protein Function
GSVIVG01021355001	10	-3.09	0.00	Protein SRG1
GSVIVG01016196001	13	-3.10	0.00	nodulin family protein
GSVIVG01010557001	16	-3.13	0.00	Stilbene synthase 4
GSVIVG01031543001	6	-3.13	0.00	Lichenase
GSVIVG01019848001	2	-3.20	0.00	Pathogenesis-related protein R major form
GSVIVG01010589001	16	-3.21	0.00	Stilbene synthase 3
GSVIVG01020734001	12	-3.23	0.00	Eugenol O-methyltransferase
GSVIVG01010581001	16	-3.24	0.00	Stilbene synthase 4
GSVIVG01030243001	8	-3.24	0.01	Wound-induced protein putative
GSVIVG01007898001	17	-3.25	0.00	Cytokinin-O-glucosyltransferase 2
GSVIVG01019836001	2	-3.28	0.00	Thaumatin-like protein
GSVIVG01037910001	3	-3.31	0.00	secretory protein putative
GSVIVG01005164001	Un	-3.31	0.00	Cysteine-rich receptor-like protein kinase 29
GSVIVG01035055001	5	-3.36	0.00	Major allergen Pru ar 1
GSVIVG01035062001	5	-3.36	0.00	Major allergen Pru ar 1
GSVIVG01035060001	5	-3.36	0.00	Major allergen Pru av 1
GSVIVG01014205001	19	-3.39	0.00	Epidermis-specific secreted glycoprotein EP1
GSVIVG01010582001	16	-3.39	0.00	Stilbene synthase 3
GSVIVG01000580001	1	-3.41	0.00	ABC transporter B family member 15
GSVIVG01016930001	9	-3.42	0.00	Unkown Protein Function
GSVIVG01028935001	16	-3.48	0.00	photoassimilate-responsive protein putative
GSVIVG01019840001	2	-3.50	0.00	Thaumatin-like protein
GSVIVG01020834001	12	-3.51	0.00	NAC domain-containing protein 42
GSVIVG01013272001	2	-3.51	0.00	Beta-amylase 3 chloroplastic
GSVIVG01005840001	Un	-3.57	0.00	Probable NADH dehydrogenase
GSVIVG01021278001	10	-3.59	0.00	Probable LRR receptor-like serine/threonine-protein kinase At1g53430
GSVIVG01010584001	16	-3.59	0.00	Stilbene synthase 4
GSVIVG01036885001	2	-3.59	0.00	Abscisic acid 8'-hydroxylase 1
GSVIVG01017332001	9	-3.59	0.00	Cucumber peeling cupredoxin
GSVIVG01025653001	8	-3.62	0.00	MLO-like protein 12
GSVIVG01010583001	16	-3.63	0.00	Stilbene synthase 4

GSVIVG01015189001	11	-3.64	0.00	Putative transcription factor bHLH041
GSVIVG01031746001	3	-3.68	0.00	Alpha-amylase
GSVIVG01010585001	16	-3.70	0.00	Stilbene synthase 4
GSVIVG01029110001	11	-3.70	0.00	Proteasome subunit alpha type-2-B
GSVIVG01019838001	2	-3.72	0.00	Zeamatin
GSVIVG01010580001	16	-3.76	0.00	Stilbene synthase 2
GSVIVG01035076001	5	-3.76	0.01	Pathogenesis-related protein STH-2
GSVIVG01035059001	5	-3.76	0.00	Major allergen Pru av 1
GSVIVG01027512001	15	-3.77	0.00	Cytochrome P450 87A3
GSVIVG01025284001	6	-3.79	0.00	Unkown Protein Function
GSVIVG01036322001	18_random	-3.84	0.00	(+)-delta-cadinene synthase isozyme A
GSVIVG01020645001	12	-3.88	0.01	Chavicol O-methyltransferase
GSVIVG01025394001	6	-3.88	0.00	Unkown Protein Function
GSVIVG01025782001	8	-3.90	0.00	Probable glutathione S-transferase
GSVIVG01035061001	5	-3.93	0.00	Major allergen Pru av 1
GSVIVG01014970001	19	-4.01	0.00	Potassium transporter 1
GSVIVG01013894001	16	-4.02	0.00	Unkown Protein Function
GSVIVG01010568001	16	-4.05	0.00	Stilbene synthase 6
GSVIVG01026213001	10	-4.05	0.00	Stilbene synthase 1
GSVIVG01020731001	12	-4.08	0.00	O-methyltransferase ZRP4
GSVIVG01014270001	19	-4.09	0.00	Anthocyanidin 3-O-glucosyltransferase
GSVIVG01010578001	16	-4.11	0.00	Stilbene synthase 4
GSVIVG01021797001	14	-4.14	0.00	Calmodulin-related protein
GSVIVG01008670001	18	-4.20	0.00	Cytochrome P450 87A3
GSVIVG01013817001	16	-4.25	0.00	Unkown Protein Function
GSVIVG01010574001	16	-4.27	0.00	Stilbene synthase 4
GSVIVG01006610001	Un	-4.29	0.00	Unkown Protein Function
GSVIVG01009107001	18	-4.32	0.00	Cationic peroxidase 1
GSVIVG01000071001	14	-4.34	0.00	Germin-like protein subfamily 1 member 13
GSVIVG01022205001	7	-4.41	0.00	Cytochrome P450 84A1
GSVIVG01025287001	6	-4.44	0.00	Unkown Protein Function
GSVIVG01027785001	5	-4.48	0.00	Unkown Protein Function
GSVIVG01009108001	18	-4.66	0.00	Cationic peroxidase 1
GSVIVG01031080001	14	-4.68	0.00	Germin-like protein subfamily 1 member 15
GSVIVG01000103001	14	-5.07	0.00	Germin-like protein subfamily 1 member 15
GSVIVG01000098001	14	-5.09	0.00	Germin-like protein subfamily 1

				member 15
GSVIVG01024505001	16	-5.29	0.00	Unkown Protein Function
GSVIVG01020640001	12	-5.29	0.00	Eugenol O-methyltransferase
GSVIVG01008597001	17	-5.43	0.00	Protein RUPTURED POLLEN GRAIN 1
GSVIVG01009109001	18	-5.48	0.00	Cationic peroxidase 1
GSVIVG01000102001	14	-5.68	0.00	Germin-like protein subfamily 1 member 15
GSVIVG01031079001	14	-5.69	0.00	Germin-like protein subfamily 1 member 15
GSVIVG01000097001	14	-6.28	0.00	Germin-like protein subfamily 1 member 15

Gene_ID = Gene code nomenclature based on reference genome annotation PN40024 (12X.v1); log_2(FC)= log base 2 of fold change; p_value = differential expression significance.
