# Supplementary material for: Transcriptome profiling of grapevine seedless segregants during berry development reveals candidate genes associated with berry weight
Source: BMC Plant Biol. 2016 Apr 26;16:104. doi: 10.1186/s12870-016-0789-1 (PMC4845426; doi:10.1186/s12870-016-0789-1)
Supplement: Additional file 8: Table S8. — List of 37 DE genes (p < 0.01) identified in the FST and B68 stages in the comparisons between LB and SB segregants. (PDF 69 kb) [file 12870_2016_789_MOESM8_ESM.pdf]

Table S8. List of 37 DE genes ($p < 0.01$) identified in the FST and B68 stages in the comparisons between LB and SB segregants.

Gene_ID	log_2(FC) FST	p_value FST	log_2(FC) B68	p_value B68	Description
GSVIVG01008910001	-1.7	0.002	-2.0	0.004	Unkown Protein Function
GSVIVG01010556001	-3.7	0.000	-2.8	0.005	Stilbene synthase 1
GSVIVG01010557001	-4.9	0.000	-3.1	0.001	Stilbene synthase 4
GSVIVG01010561001	-2.7	0.004	-3.1	0.003	Stilbene synthase 1
GSVIVG01010568001	-4.2	0.000	-4.0	0.000	Stilbene synthase 6
GSVIVG01010578001	-3.3	0.001	-4.1	0.000	Stilbene synthase 4
GSVIVG01010579001	-3.2	0.001	-3.0	0.003	Stilbene synthase 4
GSVIVG01010580001	-3.0	0.001	-3.8	0.000	Stilbene synthase 2
GSVIVG01010581001	-4.5	0.000	-3.2	0.000	Stilbene synthase 4
GSVIVG01010582001	-3.9	0.000	-3.4	0.000	Stilbene synthase 3
GSVIVG01010583001	-3.2	0.000	-3.6	0.000	Stilbene synthase 4
GSVIVG01010584001	-3.1	0.001	-3.6	0.000	Stilbene synthase 4
GSVIVG01010585001	-4.0	0.000	-3.7	0.000	Stilbene synthase 4
GSVIVG01010589001	-3.5	0.000	-3.2	0.000	Stilbene synthase 3
GSVIVG01010590001	-3.2	0.000	-3.0	0.000	Stilbene synthase 3
GSVIVG01010591001	-3.1	0.001	-2.6	0.007	Unkown Protein Function
GSVIVG01019452001	1.6	0.006	-1.5	0.007	Unkown Protein Function
GSVIVG01021278001	-2.2	0.006	-3.6	0.000	Probable LRR receptor-like serine/threonine-protein kinase At1g53430
GSVIVG01021724001	-1.8	0.005	-1.9	0.004	Unkown Protein Function
GSVIVG01024301001	4.1	0.000	3.4	0.004	Ca2+-dependent lipid-binding protein CLB1/vesicle protein vp115/Granuphilin A contains C2 domain
GSVIVG01025284001	-3.0	0.001	-3.8	0.002	Unkown Protein Function
GSVIVG01025287001	-3.3	0.000	-4.4	0.000	Unkown Protein Function
GSVIVG01025391001	-2.4	0.009	-2.9	0.001	Unkown Protein Function
GSVIVG01025394001	-2.6	0.005	-3.9	0.000	Unkown Protein Function
GSVIVG01026803001	2.2	0.006	1.7	0.001	Isoflavone-7-O-methyltransferase 6
GSVIVG01027558001	-2.3	0.000	-1.7	0.005	Cytochrome P450 76C2
GSVIVG01027568001	-2.1	0.005	-2.7	0.001	Gibberellin receptor GID1

GSVIVG01027785001	-2.4	0.001	-4.5	0.000	Unkown Protein Function
GSVIVG01029110001	-3.1	0.000	-3.7	0.000	Proteasome subunit alpha type-2-B
GSVIVG01029491001	3.1	0.008	3.9	0.002	Lupeol synthase 5
GSVIVG01031746001	-6.3	0.002	-3.7	0.002	Alpha-amylase
GSVIVG01035059001	-1.5	0.005	-3.8	0.000	Major allergen Pru av 1
GSVIVG01035076001	-2.8	0.000	-3.8	0.006	Pathogenesis-related protein STH-2
GSVIVG01036279001	1.3	0.007	-2.0	0.002	Pathogenesis-related protein PR-4B
GSVIVG01036322001	-2.8	0.000	-3.8	0.000	(+)-delta-cadinene synthase isozyme A
GSVIVG01036885001	-2.2	0.004	-3.6	0.000	Abscisic acid 8'-hydroxylase 1
GSVIVG01037055001	1.3	0.010	-2.2	0.001	Unkown Protein Function

Gene_ID= Gene code nomenclature based on reference genome annotation PN40024 (12X.v1); log_2(FC) FST= log base 2 of fold change observed in FST stage; p_value FST= differential expression significance in the FST stage; log_2(FC) B68= log base 2 of fold change observed in the B68 stage; p_value B68= differential expression significance in the B68 stage.
