# Supplementary material for: Transcriptome profiling of grapevine seedless segregants during berry development reveals candidate genes associated with berry weight
Source: BMC Plant Biol. 2016 Apr 26;16:104. doi: 10.1186/s12870-016-0789-1 (PMC4845426; doi:10.1186/s12870-016-0789-1)
Supplement: Additional file 9: Figure S1. — A, B, C, D, E. GO enrichment of five clusters identified in hierarchical clustering of 526 DE genes, from comparison between LB and SB segregants. A. GO categories over-represented in Cluster 1 (Biological Process); B. GO categories over-represented in Cluster 3 (Biological Process); C. GO categories over-represented in Cluster 4 (Molecular Function); D. GO categories over-represented in Cluster 5 (Biological Process); E. GO categories over-represented in Cluster 5 (Molecular Function). Biological Process and Molecular Function categories are shown, and only significantly over-represented categories were considered (p < 0.05 and FDR < 0.05). The analysis was performed using the online agriGO tool and the GO complete category. The boxes contain the GO number, the p-value (in parentheses), the category description, the number of genes in each category associated with the GO term versus the total of query genes and the number of genes in each category out of 14,511 genes of the reference genome of Vitis vinifera (PN40024, 12X.v1), with associated GO terms. The arrows indicate the relationships among the GO categories, as follows: black solid arrows mean that a GO category is also included in the other one; red solid arrows mean that one GO category positively regulates the other; green solid arrows mean that the GO category negatively regulates the other; black dashed arrows indicate that there are two significant nodes related to the GO category; and black dotted arrows indicate that only one significant node is related to the GO category. (PDF 567 kb) [file 12870_2016_789_MOESM9_ESM.pdf]

Gene ontology (GO) enrichments were developed using the five clusters identified by the hierarchical clustering analysis of the group of 526 DE genes, using the agriGO platform [42]. These genes were obtained from comparison between LB and SB segregants, in the FTS and B68 stages.

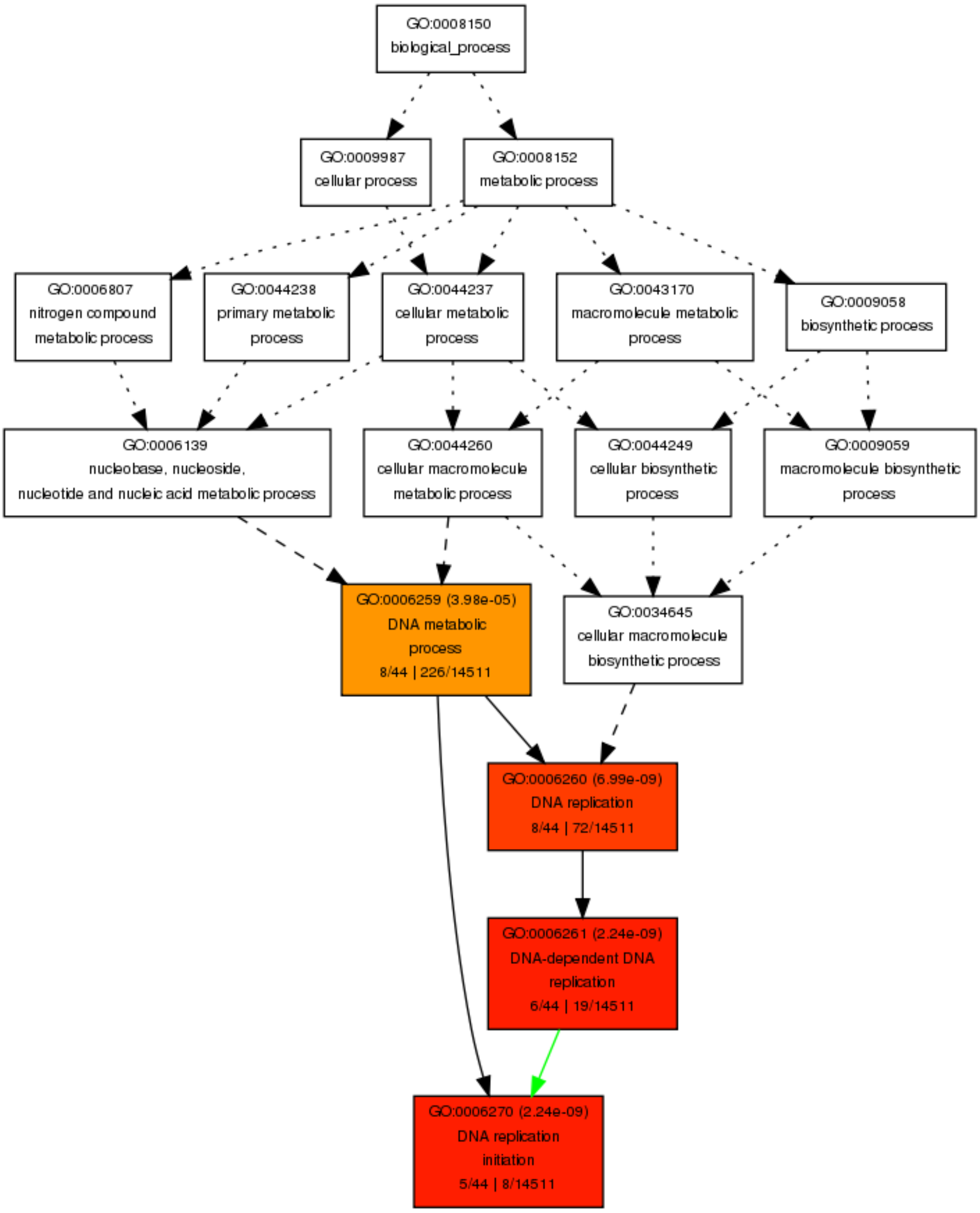

Figure S1A

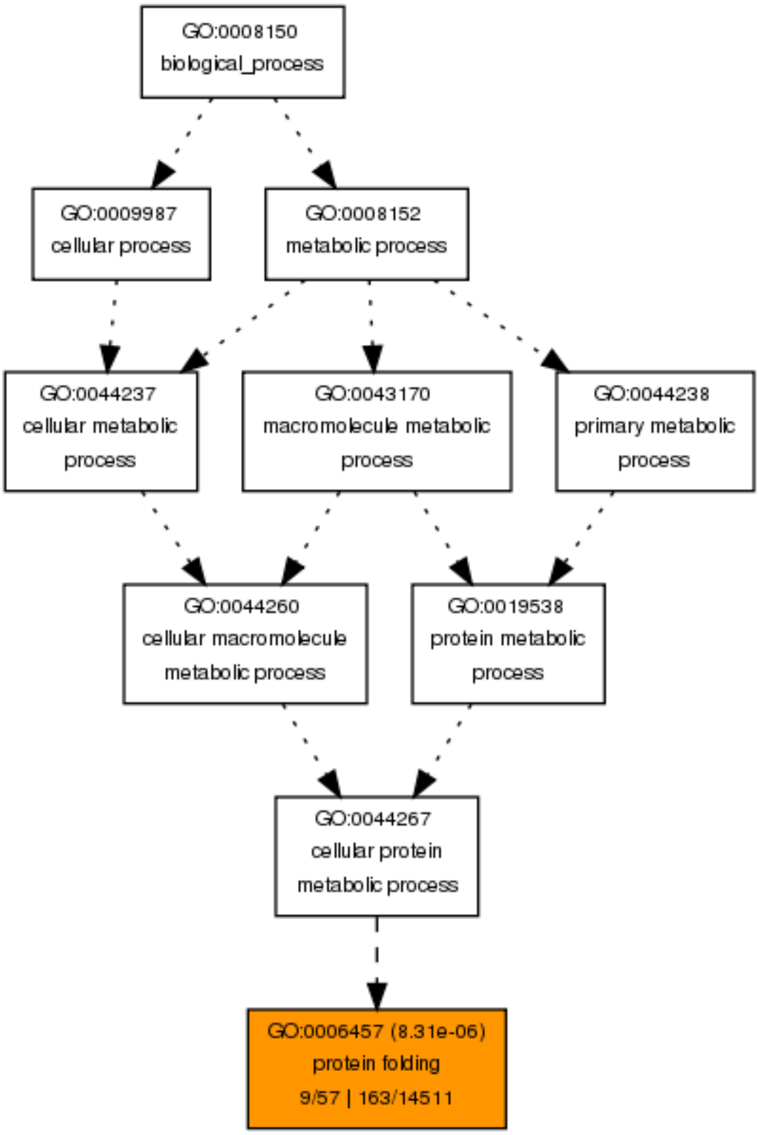
GO:0008150
biological_process
GO:0009987
cellular process
GO:0008152
metabolic process
GO:0044237
cellular metabolic process
GO:0043170
macromolecule metabolic process
GO:0044238
primary metabolic process
GO:0044260
cellular macromolecule metabolic process
GO:0019538
protein metabolic process
GO:0044267
cellular protein metabolic process
GO:0006457 (8.31e-06)
protein folding
9/57 | 163/14511

Figure S1B

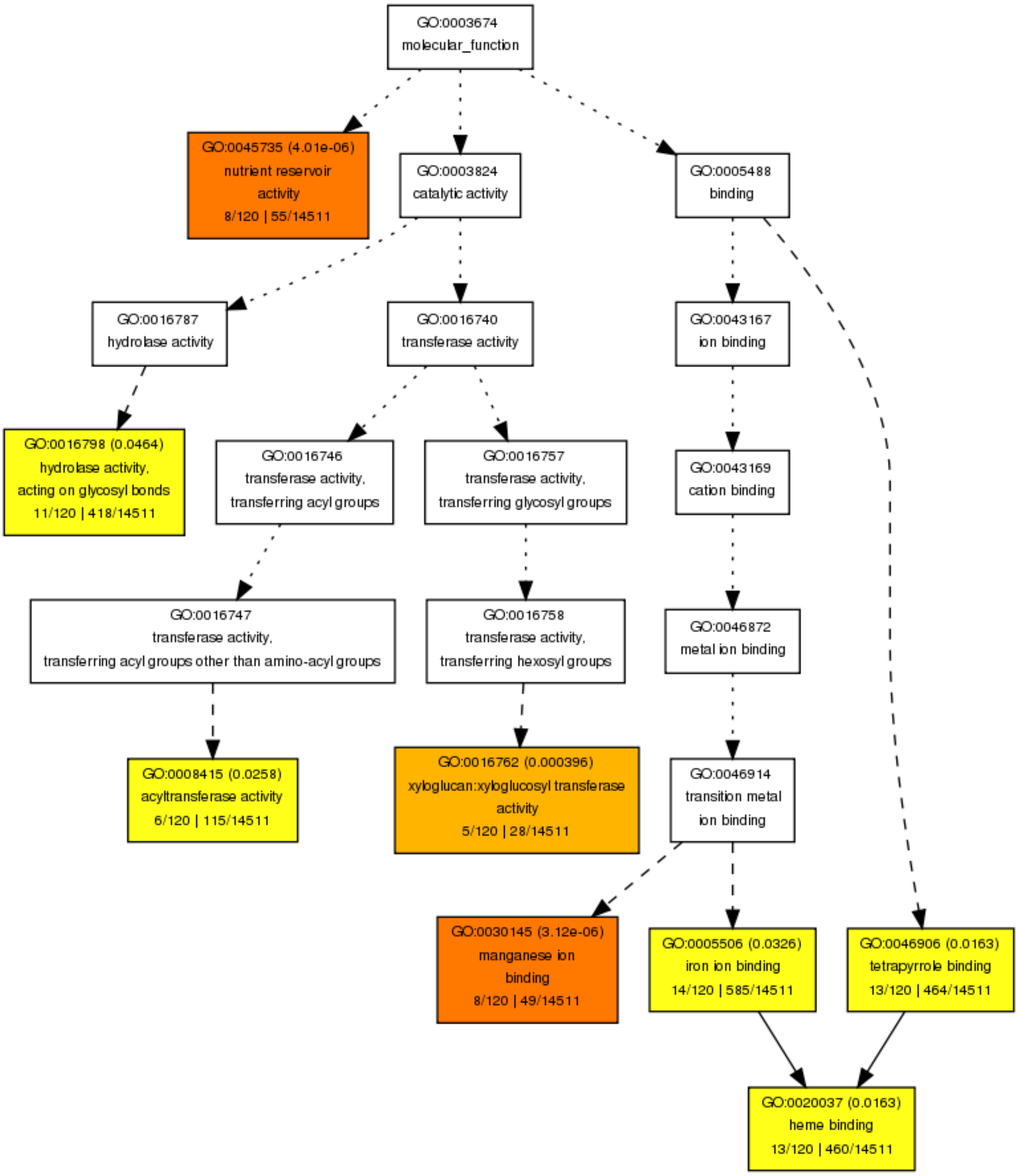

GO:0003674
molecular_function
GO:0045735 (4.01e-06)
nutrient reservoir
activity
8/120 | 55/14511
GO:0003824
catalytic activity
GO:0005488
binding
GO:0016787
hydrolase activity
GO:0016740
transferase activity
GO:0043167
ion binding
GO:0016798 (0.0464)
hydrolase activity,
acting on glycosyl bonds
11/120 | 418/14511
GO:0016746
transferase activity,
transferring acyl groups
GO:0016757
transferase activity,
transferring glycosyl groups
GO:0043169
cation binding
GO:0016747
transferase activity,
transferring acyl groups other than amino-acyl groups
GO:0016758
transferase activity,
transferring hexosyl groups
GO:0046872
metal ion binding
GO:0008415 (0.0258)
acyltransferase activity
6/120 | 115/14511
GO:0016762 (0.000396)
xyloglucan:xyloglucosyl transferase
activity
5/120 | 28/14511
GO:0046914
transition metal
ion binding
GO:0030145 (3.12e-06)
manganese ion
binding
8/120 | 49/14511
GO:0005506 (0.0326)
iron ion binding
14/120 | 585/14511
GO:0046906 (0.0163)
tetrapyrrole binding
13/120 | 464/14511
GO:0020037 (0.0163)
heme binding
13/120 | 460/14511

Figure S1C

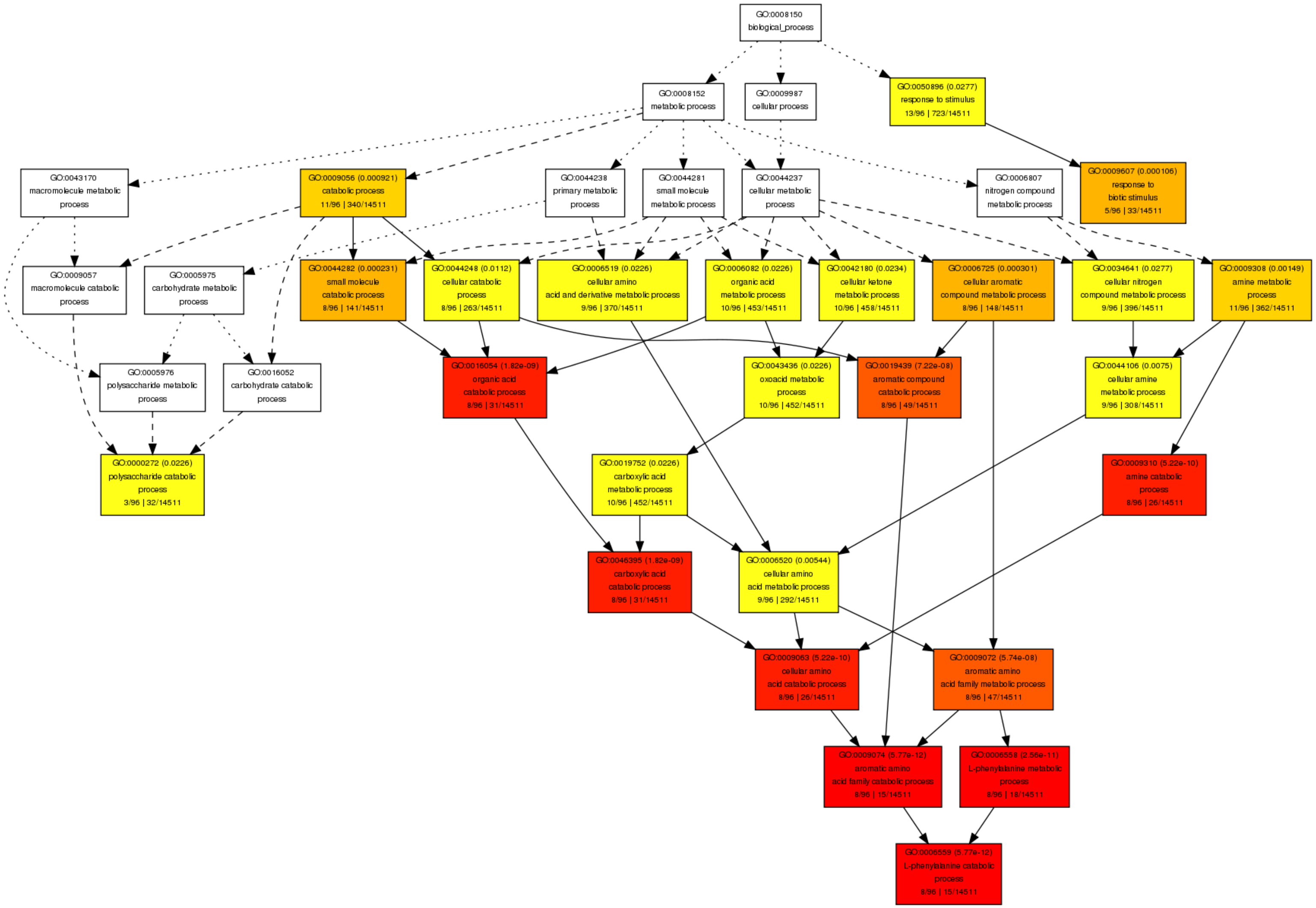
GO:0008150 biological_process
GO:0008152 metabolic process
GO:0009987 cellular process
GO:0050896 (0.0277) response to stimulus 13/96 | 723/14511
GO:0009607 (0.000106) response to biotic stimulus 5/96 | 33/14511
GO:0043170 macromolecule metabolic process
GO:0009056 (0.000921) catabolic process 11/96 | 340/14511
GO:0044238 primary metabolic process
GO:0044281 small molecule metabolic process
GO:0044237 cellular metabolic process
GO:0006807 nitrogen compound metabolic process
GO:0009057 macromolecule catabolic process
GO:0005975 carbohydrate metabolic process
GO:0044282 (0.000231) small molecule catabolic process 8/96 | 141/14511
GO:0044248 (0.0112) cellular catabolic process 8/96 | 263/14511
GO:0006519 (0.0226) cellular amino acid and derivative metabolic process 9/96 | 370/14511
GO:0006082 (0.0226) organic acid metabolic process 10/96 | 453/14511
GO:0042180 (0.0234) cellular ketone metabolic process 10/96 | 458/14511
GO:0006725 (0.000301) cellular aromatic compound metabolic process 8/96 | 148/14511
GO:0034641 (0.0277) cellular nitrogen compound metabolic process 9/96 | 396/14511
GO:0009308 (0.00149) amine metabolic process 11/96 | 362/14511
GO:0005976 polysaccharide metabolic process
GO:0016052 carbohydrate catabolic process
GO:0016054 (1.82e-09) organic acid catabolic process 8/96 | 31/14511
GO:0043436 (0.0226) oxoacid metabolic process 10/96 | 452/14511
GO:0019439 (7.22e-08) aromatic compound catabolic process 8/96 | 49/14511
GO:0044106 (0.0075) cellular amine metabolic process 9/96 | 308/14511
GO:0000272 (0.0226) polysaccharide catabolic process 3/96 | 32/14511
GO:0019752 (0.0226) carboxylic acid metabolic process 10/96 | 452/14511
GO:0009310 (5.22e-10) amine catabolic process 8/96 | 26/14511
GO:0046395 (1.82e-09) carboxylic acid catabolic process 8/96 | 31/14511
GO:0006520 (0.00544) cellular amino acid metabolic process 9/96 | 292/14511
GO:0009063 (5.22e-10) cellular amino acid catabolic process 8/96 | 26/14511
GO:0009072 (5.74e-08) aromatic amino acid family metabolic process 8/96 | 47/14511
GO:0009074 (5.77e-12) aromatic amino acid family catabolic process 8/96 | 15/14511
GO:0006558 (2.56e-11) L-phenylalanine metabolic process 8/96 | 18/14511
GO:0006559 (5.77e-12) L-phenylalanine catabolic process 8/96 | 15/14511

Figure S1D

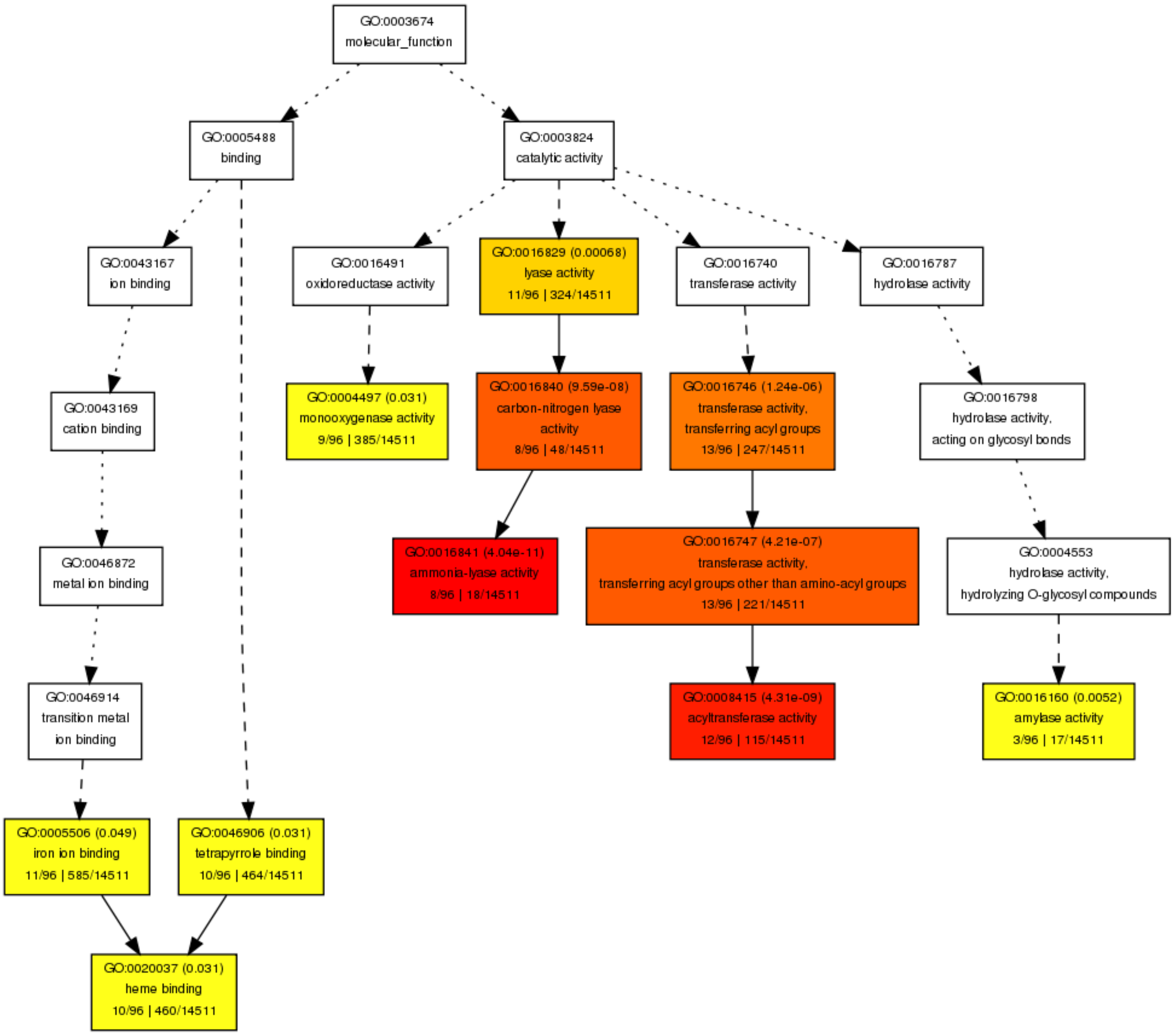

Figure S1E

Figure S1 A, B, C, D, E. GO enrichment of five clusters identified in hierarchical clustering of 526 DE genes, derived from comparison between LB and SB segregants. **A.** GO categories over-represented in Cluster 1 (Biological Process); **B.** GO categories over-represented in Cluster 3 (Biological Process); **C.** GO categories over-represented in Cluster 4 (Molecular Function); **D.** GO categories over-represented in Cluster 5 (Biological Process); **E.** GO categories over-represented in

Cluster 5 (Molecular Function). Biological Process and Molecular Function categories are shown, and only significantly over-represented categories were considered ($p < 0.05$ and FDR < 0.05). The analysis was performed using the online agriGO tool and the GO complete category. The boxes contain the GO number, the p-value (in parentheses), the category description, the number of genes in each category associated with the GO term versus the total of query genes and the number of genes in each category out of 14,511 genes of the reference genome of *Vitis vinifera* (PN40024, 12X.v1), with associated GO terms. The arrows indicate the relationships among the GO categories, as follows: black solid arrows mean that a GO category is also included in the other one; red solid arrows mean that one GO category positively regulates the other; green solid arrows mean that the GO category negatively regulates the other; black dashed arrows indicate that there are two significant nodes related to the GO category; and black dotted arrows indicate that only one significant node is related to the GO category.
