# Supplementary material for: Transcriptome profiling of grapevine seedless segregants during berry development reveals candidate genes associated with berry weight
Source: BMC Plant Biol. 2016 Apr 26;16:104. doi: 10.1186/s12870-016-0789-1 (PMC4845426; doi:10.1186/s12870-016-0789-1)
Supplement: Additional file 10: Figure S2. — Functional characterization of 68 candidate genes significantly correlated with PCA component 1, associated with differences between LB and SB segregants. (PDF 96 kb) [file 12870_2016_789_MOESM10_ESM.pdf]

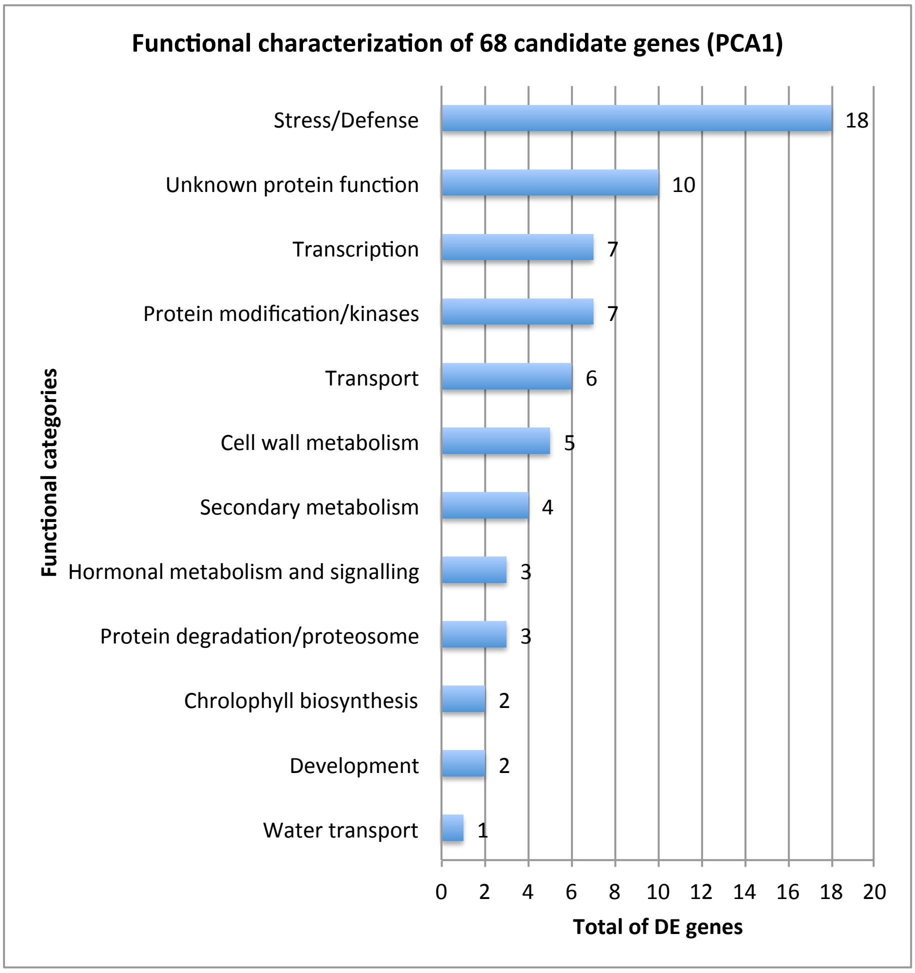

Figure S2. Functional characterization of 68 candidate genes significantly correlated with PCA component 1, associated with differences between LB and SB segregants.
