# Supplementary material for: Transcriptome profiling of grapevine seedless segregants during berry development reveals candidate genes associated with berry weight
Source: BMC Plant Biol. 2016 Apr 26;16:104. doi: 10.1186/s12870-016-0789-1 (PMC4845426; doi:10.1186/s12870-016-0789-1)
Supplement: Additional file 11: Figure S3. — Functional characterization of 16 candidate genes significantly correlated with PCA component 2, associated with differences between FST and B68 stages. (PDF 87 kb) [file 12870_2016_789_MOESM11_ESM.pdf]

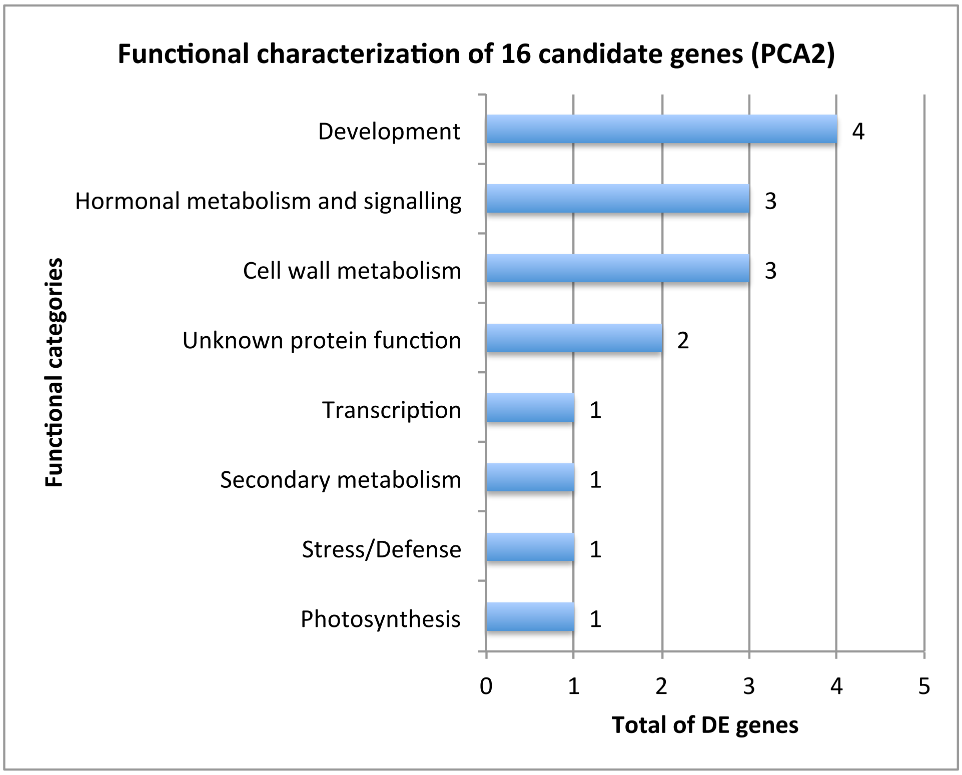

Figure S3. Functional characterization of 16 candidate genes significantly correlated with PCA component 2, associated with differences between FST and B68 stages.
