# Supplementary material for: Transcriptome profiling of grapevine seedless segregants during berry development reveals candidate genes associated with berry weight
Source: BMC Plant Biol. 2016 Apr 26;16:104. doi: 10.1186/s12870-016-0789-1 (PMC4845426; doi:10.1186/s12870-016-0789-1)
Supplement: Additional file 12: Figure S4. — Correlogram representing a total of 4,950 partial correlations, including significant and non-significant correlations, found among the group of 100 DE genes with the highest significance, associated with differences between LB and SB segregants, in the FST and B68 stages. The color indicates the type of correlation i.e., negative significant correlations are in red while positive significant correlations are shown in blue. Intensity of colors indicates strength of correlations; darker shades represent higher or more negative values. (PDF 174 kb) [file 12870_2016_789_MOESM12_ESM.pdf]

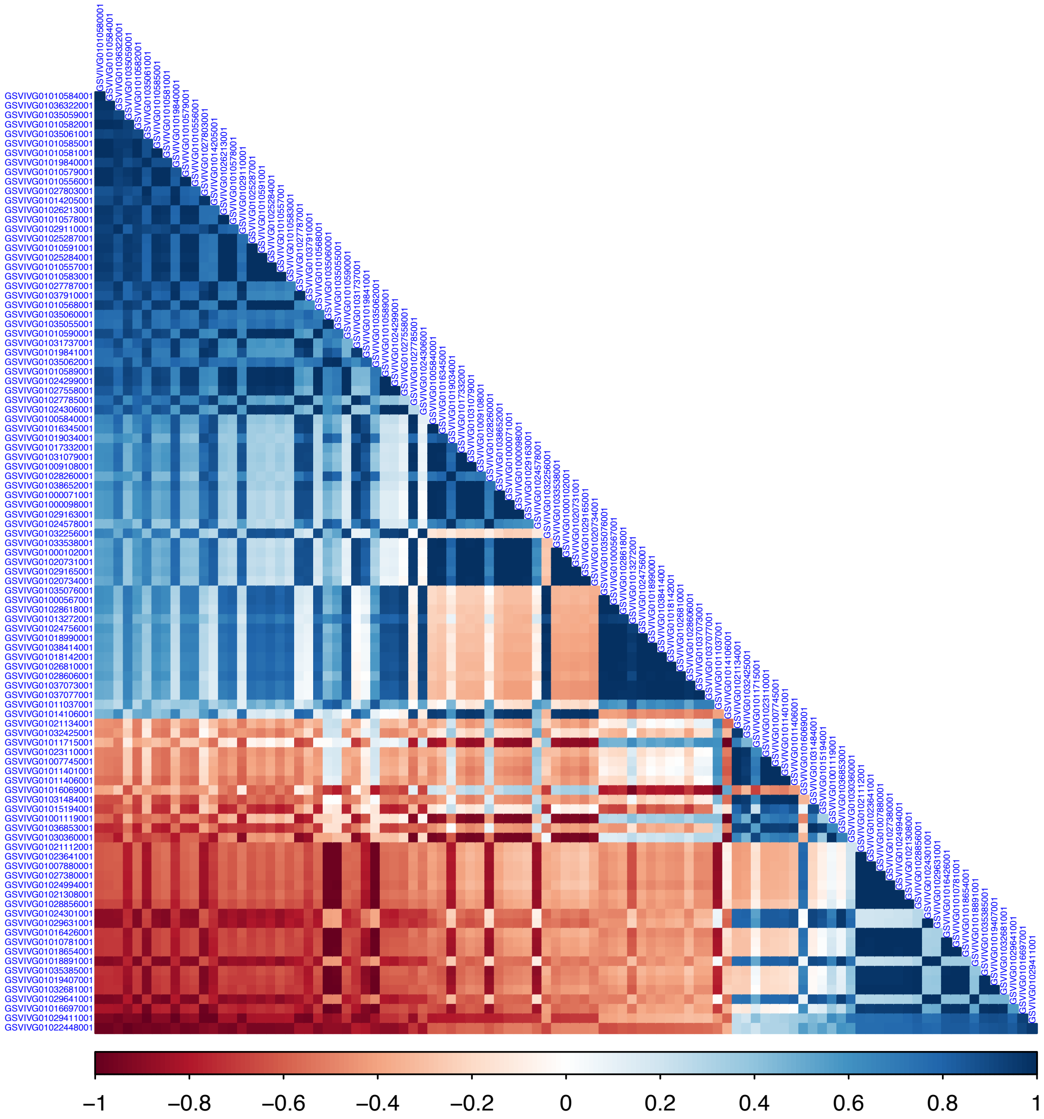

Figure S4. Correlogram representing a total of 4,950 partial correlations, including significant and non-significant correlations, found among the group of 100 DE genes with the highest significance, associated with differences between LB and SB segregants, in the FST and B68 stages. The color indicates the type of correlation *i.e.* negative significant correlations are in red while positive significant correlations are shown in blue. Intensity of colors indicates strength of correlations; darker shades represent higher or more negative values.
