# Supplementary material for: Transcriptome profiling of grapevine seedless segregants during berry development reveals candidate genes associated with berry weight
Source: BMC Plant Biol. 2016 Apr 26;16:104. doi: 10.1186/s12870-016-0789-1 (PMC4845426; doi:10.1186/s12870-016-0789-1)
Supplement: Additional file 13: Figure S5. — Correlogram representing 431 significant correlations (p < 0.05), found among the group of 100 DE genes with the highest significance, associated with differences between LB and SB segregants in the FST and B68 stages. Correlograms were plotted with the total of observed correlations. The color indicates the type of correlation i.e., negative significant correlations are in red while positive significant correlations are shown in blue. Intensity of colors indicates strength of correlations; darker shades represent higher or more negative values. (PDF 132 kb) [file 12870_2016_789_MOESM13_ESM.pdf]

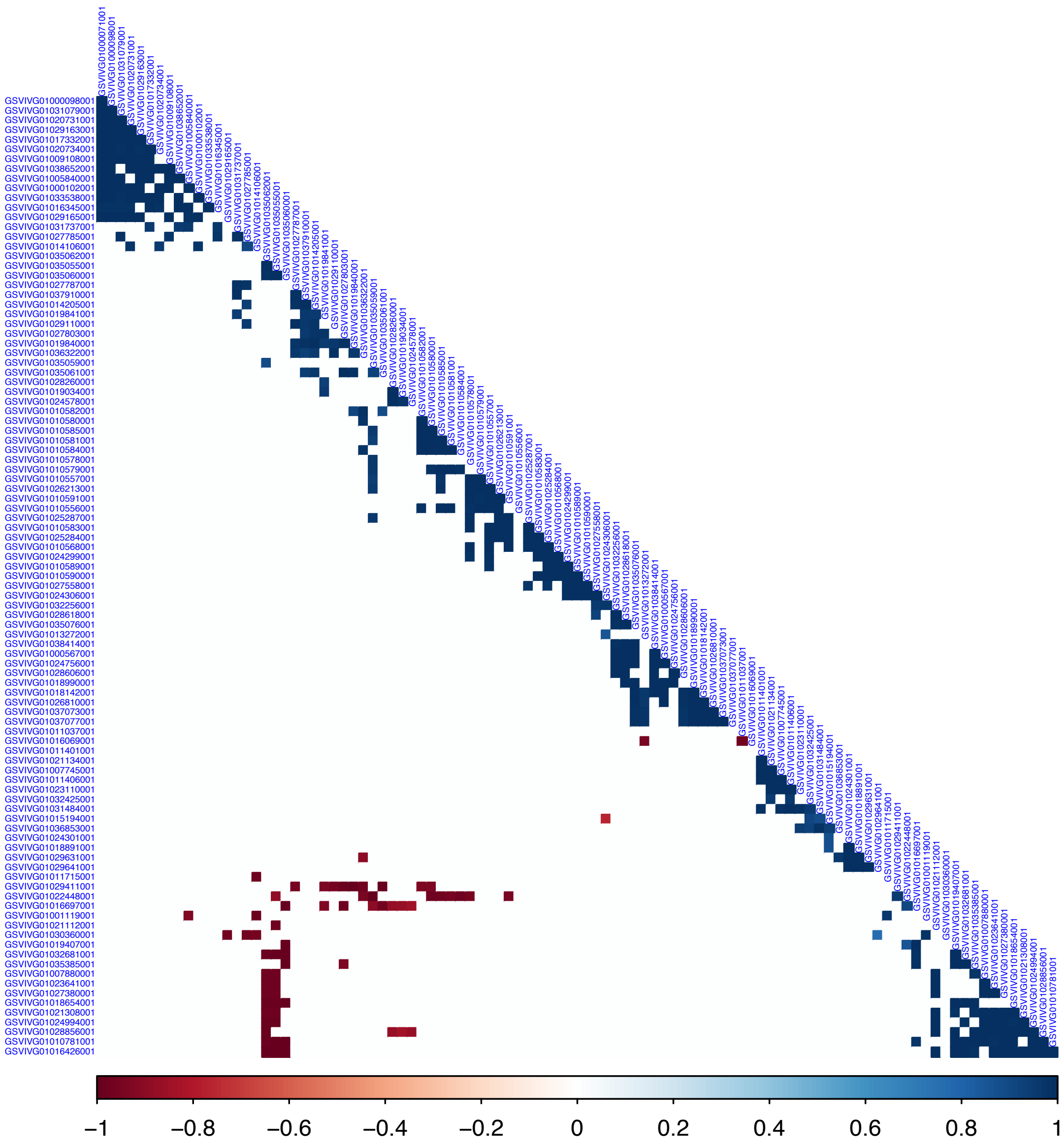

Figure S5. Correlogram representing 431 significant correlations ($p < 0.05$), found among the group of 100 DE genes with the highest significance, associated with differences between LB and SB segregants in the FST and B68 stages. Correlograms were plotted with the total of observed correlations. The color indicates the type of correlation *i.e.* negative significant correlations are in red while positive significant correlations are shown in blue. Intensity of colors indicates strength of correlations; darker shades represent higher or more negative values.
