# Supplementary material for: Transcriptome profiling of grapevine seedless segregants during berry development reveals candidate genes associated with berry weight
Source: BMC Plant Biol. 2016 Apr 26;16:104. doi: 10.1186/s12870-016-0789-1 (PMC4845426; doi:10.1186/s12870-016-0789-1)
Supplement: Additional file 14: Table S9. — Summary of significant partial correlations observed in the group of 100 DE genes with high significance, associated with differences between LB and SB segregants, in the FST and B68 stages. (PDF 295 kb) [file 12870_2016_789_MOESM14_ESM.pdf]

Table S9. **Summary of significant partial correlations observed in the group of 100 DE genes with high significance, associated with differences between LB and SB segregants, in the FST and B68 stages.**

ID ranking	Description	ID ranking	Description	Corr.
**2**	Stilbene synthase 6	**4**	Unkown Protein Function	**1.00**
**3**	Cytokinin dehydrogenase 3	**6**	Ca2+-dependent lipid-binding protein CLB1/vesicle protein vp115/Granuphilin A contains C2 domain	**0.88**
**2**	Stilbene synthase 6	**7**	Stilbene synthase 3	**1.00**
**5**	Stilbene synthase 4	**8**	Stilbene synthase 3	**1.00**
**1**	pore-forming toxin-like protein Hfr-2	**9**	4-hydroxyphenylacetaldehyde oxime monooxygenase	**1.00**
**2**	Stilbene synthase 6	**10**	Stilbene synthase 3	**1.00**
**7**	Stilbene synthase 3	**10**	Stilbene synthase 3	**1.00**
**2**	Stilbene synthase 6	**11**	Stilbene synthase 4	**1.00**
**4**	Unkown Protein Function	**11**	Stilbene synthase 4	**1.00**
**10**	Stilbene synthase 3	**11**	Stilbene synthase 4	**0.99**
**2**	Stilbene synthase 6	**13**	Stilbene synthase 4	**0.99**
**7**	Stilbene synthase 3	**13**	Stilbene synthase 4	**0.98**
**11**	Stilbene synthase 4	**13**	Stilbene synthase 4	**0.99**
**1**	pore-forming toxin-like protein Hfr-2	**15**	Pathogenesis-related protein STH-2	**1.00**
**9**	4-hydroxyphenylacetaldehyde oxime monooxygenase	**15**	Pathogenesis-related protein STH-2	**1.00**
**12**	PR6 protease inhibitor	**15**	Pathogenesis-related protein STH-2	**1.00**
**1**	pore-forming toxin-like protein Hfr-2	**16**	Cytochrome P450 76C2	**0.93**
**4**	Unkown Protein Function	**16**	Cytochrome P450 76C2	**0.97**
**7**	Stilbene synthase 3	**16**	Cytochrome P450 76C2	**1.00**
**10**	Stilbene synthase 3	**16**	Cytochrome P450 76C2	**1.00**
**8**	Stilbene synthase 3	**17**	(+)-delta-cadinene synthase isozyme A	**0.98**
**12**	PR6 protease inhibitor	**18**	Protein WAX2	**1.00**
**15**	Pathogenesis-related protein STH-2	**18**	Protein WAX2	**0.99**
**14**	Isoflavone-7-O-methyltransferase 9	**19**	Vacuolar amino acid transporter 1	**1.00**
**1**	Pore-forming toxin-like protein Hfr-2	**20**	Flavin-containing monooxygenase YUCCA6	**0.99**
**9**	4-hydroxyphenylacetaldehyde oxime monooxygenase	**20**	Flavin-containing monooxygenase YUCCA6	**0.99**
**12**	PR6 protease inhibitor	**20**	Flavin-containing monooxygenase YUCCA6	**1.00**
**15**	Pathogenesis-related protein STH-2	**20**	Flavin-containing monooxygenase YUCCA6	**1.00**

**18**	Protein WAX2	**20**	Flavin-containing monooxygenase YUCCA6	**1.00**
**5**	Stilbene synthase 4	**21**	Stilbene synthase 4	**1.00**
**8**	Stilbene synthase 3	**21**	Stilbene synthase 4	**0.99**
**13**	Stilbene synthase 4	**21**	Stilbene synthase 4	**0.98**
**6**	Ca2+-dependent lipid-binding protein CLB1/vesicle protein vp115/Granuphilin A contains C2 domain	**22**	60S ribosomal protein L7-3	**1.00**
**4**	Unkown Protein Function	**23**	Stilbene synthase 1	**0.99**
**11**	Stilbene synthase 4	**23**	Stilbene synthase 1	**0.99**
**13**	Stilbene synthase 4	**23**	Stilbene synthase 1	**1.00**
**21**	Stilbene synthase 4	**23**	Stilbene synthase 1	**0.99**
**5**	Stilbene synthase 4	**24**	Stilbene synthase 1	**0.99**
**8**	Stilbene synthase 3	**24**	Stilbene synthase 1	**0.98**
**13**	Stilbene synthase 4	**24**	Stilbene synthase 1	**0.99**
**21**	Stilbene synthase 4	**24**	Stilbene synthase 1	**1.00**
**23**	Stilbene synthase 1	**24**	Stilbene synthase 1	**1.00**
**17**	(+)-delta-cadinene synthase isozyme A	**25**	Proteasome subunit alpha type-2-B	**0.98**
**3**	Cytokinin dehydrogenase 3	**26**	Phenylalanine ammonia-lyase	**-0.77**
**7**	Stilbene synthase 3	**26**	Phenylalanine ammonia-lyase	**0.99**
**10**	Stilbene synthase 3	**26**	Phenylalanine ammonia-lyase	**0.99**
**16**	Cytochrome P450 76C2	**26**	Phenylalanine ammonia-lyase	**1.00**
**6**	Ca2+-dependent lipid-binding protein CLB1/vesicle protein vp115/Granuphilin A contains C2 domain	**27**	60S ribosomal protein L7-4	**1.00**
**17**	(+)-delta-cadinene synthase isozyme A	**27**	60S ribosomal protein L7-4	**-0.89**
**22**	60S ribosomal protein L7-3	**27**	60S ribosomal protein L7-4	**0.99**
**1**	pore-forming toxin-like protein Hfr-2	**28**	Cytochrome P450 71B35	**1.00**
**9**	4-hydroxyphenylacetaldehyde oxime monooxygenase	**28**	Cytochrome P450 71B35	**1.00**
**12**	PR6 protease inhibitor	**28**	Cytochrome P450 71B35	**0.99**
**15**	Pathogenesis-related protein STH-2	**28**	Cytochrome P450 71B35	**1.00**
**18**	Protein WAX2	**28**	Cytochrome P450 71B35	**0.99**
**20**	Flavin-containing monooxygenase YUCCA6	**28**	Cytochrome P450 71B35	**0.99**
**12**	PR6 protease inhibitor	**29**	Eugenol synthase 1	**1.00**
**15**	Pathogenesis-related protein STH-2	**29**	Eugenol synthase 1	**0.99**
**18**	Protein WAX2	**29**	Eugenol synthase 1	**1.00**
**20**	Flavin-containing monooxygenase YUCCA6	**29**	Eugenol synthase 1	**1.00**
**5**	Stilbene synthase 4	**30**	Stilbene synthase 4	**1.00**
**8**	Stilbene synthase 3	**30**	Stilbene synthase 4	**1.00**

**17**	(+)-delta-cadinene synthase isozyme A	**30**	Stilbene synthase 4	**0.98**
**21**	Stilbene synthase 4	**30**	Stilbene synthase 4	**0.99**
**3**	Cytokinin dehydrogenase 3	**31**	Cysteine-rich receptor-like protein kinase 10	**0.93**
**27**	60S ribosomal protein L7-4	**31**	Cysteine-rich receptor-like protein kinase 10	**0.96**
**2**	Stilbene synthase 6	**32**	Stilbene synthase 4	**1.00**
**4**	Unkown Protein Function	**32**	Stilbene synthase 4	**1.00**
**11**	Stilbene synthase 4	**32**	Stilbene synthase 4	**1.00**
**13**	Stilbene synthase 4	**32**	Stilbene synthase 4	**1.00**
**23**	Stilbene synthase 1	**32**	Stilbene synthase 4	**1.00**
**24**	Stilbene synthase 1	**32**	Stilbene synthase 4	**0.99**
**14**	Isoflavone-7-O-methyltransferase 9	**33**	Vacuolar amino acid transporter 1	**0.99**
**19**	Vacuolar amino acid transporter 1	**33**	Vacuolar amino acid transporter 1	**1.00**
**2**	Stilbene synthase 6	**34**	Phenylalanine ammonia-lyase 2	**1.00**
**7**	Stilbene synthase 3	**34**	Phenylalanine ammonia-lyase 2	**1.00**
**10**	Stilbene synthase 3	**34**	Phenylalanine ammonia-lyase 2	**1.00**
**11**	Stilbene synthase 4	**34**	Phenylalanine ammonia-lyase 2	**0.99**
**13**	Stilbene synthase 4	**34**	Phenylalanine ammonia-lyase 2	**0.98**
**16**	Cytochrome P450 76C2	**34**	Phenylalanine ammonia-lyase 2	**0.99**
**26**	Phenylalanine ammonia-lyase	**34**	Phenylalanine ammonia-lyase 2	**0.99**
**32**	Stilbene synthase 4	**34**	Phenylalanine ammonia-lyase 2	**0.98**
**5**	Stilbene synthase 4	**35**	F-box/LRR-repeat protein At3g48880	**-0.96**
**17**	(+)-delta-cadinene synthase isozyme A	**35**	F-box/LRR-repeat protein At3g48880	**-0.95**
**21**	Stilbene synthase 4	**35**	F-box/LRR-repeat protein At3g48880	**-0.97**
**30**	Stilbene synthase 4	**35**	F-box/LRR-repeat protein At3g48880	**-0.97**
**32**	Stilbene synthase 4	**35**	F-box/LRR-repeat protein At3g48880	**-0.95**
**14**	Isoflavone-7-O-methyltransferase 9	**36**	Carbonic anhydrase chloroplastic	**1.00**
**19**	Vacuolar amino acid transporter 1	**36**	Carbonic anhydrase chloroplastic	**1.00**
**33**	Vacuolar amino acid transporter 1	**36**	Carbonic anhydrase chloroplastic	**1.00**
**3**	Cytokinin dehydrogenase 3	**37**	Galactinol--sucrose galactosyltransferase	**0.92**
**14**	Isoflavone-7-O-methyltransferase 9	**37**	Galactinol--sucrose galactosyltransferase	**0.96**
**31**	Cysteine-rich receptor-like protein kinase 10	**37**	Galactinol--sucrose galactosyltransferase	**0.93**
**33**	Vacuolar amino acid transporter 1	**37**	Galactinol--sucrose galactosyltransferase	**0.96**
**5**	Stilbene synthase 4	**38**	Stilbene synthase 4	**0.99**
**13**	Stilbene synthase 4	**38**	Stilbene synthase 4	**0.99**
**21**	Stilbene synthase 4	**38**	Stilbene synthase 4	**1.00**
**23**	Stilbene synthase 1	**38**	Stilbene synthase 4	**1.00**

**24**	Stilbene synthase 1	**38**	Stilbene synthase 4	**1.00**
**30**	Stilbene synthase 4	**38**	Stilbene synthase 4	**0.99**
**12**	PR6 protease inhibitor	**39**	Eugenol synthase 1	**1.00**
**15**	Pathogenesis-related protein STH-2	**39**	Eugenol synthase 1	**0.99**
**18**	Protein WAX2	**39**	Eugenol synthase 1	**1.00**
**20**	Flavin-containing monooxygenase YUCCA6	**39**	Eugenol synthase 1	**1.00**
**29**	Eugenol synthase 1	**39**	Eugenol synthase 1	**1.00**
**5**	Stilbene synthase 4	**41**	Stilbene synthase 2	**1.00**
**8**	Stilbene synthase 3	**41**	Stilbene synthase 2	**1.00**
**17**	(+)-delta-cadinene synthase isozyme A	**41**	Stilbene synthase 2	**0.98**
**21**	Stilbene synthase 4	**41**	Stilbene synthase 2	**1.00**
**30**	Stilbene synthase 4	**41**	Stilbene synthase 2	**1.00**
**35**	F-box/LRR-repeat protein At3g48880	**41**	Stilbene synthase 2	**-0.98**
**38**	Stilbene synthase 4	**41**	Stilbene synthase 2	**0.99**
**25**	Proteasome subunit alpha type-2-B	**42**	Unkown Protein Function	**0.95**
**1**	pore-forming toxin-like protein Hfr-2	**43**	Unkown Protein Function	**1.00**
**9**	4-hydroxyphenylacetaldehyde oxime monooxygenase	**43**	Unkown Protein Function	**1.00**
**12**	PR6 protease inhibitor	**43**	Unkown Protein Function	**0.99**
**18**	Protein WAX2	**43**	Unkown Protein Function	**0.98**
**29**	Eugenol synthase 1	**43**	Unkown Protein Function	**0.97**
**39**	Eugenol synthase 1	**43**	Unkown Protein Function	**0.97**
**2**	Stilbene synthase 6	**44**	Unkown Protein Function	**1.00**
**4**	Unkown Protein Function	**44**	Unkown Protein Function	**1.00**
**11**	Stilbene synthase 4	**44**	Unkown Protein Function	**1.00**
**13**	Stilbene synthase 4	**44**	Unkown Protein Function	**1.00**
**23**	Stilbene synthase 1	**44**	Unkown Protein Function	**1.00**
**32**	Stilbene synthase 4	**44**	Unkown Protein Function	**1.00**
**35**	F-box/LRR-repeat protein At3g48880	**44**	Unkown Protein Function	**-0.94**
**38**	Stilbene synthase 4	**44**	Unkown Protein Function	**0.99**
**14**	Isoflavone-7-O-methyltransferase 9	**45**	Glutathione S-transferase zeta class	**0.99**
**31**	Cysteine-rich receptor-like protein kinase 10	**45**	Glutathione S-transferase zeta class	**0.94**
**33**	Vacuolar amino acid transporter 1	**45**	Glutathione S-transferase zeta class	**1.00**
**36**	Carbonic anhydrase chloroplastic	**45**	Glutathione S-transferase zeta class	**1.00**
**2**	Stilbene synthase 6	**46**	Unkown Protein Function	**1.00**
**4**	Unkown Protein Function	**46**	Unkown Protein Function	**1.00**
**7**	Stilbene synthase 3	**46**	Unkown Protein Function	**0.99**
**10**	Stilbene synthase 3	**46**	Unkown Protein Function	**0.99**
**11**	Stilbene synthase 4	**46**	Unkown Protein Function	**1.00**

**13**	Stilbene synthase 4	**46**	Unkown Protein Function	**0.99**
**16**	Cytochrome P450 76C2	**46**	Unkown Protein Function	**0.98**
**23**	Stilbene synthase 1	**46**	Unkown Protein Function	**0.99**
**34**	Phenylalanine ammonia-lyase 2	**46**	Unkown Protein Function	**0.99**
**44**	Unkown Protein Function	**46**	Unkown Protein Function	**1.00**
**3**	Cytokinin dehydrogenase 3	**47**	Abscisic acid 8'-hydroxylase 3	**0.87**
**6**	Ca2+-dependent lipid-binding protein CLB1/vesicle protein vp115/Granuphilin A contains C2 domain	**47**	Abscisic acid 8'-hydroxylase 3	**1.00**
**22**	60S ribosomal protein L7-3	**47**	Abscisic acid 8'-hydroxylase 3	**1.00**
**27**	60S ribosomal protein L7-4	**47**	Abscisic acid 8'-hydroxylase 3	**1.00**
**1**	pore-forming toxin-like protein Hfr-2	**48**	Omega-hydroxypalmitate O-feruloyl transferase	**1.00**
**9**	4-hydroxyphenylacetaldehyde oxime monooxygenase	**48**	Omega-hydroxypalmitate O-feruloyl transferase	**1.00**
**12**	PR6 protease inhibitor	**48**	Omega-hydroxypalmitate O-feruloyl transferase	**1.00**
**15**	Pathogenesis-related protein STH-2	**48**	Omega-hydroxypalmitate O-feruloyl transferase	**1.00**
**20**	Flavin-containing monooxygenase YUCCA6	**48**	Omega-hydroxypalmitate O-feruloyl transferase	**1.00**
**28**	Cytochrome P450 71B35	**48**	Omega-hydroxypalmitate O-feruloyl transferase	**1.00**
**43**	Unkown Protein Function	**48**	Omega-hydroxypalmitate O-feruloyl transferase	**0.99**
**3**	Cytokinin dehydrogenase 3	**49**	Vacuolar amino acid transporter 1	**0.88**
**19**	Vacuolar amino acid transporter 1	**49**	Vacuolar amino acid transporter 1	**0.98**
**31**	Cysteine-rich receptor-like protein kinase 10	**49**	Vacuolar amino acid transporter 1	**0.98**
**33**	Vacuolar amino acid transporter 1	**49**	Vacuolar amino acid transporter 1	**0.99**
**36**	Carbonic anhydrase chloroplastic	**49**	Vacuolar amino acid transporter 1	**0.98**
**37**	Galactinol--sucrose galactosyltransferase	**49**	Vacuolar amino acid transporter 1	**0.95**
**45**	Glutathione S-transferase zeta class	**49**	Vacuolar amino acid transporter 1	**0.99**
**1**	pore-forming toxin-like protein Hfr-2	**50**	Trans-cinnamate 4-monooxygenase	**0.99**
**9**	4-hydroxyphenylacetaldehyde oxime monooxygenase	**50**	Trans-cinnamate 4-monooxygenase	**0.99**
**15**	Pathogenesis-related protein STH-2	**50**	Trans-cinnamate 4-monooxygenase	**0.99**
**16**	Cytochrome P450 76C2	**50**	Trans-cinnamate 4-monooxygenase	**0.96**
**26**	Phenylalanine ammonia-lyase	**50**	Trans-cinnamate 4-monooxygenase	**0.97**
**28**	Cytochrome P450 71B35	**50**	Trans-cinnamate 4-monooxygenase	**0.99**
**43**	Unkown Protein Function	**50**	Trans-cinnamate 4-monooxygenase	**0.99**
**48**	Omega-hydroxypalmitate O-feruloyl transferase	**50**	Trans-cinnamate 4-monooxygenase	**0.98**
**8**	Stilbene synthase 3	**52**	Major allergen Pru av 1	**0.90**
**35**	F-box/LRR-repeat protein At3g48880	**53**	Heat shock protein 83	**0.86**
**51**	GDSL esterase/lipase At1g29670	**53**	Heat shock protein 83	**0.98**

**53**	Heat shock protein 83	**54**	Major allergen Pru av 1	**-1.00**
**35**	F-box/LRR-repeat protein At3g48880	**55**	Major allergen Pru ar 1	**-0.88**
**51**	GDSL esterase/lipase At1g29670	**55**	Major allergen Pru ar 1	**-0.98**
**54**	Major allergen Pru av 1	**55**	Major allergen Pru ar 1	**0.99**
**51**	GDSL esterase/lipase At1g29670	**56**	Major allergen Pru ar 1	**-0.98**
**54**	Major allergen Pru av 1	**56**	Major allergen Pru ar 1	**0.99**
**55**	Major allergen Pru ar 1	**56**	Major allergen Pru ar 1	**1.00**
**12**	PR6 protease inhibitor	**57**	Beta-amylase 3 chloroplastic	**0.96**
**18**	Protein WAX2	**57**	Beta-amylase 3 chloroplastic	**0.97**
**26**	Phenylalanine ammonia-lyase	**57**	Beta-amylase 3 chloroplastic	**0.85**
**29**	Eugenol synthase 1	**57**	Beta-amylase 3 chloroplastic	**0.98**
**39**	Eugenol synthase 1	**57**	Beta-amylase 3 chloroplastic	**0.98**
**40**	Protein WAX2	**57**	Beta-amylase 3 chloroplastic	**-0.94**
**51**	GDSL esterase/lipase At1g29670	**59**	23.6 kDa heat shock protein mitochondrial	**0.99**
**53**	Heat shock protein 83	**59**	23.6 kDa heat shock protein mitochondrial	**1.00**
**54**	Major allergen Pru av 1	**59**	23.6 kDa heat shock protein mitochondrial	**-1.00**
**55**	Major allergen Pru ar 1	**59**	23.6 kDa heat shock protein mitochondrial	**-0.99**
**56**	Major allergen Pru ar 1	**59**	23.6 kDa heat shock protein mitochondrial	**-0.99**
**4**	Unkown Protein Function	**60**	Major allergen Pru av 1	**0.96**
**5**	Stilbene synthase 4	**60**	Major allergen Pru av 1	**0.94**
**13**	Stilbene synthase 4	**60**	Major allergen Pru av 1	**0.94**
**21**	Stilbene synthase 4	**60**	Major allergen Pru av 1	**0.96**
**23**	Stilbene synthase 1	**60**	Major allergen Pru av 1	**0.95**
**32**	Stilbene synthase 4	**60**	Major allergen Pru av 1	**0.96**
**35**	F-box/LRR-repeat protein At3g48880	**60**	Major allergen Pru av 1	**-0.99**
**38**	Stilbene synthase 4	**60**	Major allergen Pru av 1	**0.96**
**52**	Major allergen Pru av 1	**60**	Major allergen Pru av 1	**0.93**
**56**	Major allergen Pru ar 1	**60**	Major allergen Pru av 1	**0.90**
**42**	Unkown Protein Function	**62**	Cationic peroxidase 1	**0.98**
**51**	GDSL esterase/lipase At1g29670	**63**	Chaperone protein ClpB 1	**1.00**
**55**	Major allergen Pru ar 1	**63**	Chaperone protein ClpB 1	**-0.98**
**56**	Major allergen Pru ar 1	**63**	Chaperone protein ClpB 1	**-0.98**
**35**	F-box/LRR-repeat protein At3g48880	**64**	18.6 kDa class III heat shock protein	**0.91**
**52**	Major allergen Pru av 1	**64**	18.6 kDa class III heat shock protein	**-0.97**
**53**	Heat shock protein 83	**64**	18.6 kDa class III heat shock protein	**0.99**
**54**	Major allergen Pru av 1	**64**	18.6 kDa class III heat shock protein	**-0.99**
**60**	Major allergen Pru av 1	**64**	18.6 kDa class III heat shock protein	**-0.90**

**61**	Uncharacterized membrane protein YOL092W	**64**	18.6 kDa class III heat shock protein	**-0.86**
**58**	Glucan endo-13-beta-glucosidase basic isoform	**65**	Germin-like protein subfamily 1 member 13	**1.00**
**62**	Cationic peroxidase 1	**65**	Germin-like protein subfamily 1 member 13	**1.00**
**8**	Stilbene synthase 3	**66**	Thaumatin-like protein	**0.91**
**17**	(+)-delta-cadinene synthase isozyme A	**66**	Thaumatin-like protein	**0.97**
**25**	Proteasome subunit alpha type-2-B	**66**	Thaumatin-like protein	**0.96**
**52**	Major allergen Pru av 1	**66**	Thaumatin-like protein	**0.98**
**58**	Glucan endo-13-beta-glucosidase basic isoform	**67**	Germin-like protein subfamily 1 member 15	**1.00**
**62**	Cationic peroxidase 1	**67**	Germin-like protein subfamily 1 member 15	**1.00**
**65**	Germin-like protein subfamily 1 member 13	**67**	Germin-like protein subfamily 1 member 15	**1.00**
**51**	GDSL esterase/lipase At1g29670	**68**	Unkown Protein Function	**0.99**
**53**	Heat shock protein 83	**68**	Unkown Protein Function	**0.99**
**54**	Major allergen Pru av 1	**68**	Unkown Protein Function	**-0.99**
**55**	Major allergen Pru ar 1	**68**	Unkown Protein Function	**-0.99**
**56**	Major allergen Pru ar 1	**68**	Unkown Protein Function	**-0.99**
**59**	23.6 kDa heat shock protein mitochondrial	**68**	Unkown Protein Function	**1.00**
**58**	Glucan endo-13-beta-glucosidase basic isoform	**69**	O-methyltransferase ZRP4	**1.00**
**62**	Cationic peroxidase 1	**69**	O-methyltransferase ZRP4	**1.00**
**65**	Germin-like protein subfamily 1 member 13	**69**	O-methyltransferase ZRP4	**1.00**
**67**	Germin-like protein subfamily 1 member 15	**69**	O-methyltransferase ZRP4	**1.00**
**51**	GDSL esterase/lipase At1g29670	**70**	Heat shock factor protein HSF30	**0.98**
**53**	Heat shock protein 83	**70**	Heat shock factor protein HSF30	**1.00**
**54**	Major allergen Pru av 1	**70**	Heat shock factor protein HSF30	**-1.00**
**59**	23.6 kDa heat shock protein mitochondrial	**70**	Heat shock factor protein HSF30	**1.00**
**63**	Chaperone protein ClpB 1	**70**	Heat shock factor protein HSF30	**0.98**
**64**	18.6 kDa class III heat shock protein	**70**	Heat shock factor protein HSF30	**0.99**
**68**	Unkown Protein Function	**70**	Heat shock factor protein HSF30	**0.99**
**17**	(+)-delta-cadinene synthase isozyme A	**71**	secretory protein putative	**0.93**
**25**	Proteasome subunit alpha type-2-B	**71**	secretory protein putative	**0.96**
**52**	Major allergen Pru av 1	**71**	secretory protein putative	**0.95**
**66**	Thaumatin-like protein	**71**	secretory protein putative	**0.98**
**61**	Uncharacterized membrane protein YOL092W	**72**	Pathogenesis-related protein R major form	**0.96**
**64**	18.6 kDa class III heat shock protein	**72**	Pathogenesis-related protein R major form	**-0.93**
**66**	Thaumatin-like protein	**72**	Pathogenesis-related protein R major form	**0.95**

**71**	secretory protein putative	**72**	Pathogenesis-related protein R major form	**0.97**
**58**	Glucan endo-13-beta-glucosidase basic isoform	**73**	Germin-like protein subfamily 1 member 15	**1.00**
**62**	Cationic peroxidase 1	**73**	Germin-like protein subfamily 1 member 15	**1.00**
**65**	Germin-like protein subfamily 1 member 13	**73**	Germin-like protein subfamily 1 member 15	**1.00**
**67**	Germin-like protein subfamily 1 member 15	**73**	Germin-like protein subfamily 1 member 15	**1.00**
**69**	O-methyltransferase ZRP4	**73**	Germin-like protein subfamily 1 member 15	**1.00**
**40**	Protein WAX2	**74**	Probable gibberellin receptor GID1L2	**-0.97**
**62**	Cationic peroxidase 1	**75**	Probable xyloglucan endotransglucosylase/hydrolase protein 23	**0.99**
**65**	Germin-like protein subfamily 1 member 13	**75**	Probable xyloglucan endotransglucosylase/hydrolase protein 23	**1.00**
**67**	Germin-like protein subfamily 1 member 15	**75**	Probable xyloglucan endotransglucosylase/hydrolase protein 23	**1.00**
**69**	O-methyltransferase ZRP4	**75**	Probable xyloglucan endotransglucosylase/hydrolase protein 23	**1.00**
**73**	Germin-like protein subfamily 1 member 15	**75**	Probable xyloglucan endotransglucosylase/hydrolase protein 23	**1.00**
**62**	Cationic peroxidase 1	**76**	Probable NADH dehydrogenase	**1.00**
**65**	Germin-like protein subfamily 1 member 13	**76**	Probable NADH dehydrogenase	**0.99**
**67**	Germin-like protein subfamily 1 member 15	**76**	Probable NADH dehydrogenase	**0.99**
**73**	Germin-like protein subfamily 1 member 15	**76**	Probable NADH dehydrogenase	**0.99**
**75**	Probable xyloglucan endotransglucosylase/hydrolase protein 23	**76**	Probable NADH dehydrogenase	**0.99**
**51**	GDSL esterase/lipase At1g29670	**77**	Heat shock cognate protein 80	**1.00**
**53**	Heat shock protein 83	**77**	Heat shock cognate protein 80	**0.99**
**56**	Major allergen Pru ar 1	**77**	Heat shock cognate protein 80	**-0.98**
**59**	23.6 kDa heat shock protein mitochondrial	**77**	Heat shock cognate protein 80	**1.00**
**61**	Uncharacterized membrane protein YOL092W	**77**	Heat shock cognate protein 80	**-0.83**
**63**	Chaperone protein ClpB 1	**77**	Heat shock cognate protein 80	**1.00**
**68**	Unkown Protein Function	**77**	Heat shock cognate protein 80	**1.00**
**70**	Heat shock factor protein HSF30	**77**	Heat shock cognate protein 80	**0.99**
**53**	Heat shock protein 83	**78**	Unkown Protein Function	**1.00**
**54**	Major allergen Pru av 1	**78**	Unkown Protein Function	**-1.00**
**55**	Major allergen Pru ar 1	**78**	Unkown Protein Function	**-0.99**
**56**	Major allergen Pru ar 1	**78**	Unkown Protein Function	**-0.99**

**59**	23.6 kDa heat shock protein mitochondrial	**78**	Unkown Protein Function	**1.00**
**63**	Chaperone protein ClpB 1	**78**	Unkown Protein Function	**0.99**
**64**	18.6 kDa class III heat shock protein	**78**	Unkown Protein Function	**0.98**
**68**	Unkown Protein Function	**78**	Unkown Protein Function	**1.00**
**70**	Heat shock factor protein HSF30	**78**	Unkown Protein Function	**0.99**
**77**	Heat shock cognate protein 80	**78**	Unkown Protein Function	**1.00**
**8**	Stilbene synthase 3	**80**	Expansin-A15	**-0.93**
**17**	(+)-delta-cadinene synthase isozyme A	**80**	Expansin-A15	**-0.97**
**25**	Proteasome subunit alpha type-2-B	**80**	Expansin-A15	**-0.95**
**35**	F-box/LRR-repeat protein At3g48880	**80**	Expansin-A15	**0.96**
**41**	Stilbene synthase 2	**80**	Expansin-A15	**-0.94**
**52**	Major allergen Pru av 1	**80**	Expansin-A15	**-0.99**
**66**	Thaumatin-like protein	**80**	Expansin-A15	**-1.00**
**72**	Pathogenesis-related protein R major form	**80**	Expansin-A15	**-0.93**
**58**	Glucan endo-13-beta-glucosidase basic isoform	**81**	Flavanone 3-dioxygenase	**1.00**
**62**	Cationic peroxidase 1	**81**	Flavanone 3-dioxygenase	**1.00**
**65**	Germin-like protein subfamily 1 member 13	**81**	Flavanone 3-dioxygenase	**1.00**
**67**	Germin-like protein subfamily 1 member 15	**81**	Flavanone 3-dioxygenase	**1.00**
**69**	O-methyltransferase ZRP4	**81**	Flavanone 3-dioxygenase	**1.00**
**76**	Probable NADH dehydrogenase	**81**	Flavanone 3-dioxygenase	**1.00**
**42**	Unkown Protein Function	**82**	RNA-binding protein 24	**0.97**
**58**	Glucan endo-13-beta-glucosidase basic isoform	**82**	RNA-binding protein 24	**0.99**
**65**	Germin-like protein subfamily 1 member 13	**82**	RNA-binding protein 24	**0.99**
**67**	Germin-like protein subfamily 1 member 15	**82**	RNA-binding protein 24	**0.99**
**69**	O-methyltransferase ZRP4	**82**	RNA-binding protein 24	**0.99**
**76**	Probable NADH dehydrogenase	**82**	RNA-binding protein 24	**0.99**
**81**	Flavanone 3-dioxygenase	**82**	RNA-binding protein 24	**1.00**
**42**	Unkown Protein Function	**83**	Ferredoxin--nitrite reductase chloroplastic	**0.99**
**71**	secretory protein putative	**83**	Ferredoxin--nitrite reductase chloroplastic	**0.99**
**72**	Pathogenesis-related protein R major form	**83**	Ferredoxin--nitrite reductase chloroplastic	**0.94**
**76**	Probable NADH dehydrogenase	**83**	Ferredoxin--nitrite reductase chloroplastic	**0.97**
**81**	Flavanone 3-dioxygenase	**83**	Ferredoxin--nitrite reductase chloroplastic	**0.95**
**82**	RNA-binding protein 24	**83**	Ferredoxin--nitrite reductase chloroplastic	**0.96**
**22**	60S ribosomal protein L7-3	**84**	Ankyrin-1	**0.76**

**42**	Unkown Protein Function	**84**	Ankyrin-1	**-0.97**
**75**	Probable xyloglucan endotransglucosylase/hydrolase protein 23	**84**	Ankyrin-1	**-0.96**
**51**	GDSL esterase/lipase At1g29670	**85**	DnaJ homolog subfamily B member 13	**1.00**
**55**	Major allergen Pru ar 1	**85**	DnaJ homolog subfamily B member 13	**-0.97**
**63**	Chaperone protein ClpB 1	**85**	DnaJ homolog subfamily B member 13	**1.00**
**68**	Unkown Protein Function	**85**	DnaJ homolog subfamily B member 13	**0.99**
**77**	Heat shock cognate protein 80	**85**	DnaJ homolog subfamily B member 13	**1.00**
**42**	Unkown Protein Function	**86**	Unkown Protein Function	**0.91**
**69**	O-methyltransferase ZRP4	**86**	Unkown Protein Function	**0.97**
**73**	Germin-like protein subfamily 1 member 15	**86**	Unkown Protein Function	**0.98**
**79**	Putative phosphoethanolamine N-methyltransferase 2	**86**	Unkown Protein Function	**-0.97**
**84**	Ankyrin-1	**86**	Unkown Protein Function	**-0.96**
**58**	Glucan endo-13-beta-glucosidase basic isoform	**87**	Cucumber peeling cupredoxin	**1.00**
**62**	Cationic peroxidase 1	**87**	Cucumber peeling cupredoxin	**1.00**
**65**	Germin-like protein subfamily 1 member 13	**87**	Cucumber peeling cupredoxin	**1.00**
**67**	Germin-like protein subfamily 1 member 15	**87**	Cucumber peeling cupredoxin	**1.00**
**69**	O-methyltransferase ZRP4	**87**	Cucumber peeling cupredoxin	**0.99**
**76**	Probable NADH dehydrogenase	**87**	Cucumber peeling cupredoxin	**1.00**
**81**	Flavanone 3-dioxygenase	**87**	Cucumber peeling cupredoxin	**1.00**
**82**	RNA-binding protein 24	**87**	Cucumber peeling cupredoxin	**1.00**
**83**	Ferredoxin--nitrite reductase chloroplastic	**87**	Cucumber peeling cupredoxin	**0.96**
**51**	GDSL esterase/lipase At1g29670	**88**	HSP90 co-chaperone putative	**1.00**
**55**	Major allergen Pru ar 1	**88**	HSP90 co-chaperone putative	**-0.98**
**56**	Major allergen Pru ar 1	**88**	HSP90 co-chaperone putative	**-0.98**
**63**	Chaperone protein ClpB 1	**88**	HSP90 co-chaperone putative	**1.00**
**68**	Unkown Protein Function	**88**	HSP90 co-chaperone putative	**0.99**
**77**	Heat shock cognate protein 80	**88**	HSP90 co-chaperone putative	**1.00**
**78**	Unkown Protein Function	**88**	HSP90 co-chaperone putative	**0.99**
**85**	DnaJ homolog subfamily B member 13	**88**	HSP90 co-chaperone putative	**1.00**
**61**	Uncharacterized membrane protein YOL092W	**89**	expressed protein	**0.99**
**64**	18.6 kDa class III heat shock protein	**89**	expressed protein	**-0.87**
**72**	Pathogenesis-related protein R major form	**89**	expressed protein	**0.94**
**77**	Heat shock cognate protein 80	**89**	expressed protein	**-0.87**
**17**	(+)-delta-cadinene synthase isozyme A	**90**	Epidermis-specific secreted glycoprotein EP1	**0.96**

**25**	Proteasome subunit alpha type-2-B	**90**	Epidermis-specific secreted glycoprotein EP1	**0.97**
**42**	Unkown Protein Function	**90**	Epidermis-specific secreted glycoprotein EP1	**0.94**
**52**	Major allergen Pru av 1	**90**	Epidermis-specific secreted glycoprotein EP1	**0.97**
**66**	Thaumatin-like protein	**90**	Epidermis-specific secreted glycoprotein EP1	**1.00**
**71**	secretory protein putative	**90**	Epidermis-specific secreted glycoprotein EP1	**0.99**
**72**	Pathogenesis-related protein R major form	**90**	Epidermis-specific secreted glycoprotein EP1	**0.96**
**42**	Unkown Protein Function	**91**	Germin-like protein subfamily 1 member 15	**0.98**
**58**	Glucan endo-13-beta-glucosidase basic isoform	**91**	Germin-like protein subfamily 1 member 15	**0.99**
**62**	Cationic peroxidase 1	**91**	Germin-like protein subfamily 1 member 15	**1.00**
**65**	Germin-like protein subfamily 1 member 13	**91**	Germin-like protein subfamily 1 member 15	**1.00**
**67**	Germin-like protein subfamily 1 member 15	**91**	Germin-like protein subfamily 1 member 15	**1.00**
**69**	O-methyltransferase ZRP4	**91**	Germin-like protein subfamily 1 member 15	**0.99**
**73**	Germin-like protein subfamily 1 member 15	**91**	Germin-like protein subfamily 1 member 15	**1.00**
**75**	Probable xyloglucan endotransglucosylase/hydrolase protein 23	**91**	Germin-like protein subfamily 1 member 15	**1.00**
**76**	Probable NADH dehydrogenase	**91**	Germin-like protein subfamily 1 member 15	**1.00**
**82**	RNA-binding protein 24	**91**	Germin-like protein subfamily 1 member 15	**0.99**
**87**	Cucumber peeling cupredoxin	**91**	Germin-like protein subfamily 1 member 15	**1.00**
**51**	GDSL esterase/lipase At1g29670	**92**	Heat shock 70 kDa protein	**1.00**
**55**	Major allergen Pru ar 1	**92**	Heat shock 70 kDa protein	**-0.98**
**56**	Major allergen Pru ar 1	**92**	Heat shock 70 kDa protein	**-0.98**
**63**	Chaperone protein ClpB 1	**92**	Heat shock 70 kDa protein	**1.00**
**68**	Unkown Protein Function	**92**	Heat shock 70 kDa protein	**0.99**
**77**	Heat shock cognate protein 80	**92**	Heat shock 70 kDa protein	**1.00**
**78**	Unkown Protein Function	**92**	Heat shock 70 kDa protein	**0.99**
**85**	DnaJ homolog subfamily B member 13	**92**	Heat shock 70 kDa protein	**1.00**
**88**	HSP90 co-chaperone putative	**92**	Heat shock 70 kDa protein	**1.00**
**58**	Glucan endo-13-beta-glucosidase basic isoform	**93**	Eugenol O-methyltransferase	**1.00**
**65**	Germin-like protein subfamily 1 member 13	**93**	Eugenol O-methyltransferase	**0.99**
**67**	Germin-like protein subfamily 1 member 15	**93**	Eugenol O-methyltransferase	**0.99**
**69**	O-methyltransferase ZRP4	**93**	Eugenol O-methyltransferase	**1.00**
**73**	Germin-like protein subfamily 1 member 15	**93**	Eugenol O-methyltransferase	**0.99**

**81**	Flavanone 3-dioxygenase	**93**	Eugenol O-methyltransferase	**0.99**
**82**	RNA-binding protein 24	**93**	Eugenol O-methyltransferase	**0.99**
**86**	Unkown Protein Function	**93**	Eugenol O-methyltransferase	**0.95**
**87**	Cucumber peeling cupredoxin	**93**	Eugenol O-methyltransferase	**0.99**
**91**	Germin-like protein subfamily 1 member 15	**93**	Eugenol O-methyltransferase	**0.99**
**17**	(+)-delta-cadinene synthase isozyme A	**94**	Unkown Protein Function	**0.96**
**25**	Proteasome subunit alpha type-2-B	**94**	Unkown Protein Function	**0.99**
**42**	Unkown Protein Function	**94**	Unkown Protein Function	**0.98**
**66**	Thaumatin-like protein	**94**	Unkown Protein Function	**0.97**
**71**	secretory protein putative	**94**	Unkown Protein Function	**0.98**
**80**	Expansin-A15	**94**	Unkown Protein Function	**-0.95**
**83**	Ferredoxin--nitrite reductase chloroplastic	**94**	Unkown Protein Function	**0.99**
**90**	Epidermis-specific secreted glycoprotein EP1	**94**	Unkown Protein Function	**0.98**
**58**	Glucan endo-13-beta-glucosidase basic isoform	**95**	Probable xyloglucan endotransglucosylase/hydrolase protein 23	**0.99**
**62**	Cationic peroxidase 1	**95**	Probable xyloglucan endotransglucosylase/hydrolase protein 23	**1.00**
**65**	Germin-like protein subfamily 1 member 13	**95**	Probable xyloglucan endotransglucosylase/hydrolase protein 23	**1.00**
**67**	Germin-like protein subfamily 1 member 15	**95**	Probable xyloglucan endotransglucosylase/hydrolase protein 23	**1.00**
**69**	O-methyltransferase ZRP4	**95**	Probable xyloglucan endotransglucosylase/hydrolase protein 23	**1.00**
**73**	Germin-like protein subfamily 1 member 15	**95**	Probable xyloglucan endotransglucosylase/hydrolase protein 23	**1.00**
**75**	Probable xyloglucan endotransglucosylase/hydrolase protein 23	**95**	Probable xyloglucan endotransglucosylase/hydrolase protein 23	**1.00**
**76**	Probable NADH dehydrogenase	**95**	Probable xyloglucan endotransglucosylase/hydrolase protein 23	**0.99**
**81**	Flavanone 3-dioxygenase	**95**	Probable xyloglucan endotransglucosylase/hydrolase protein 23	**0.99**
**87**	Cucumber peeling cupredoxin	**95**	Probable xyloglucan endotransglucosylase/hydrolase protein 23	**0.99**
**91**	Germin-like protein subfamily 1 member 15	**95**	Probable xyloglucan endotransglucosylase/hydrolase protein 23	**1.00**
**93**	Eugenol O-methyltransferase	**95**	Probable xyloglucan endotransglucosylase/hydrolase protein 23	**0.99**

**76**	Probable NADH dehydrogenase	**96**	DNA replication licensing factor MCM2	**-0.93**
**79**	Putative phosphoethanolamine N-methyltransferase 2	**96**	DNA replication licensing factor MCM2	**0.97**
**84**	Ankyrin-1	**96**	DNA replication licensing factor MCM2	**0.99**
**86**	Unkown Protein Function	**96**	DNA replication licensing factor MCM2	**-0.98**
**51**	GDSL esterase/lipase At1g29670	**97**	Heat shock cognate 70 kDa protein 2	**1.00**
**55**	Major allergen Pru ar 1	**97**	Heat shock cognate 70 kDa protein 2	**-0.98**
**56**	Major allergen Pru ar 1	**97**	Heat shock cognate 70 kDa protein 2	**-0.98**
**59**	23.6 kDa heat shock protein mitochondrial	**97**	Heat shock cognate 70 kDa protein 2	**0.99**
**63**	Chaperone protein ClpB 1	**97**	Heat shock cognate 70 kDa protein 2	**1.00**
**68**	Unkown Protein Function	**97**	Heat shock cognate 70 kDa protein 2	**0.99**
**77**	Heat shock cognate protein 80	**97**	Heat shock cognate 70 kDa protein 2	**1.00**
**78**	Unkown Protein Function	**97**	Heat shock cognate 70 kDa protein 2	**0.99**
**85**	DnaJ homolog subfamily B member 13	**97**	Heat shock cognate 70 kDa protein 2	**1.00**
**88**	HSP90 co-chaperone putative	**97**	Heat shock cognate 70 kDa protein 2	**1.00**
**92**	Heat shock 70 kDa protein	**97**	Heat shock cognate 70 kDa protein 2	**1.00**
**61**	Uncharacterized membrane protein YOL092W	**98**	Probable serine/threonine-protein kinase WNK4	**0.98**
**64**	18.6 kDa class III heat shock protein	**98**	Probable serine/threonine-protein kinase WNK4	**-0.85**
**77**	Heat shock cognate protein 80	**98**	Probable serine/threonine-protein kinase WNK4	**-0.87**
**89**	expressed protein	**98**	Probable serine/threonine-protein kinase WNK4	**0.99**
**53**	Heat shock protein 83	**99**	Expansin-A8	**1.00**
**54**	Major allergen Pru av 1	**99**	Expansin-A8	**-1.00**
**55**	Major allergen Pru ar 1	**99**	Expansin-A8	**-0.99**
**56**	Major allergen Pru ar 1	**99**	Expansin-A8	**-0.99**
**59**	23.6 kDa heat shock protein mitochondrial	**99**	Expansin-A8	**1.00**
**64**	18.6 kDa class III heat shock protein	**99**	Expansin-A8	**0.99**
**68**	Unkown Protein Function	**99**	Expansin-A8	**1.00**
**70**	Heat shock factor protein HSF30	**99**	Expansin-A8	**1.00**
**78**	Unkown Protein Function	**99**	Expansin-A8	**1.00**
**97**	Heat shock cognate 70 kDa protein 2	**99**	Expansin-A8	**0.98**
**52**	Major allergen Pru av 1	**100**	Inorganic phosphate transporter 1-4	**1.00**
**64**	18.6 kDa class III heat shock protein	**100**	Inorganic phosphate transporter 1-4	**-0.97**
**66**	Thaumatin-like protein	**100**	Inorganic phosphate transporter 1-4	**0.98**

**70**	Heat shock factor protein HSF30	**100**	Inorganic phosphate transporter 1-4	**-0.94**
**71**	secretory protein putative	**100**	Inorganic phosphate transporter 1-4	**0.96**
**72**	Pathogenesis-related protein R major form	**100**	Inorganic phosphate transporter 1-4	**0.97**
**80**	Expansin-A15	**100**	Inorganic phosphate transporter 1-4	**-0.98**
**90**	Epidermis-specific secreted glycoprotein EP1	**100**	Inorganic phosphate transporter 1-4	**0.97**
