# Supplementary material for: Transcriptome profiling of grapevine seedless segregants during berry development reveals candidate genes associated with berry weight
Source: BMC Plant Biol. 2016 Apr 26;16:104. doi: 10.1186/s12870-016-0789-1 (PMC4845426; doi:10.1186/s12870-016-0789-1)
Supplement: Additional file 15: Figure S6. — Network analysis of co-expressed genes among LB and SB segregants. In blue and red are represented DE genes that are part of the network, which respectively present or not significant correlation with component 1 of the PCA analysis, selected as candidate genes for berry weight. Lines in red and blue represent negative and positive correlations, respectively. (PDF 64 kb) [file 12870_2016_789_MOESM15_ESM.pdf]

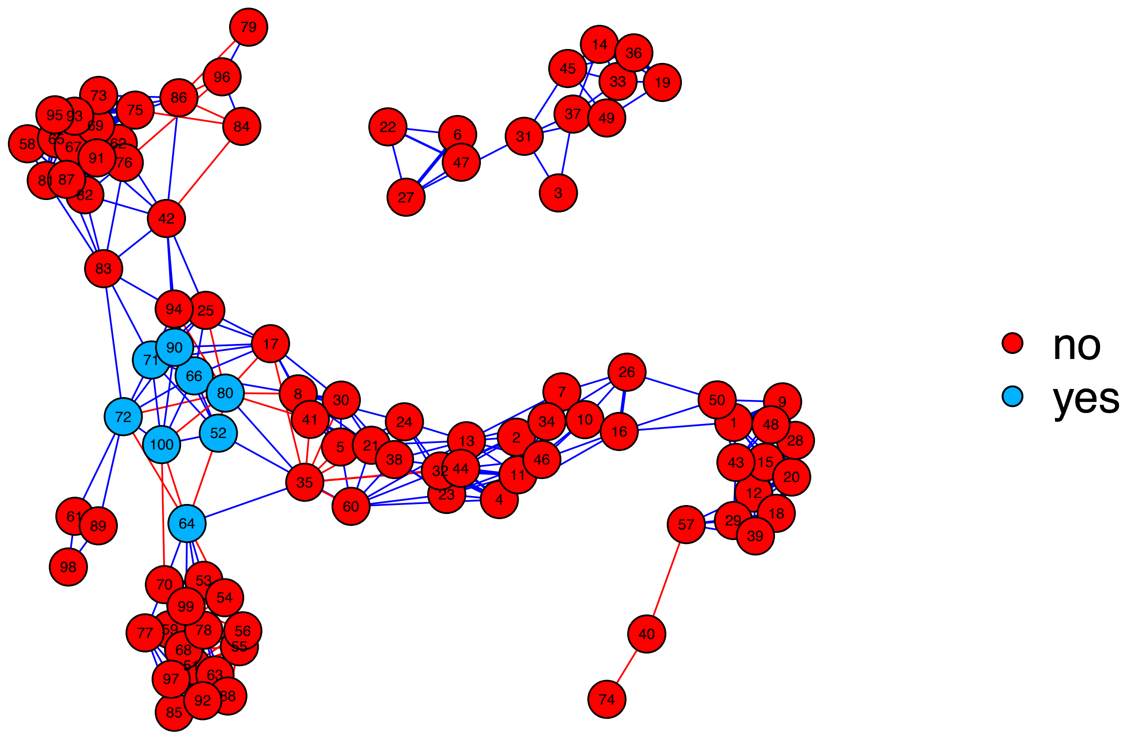

Figure S6. Network analysis of co-expressed genes among LB and SB segregants. In blue and red are represented DE genes that are part of the network, which respectively present or not significant correlation with component 1 of the PCA analysis, selected as candidate genes for berry weight. Lines in red and blue represent negative and positive correlations, respectively.
