# Supplementary material for: Transcriptome profiling of grapevine seedless segregants during berry development reveals candidate genes associated with berry weight
Source: BMC Plant Biol. 2016 Apr 26;16:104. doi: 10.1186/s12870-016-0789-1 (PMC4845426; doi:10.1186/s12870-016-0789-1)
Supplement: Additional file 16: Table S10. — Primers designed for the analysis of the expression level of the seven candidate genes, based on real-time qPCR. (PDF 63 kb) [file 12870_2016_789_MOESM16_ESM.pdf]

Table S10. Primers designed for the analysis of the expression level of the seven candidate genes, based on real-time qPCR.

geneID	Description	Type	Primer sequence (5'->3')	%GC	Tm (°C)
GSVIVG01008708001	TCPb (Possible complex-T subunit beta protein)	PF	AGACAGTGATTGACAGCCGAGTT	50	60
		PR	ATCCCTGCGTGGCTTTCTTCC		
GSVIVG01001971001	Ubiquitin	PF	GCCGCACTCTTGCTGATT	50	60
		PR	GATGGCTCAATAATACCACCAC		
GSVIVG01008305001	TF-bHLH93	PF	ACCCGGATTGAGATTTGC	50	60
		PR	TCTGAACAGGAAGCTTGCAC	50	
GSVIVG01009097001	TF-bHLH96	PF	CCGAGAAGAGCAGTAAGCAA	50	60
		PR	GTTGTCACCCATCATAGAGTC	48	
GSVIVG01015194001	Cytokinin dehydrogenase 3	PF	GGCTCTCTCATCACCAAACA	50	60
		PR	TCGTCCACTGTATGGCTTGT	50	
GSVIVG01010568001	Stilbene synthase 6	PF	CGCCAAGAGATTATCACTGCTG	50	60
		PR	GCATTTCTACACCGGAGGTTGT	50	
GSVIVG01033350001	TF-bHLH60	PF	GCAGAAAGGGCTAGGAGAGA	55	60
		PR	TAGGGATTGTACGTGGCTGA	50	
GSVIVG01021308001	GDSL esterase/lipase At1g29670	PF	TCAGGTCCATGCAGCAATAG	50	60
		PR	CTGTCATCTTCTTCCCCAGAG	52	
GSVIVG01035433001	17.9 kDa class II HSP	PF	GAATGGAGAGGAGGGTAGGC	60	60
		PR	CTCAAACGGTGACGGTCAGTA	55	

PF= Primer forward, PR= Primer reverse.
